# Correlation between the Antimicrobial Activity and Metabolic Profiles of Cell Free Supernatants and Membrane Vesicles Produced by *Lactobacillus reuteri* DSM 17938

**DOI:** 10.3390/microorganisms8111653

**Published:** 2020-10-24

**Authors:** Alessandro Maccelli, Simone Carradori, Valentina Puca, Francesca Sisto, Paola Lanuti, Maria Elisa Crestoni, Alba Lasalvia, Raffaella Muraro, Helena Bysell, Antonella Di Sotto, Stefan Roos, Rossella Grande

**Affiliations:** 1 Dipartimento di Chimica e Tecnologie del Farmaco, Sapienza Università di Roma, 00185 Rome, Italy; alessandro.maccelli@uniroma1.it (A.M.); mariaelisa.crestoni@uniroma1.it (M.E.C.); alba.lasalvia@uniroma1.it (A.L.); 2 Department of Pharmacy, “G. d’Annunzio” University of Chieti-Pescara, 66100 Chieti, Italy; 3 Center for Advanced Studies and Technology (CAST), “G. d’Annunzio” University of Chieti-Pescara, 66100 Chieti, Italy; valentina.puca@unich.it (V.P.); paola.lanuti@unich.it (P.L.); 4 Department of Medicine and Aging Science, “G. d’Annunzio” University of Chieti-Pescara, 66100 Chieti, Italy; 5 Department of Biomedical, Surgical and Dental Sciences, University of Milan, 20133 Milan, Italy; francesca.sisto@unimi.it; 6 Department of Medical, Oral and Biotechnological Sciences, “G. d’Annunzio” University of Chieti-Pescara, 66100 Chieti, Italy; raffaella.muraro@unich.it; 7 BioGaia, SE-103 64 Stockholm, Sweden; hb@biogaia.se; 8 Department of Physiology and Pharmacology “V. Erspamer”, Sapienza University of Rome, P.le Aldo Moro 5, 00185 Rome, Italy; antonella.disotto@uniroma1.it; 9 Department of Molecular Sciences, Swedish University of Agricultural Sciences, 750 07 Uppsala, Sweden; sr@biogaia.se

**Keywords:** *Lactobacillus reuteri*, *Limosilactobacillus reuteri*, cell free supernatant, metabolomics, extracellular and membrane vesicles, antimicrobial activity, biofilm, probiotics

## Abstract

The aim of the work is to assess the antimicrobial activities of Cell Free Supernatants (CFS) and Membrane Vesicles (MVs), produced by *Lactobacillus reuteri* DSM 17938, versus Gram-positive and Gram-negative bacteria and investigate their metabolic profiles. The Minimum Inhibitory Concentration was determined through the broth microdilution method and cell proliferation assay while the Minimum Bactericidal Concentration was determined by Colony Forming Units counts. The characteristics of the antimicrobial compounds were evaluated by pH adjustments, proteinase treatment, and size fractionation of the CFS. The cytotoxicity of CFS was tested on two human cell lines. A detailed snapshot of the *L*. *reuteri* metabolism was attained through an untargeted metabolic profiling by means of high resolution Fourier Transform Ion Cyclotron Resonance Mass Spectrometry (FT-ICR MS) coupled with Electrospray Ionization Source (ESI). The results showed (i) a greater efficacy of CFS and its fractions towards Gram-negative compared to Gram-positive bacteria; (ii) an antimicrobial effect related to pH-dependent compounds but not to MVs; (iii) a molecular weight < 3 KDa as well as an a non-proteinaceous nature of the antimicrobial compounds; and (iv) more than 200 and 500 putative metabolites annotated in MVs and supernatants, covering several classes of metabolites, including amino acids, lipids, fatty and organic acids, polyalcohols, nucleotides, and vitamins. Some putative compounds were proposed not only as characteristic of specific fractions, but also possibly involved in antimicrobial activity.

## 1. Introduction

Probiotics have been defined by the World Health Organization as “live microorganisms which, when administered in adequate amounts, confer a health benefit on the host”. Probiotics are proposed to act by multiple mechanisms through the production of e.g., organic acids, vitamins, antimicrobial substances, immune modulators, and neurotransmitters, which are useful for the regulation of intestinal transit and motility, the restoration of eubiosis, the resistance to the colonization of pathogens, etc. [1,2]. In recent years, the interest in probiotics has considerably increased due to the growing awareness of the importance of the gastrointestinal microbiota structure and composition.

*Lactobacillus* spp. represent one of the most widely dispensed probiotics [3] and may be involved in the prevention and treatment of gastrointestinal disorders like enteric infections and antibiotic-associated diarrhea [2,4]. In addition, *Lactobacillus* spp. have been shown to be effective in the prevention and treatment of urogenital diseases, bacterial vaginosis, atopic disease, and dental caries [4].

*Lactobacillus reuteri*, recently reclassified as *Limosilactobacillus reuteri* [5], colonizes the gastrointestinal tract of mammals and birds and are transmitted from one generation to another [6]. It has co-evolved with humans over millions of years [7]. In the past it was thought that the newborns are born sterile however, several studies detected bacterial DNA in first-pass meconium and amniotic fluid samples suggesting the presence or an early development of a microbiome in utero [8,9,10]. Newborns meet *L. reuteri* during the birthing process and through breastfeeding [6]. *L. reuteri* colonizes the gastrointestinal tract of humans [2,11]. As a member of gut microbiota, *L. reuteri* strains interact with the host in different ways, such as the stimulation of the immune system and the production of molecules or compounds that modulate the microbiota composition and prevent pathogen colonization [6]. Many studies demonstrated that several *L. reuteri* strains release various antimicrobial compounds capable of inhibiting the growth of pathogens in vitro [12,13]. Among those, the best characterized is reuterin, a mixture of different forms of 3-hydroxypropionaldehyde (3-HPA) endowed with an antimicrobial activity versus many bacterial pathogens [13]. Generally, *L. reuteri* strains are more resistant to reuterin than most other bacteria, suggesting that the production of reuterin could represent a defense strategy aimed to promote the species survival [6]. *L. reuteri* is capable of producing biofilm [14,15], a complex structure characterized by a community of microorganisms adhered to a surface and embedded in self-produced matrix of Extracellular Polymeric Substances (EPS), a mixture of macromolecules such as exopolysaccharides, proteins, and extracellular DNA (eDNA) and membrane vesicles [16,17]. Recently, it has been demonstrated that during its growth *L. reuteri* DSM 17938 produces Membrane Vesicles (MVs), also defined extracellular vesicles, in both planktonic (p) and biofilm (b) phenotypes [15,18,19].

It has also been demonstrated that the MVs produced by different strains of *Lactobacillus rhamnosus*, *Lactobacillus casei*, and *Lactobacillus plantarum* deliver effector molecules responsible for probiotic effects [20,21,22]. In addition, MVs released by *B. longum* alleviated a food allergy response in a mouse model, while *L. rhamnosus* MVs were shown to have a significant cytotoxic effect on hepatic cancer cells. Moreover, *L. casei* MVs contain proteins responsible of anti-apoptotic effects and *L. plantarum* MVs guarantee the protection of the host by inhibiting pathogen colonization. The described effects have often been observed with MVs but not with bacterial cells and an explanation of these results is probably due to the capability of MVs to cross the intestinal epithelial barrier and move to other parts of the body or interact with the host’s immune system [20,23].

Several studies demonstrated that MVs produced by microorganisms that are members of the gut microbiota travel within the human body delivering their content far from the producer microorganisms [24,25,26]. Moreover, the fusion of bacterial MVs with eukaryotic cell membranes has been distinctly demonstrated [27,28]. It is well known that many probiotic strains can release into the extracellular environment bioactive substances, such as biosurfactants, bacteriocins and antimicrobial peptides, that can inhibit the growth of pathogens. However, it has been speculated that each *Lactobacillus* strain is capable of producing its own peculiar compound. Therefore, the aim of the present study is the evaluation of the potential antimicrobial activity and the metabolic profile of MVs produced by *L. reuteri* DSM 17938 in planktonic and biofilm phenotypes. Since many studies have demonstrated the secretion of antimicrobial agents directly into the extracellular environment [29,30,31], we have also evaluated the antimicrobial activity and metabolic profile of the Cell Free Supernatant (CFS). In particular, the study of the metabolic profile was carried out both to identify possible candidate molecules responsible for antimicrobial activity and to get a better understanding of the metabolic content in the vesicles or released to the extracellular environment.

An untargeted metabolomics approach, based on direct infusion Fourier Transform Ion Cyclotron Resonance Mass Spectrometry (FT-ICR MS) coupled with Electrospray Ionization (ESI), has been exploited to achieve maximum metabolome coverage on biofilm and planktonic MVs and CFS, in the attempt to, for the first time, ascribe the antimicrobial properties to their chemical diversity. This method has widely shown to provide a fast and accurate metabolic fingerprint of (moderately) polar compounds in complex biological matrixes through in-depth chemical characterization covering a large variety of molecular families [32]. The qualitative description of the metabolic profile has been clustered by van Krevelen diagrams, which allow the visualization of the most populated classes of metabolites.

Furthermore, the possible cytotoxic effects of MVs and CFS have been evaluated in human noncancerous epithelial cells of the biliary tract (H69 intrahepatic cholangiocytes), a representative in vitro model of the gastrointestinal system, due to the common embryological origin with liver and intestinal epithelial cells [33,34,35]. In order to characterize the cytotoxicity of MVs and CFS on non-gut tissues and human bronchial epithelial BEAS-2B cells, widely used toxicological studies on inhaled toxicants, were also included [36].

## 2. Material and Methods

### 2.1. Bacterial Strains and Culture Conditions

*Lactobacillus reuteri* DSM 17938 [37] kindly provided by BioGaia AB (Stockholm, Sweden), was used in the study. The strain was plated on DeMan, Rogosa, and Sharpe agar (MRSA; Oxoid Limited, Basingstoke, Hampshire, UK), and incubated at 37 °C for 24 h in an anaerobic atmosphere (Anaerogen Pak Jar, Oxoid Ltd.). Different media and growth conditions were selected for the different bacterial species used in the study. In particular, *Escherichia coli*, *Staphylococcus aureus*, and *Pseudomonas aeruginosa* were cultured on Mueller-Hinton agar (MHA; Oxoid Ltd.) for 24 h in aerobic conditions; *Fusobacterium nucleatum* was cultured on Fastidious Anaerobe Agar (FAA; Lab M, Heywood, UK) and 5% (*v*/*v*) of defibrinated horse sterile blood (Oxoid Ltd.) for 48 h in anaerobiosis (Anaerogen Pak Jar, Oxoid Ltd.); and *Streptococcus mutans* was cultured on Columbia agar (CA; Oxoid Ltd.) and 5% (*v*/*v*) of defibrinated horse sterile blood (Oxoid Ltd.) for 24 h at 5% CO_2_. The isolated strains have been previously used in other research studies [33]. The study did not require ethical approval because all isolates were obtained as a part of routine diagnostic microbiology investigations, however, patients gave informed consent for further scientific studies.

### 2.2. Isolation and Size Fractionation of L. reuteri Cell Free Supernatant

The bacteria were inoculated in DeMan, Rogosa, and Sharpe broth (MRSB; Oxoid Ltd.) and incubated overnight at 37 °C in anaerobic atmosphere under shaking at 90 rpm. After incubation, each culture was diluted in MRSB to an optical density (OD_600_) of 0.10, corresponding to approximately 10^7^ CFU/mL, and incubated at 37 °C for 24 h. The Cell Free Supernatant indicated as SurP (Planktonic Supernatant) was obtained after centrifugation for 20 min at 4000× *g* at 4 °C and subsequently filtered through 0.22 µm cellulose membrane filters (Corning, New York, NY, USA). The presence of Membrane Vesicles (MVs) was determined and quantified by using Polychromatic Flow Cytometry (PFC) according to Puca et al. [38]. To separate the MVs from other free components, the SurP was concentrated by using Amicon^®^ Ultra-15 10K (10,000 MWCO) Centrifugal Filter Devices (Merck KGaA, Darmstadt, Germany) according to the manufacturer’s recommendations. Briefly, 15 mL of the SurP were centrifuged for 45 min at 4000× *g* at room temperature to reach the volume 150–300 μL. The eluted sample was called SurE (Eluted Supernatant) 10K, while the sample held by the filter (MVs) named SurM (Membrane Supernatant) 10K, was diluted in MRSB until the volume of 4 mL. SurP, SurE 10K, and SurM 10K were subsequently filtered through a cellulose membrane filter of 0.22 µm pore size (Corning, New York, USA) and tested for the antimicrobial activity versus *E. coli*, *P. aeruginosa*, *F. nucleatum*, *S. aureus*, and *S. mutans*. The absence of MVs in SurE 10K was further confirmed by using PFC.

### 2.3. Biofilm Formation Assay and MVs Isolation

*L. reuteri* biofilm was developed into 90 mm diameter Petri dishes (Corning Incorporated, New York, NY, USA) as previously described and MVs were isolated from the planktonic and biofilm phenotypes as previously described [15]. The isolated pMVs and bMVs were quantified by using PFC according to Puca et al. [38] and used for the evaluation of the antimicrobial activity against the microorganisms aforementioned, as well as for the analysis of the metabolic profiles.

### 2.4. Characterization of the Bacterial Strains Used

The antimicrobial activity of SurP, SurE 10K, and SurM 10K was evaluated on 5 bacterial species. The antimicrobial susceptibility pattern was determined by using the Kirby–Bauer disc diffusion method [39]. The discs containing fixed concentrations of different antimicrobials were supplied by Oxoid.

### 2.5. Determination of Minimum Inhibitory Concentration and Minimum Bactericidal Concentration of SurP, SurE 10K, and SurM 10K

The Minimum Inhibitory Concentration (MIC) and the Minimum Bactericidal Concentration (MBC) were determined using the broth microdilution method in 96-well polystyrene microtitre plates (Eppendorf, Hamburg, Germany) and CFU counting. The results obtained were confirmed by the hydrosoluble formazan XTT (sodium-2,3-bis-[2-methoxy-4-nitro-5-sulfophenyl]-2*H*–tetrazolium-5-carboxanilide) XTT assay as previously demonstrated [40]. Briefly, *E. coli*, *S. aureus*, and *P. aeruginosa* were grown in Tryptic Soy Broth (TSB; Oxoid Ltd., Hampshire, UK) for 16 h at 37 °C under shaking conditions at 125 rpm. The overnight broth cultures were resuspended in Mueller–Hinton broth (MHB; Oxoid) to an optical density at 550 nm (OD_550_) of 0.8 corresponding to 1–5 × 10^8^ Colony-Forming Units (CFU)/mL. The broth cultures were then diluted in MHB to 1–5 × 10^5^ CFU/mL per well. *F. nucleatum* was grown in Fastidious Anaerobe Broth (FAB; Lab M, Heywood, UK) for 18 h at 37 °C under shaking conditions at 125 rpm in anaerobiosis condition. The overnight broth cultures were resuspended in FAB to an optical density at 600 nm (OD_600_) of 0.15 corresponding to 1–5 × 10^7^ CFU/mL [41], diluted in FAB to 1–5 × 10^5^ CFU/mL per well. *S. mutans* was grown in Brain Heart Infusion Broth (BHIB, Oxoid Ltd.) plus 2% (*w*/*v*) glucose for 18 h at 37 °C at 5% CO_2_. The overnight broth cultures were resuspended to an OD_600_ of 0.80 corresponding to 1.0 × 10^8^ CFU/mL and then diluted in BHIB plus glucose to 1–5 × 10^5^ CFU/mL per well.

Considering the fact that the MIC values of SurP were perfectly reproducible or, in any case, very close, as in the case of *E. coli*, among the strains belonging to the same species, we decided to test the antimicrobial activity of SurE 10K and SurM 10K exclusively on the reference strains, adding the SurP as positive control.

SurP, SurE 10K, and SurM 10K were diluted in MRS broth in the range of 5–50 µL per well. The plates were incubated at 37 °C for 24 h for *E. coli*, *S. aureus*, *P. aeruginosa*, and *S. mutans* and for 48 h for *F. nucleatum*. The MIC was defined as the lowest concentration without visible growth. The MBC was defined as the lowest concentration that gave a reduction of CFU with 99.9% compared to the initial inoculum. One hundred microliters have been picked up by the wells corresponding to concentrations ≥MIC and plated. Controls consisting of (i) bacterial broth cultures grown in the appropriate media, (ii) bacterial broth cultures grown in the appropriate media with the addition of 50 µL of MRSB, (iii) the appropriate media with the addition of SurP or SurE 10K or SurM 10K at different concentrations, (iv) MRSB and the appropriate broths, and (v) just the appropriate media were added to the experiment. Three independent experiments were performed in triplicate.

### 2.6. XTT Metabolic Assay

The MIC and MBC were confirmed by using XTT metabolic assay (Cell Proliferation Kit II XTT, Roche Diagnosis, Mannheim, Germany) according to the manufacturer’s recommendations, as previously described [40]. The colorimetric assay is based on the cleavage of the yellow tetrazolium salt XTT, added to the sample, to form an orange formazan dye by metabolic active cells. The conversion occurs in the presence of viable cells. The formazan production can be determined by using a plate reader. In brief, the XTT labeling reagent and the electron-coupling reagent were thawed in a waterbath at 37 °C and mixed to obtain a clear solution, immediately before use. To confirm the MIC values evaluated by eye, in each well, containing the different broth cultures plus different concentrations of SurP or SurE 10K, or SurM 10K, 50 µL of the XTT labeling mixture were added to each sample (final XTT concentration 0.3 mg/mL). The microplates containing *E. coli*, *S. aureus*, and *P. aeruginosa* and *S. mutans* were then incubated for 2 h, while *F. nucleatum* for 4 h in the proper atmosphere conditions previously described. The absorbance was then read at 490 nm using a microplate reader (Synergy H1 Multi-Mode Reader, BioTek, Winooski, VT, USA). The MIC in microplate reader, was defined as the lowest concentration of SurP or SurE 10K or SurM 10K that inhibited bacterial growth by 50% [42].

**Figure 1:**
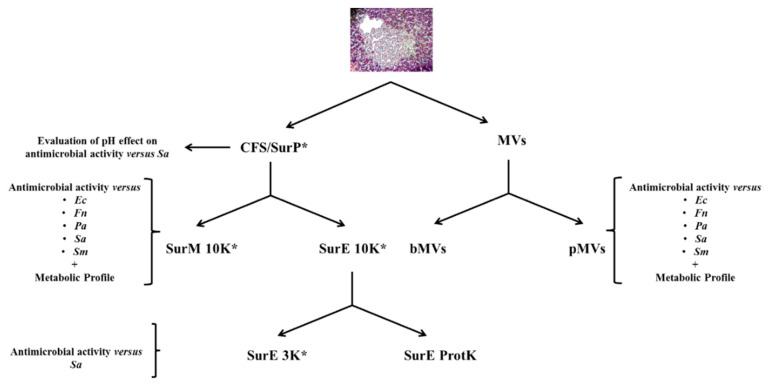
Flowchart of this study. CFS/SurP = Cell Free Supernatant/Planktonic Supernatant; MVs = Extracellular Vesicles; bMVs = Membrane Vesicles of Biofilm phenotype; pMVs = Membrane Vesicles of Planktonic phenotype; Ec = *Escherichia coli*; Fn = *Fusobacterium nucleatum*; Pa = *Pseudomonas aeruginosa*; Sa = *Staphylococcus aureus*; Sm = *Streptococcus mutans*; SurE 10K = Fractionated Planktonic Supernatant eluted by Amicon Ultra-15 10K centrifugal filter Devices; SurM 10K = Membrane Planktonic Supernatant obtained above the Amicon Ultra-15 10K centrifugal filter Devices; SurE 3K = Fractionated Planktonic Supernatant eluted by Amicon^®^ Ultra-2 3K Centrifugal Filter Devices; SurE ProtK = Fractionated Planktonic Supernatant eluted by Amicon Ultra-15 10K centrifugal filter Devices and treated with 1 mg/mL of proteinase K; * = Cytotoxicity evaluation versus human cell lines.

Moreover, to investigate if the antimicrobial activity was associated with larger protein-like compounds, SurE 10K was both treated with proteinase K and further fractionated by using 3K columns (Figure 1). One mL of SurE 10K was treated with Proteinase K (Sigma-Aldrich, Milan, Italy) 1 mg/mL at 37 °C for 2 h and then at 95 °C for 1 min to inactivate the enzyme. Moreover, SurE 10K was further fractionated by using Amicon^®^ Ultra-2 3K (3000 NMWL) Centrifugal Filter Devices (Merck, KGaA) as indicated by the manufacturer’s. The obtained samples were indicated as SurE + K (SurE 10K treated with Proteinase K) and SurE 3K (SurE 10K treated with 3K columns). Finally, the samples were sterilized through a cellulose membrane filter of 0.22 µm pore size (Corning, NY, USA) and tested for the antimicrobial activity as previously indicated.

Moreover, to exclude the effect of pH in the antimicrobial activity, *S. aureus* ATCC 29213 was used as test strain. The pH was measured at the inoculum (T0) and after 24 h of incubation (T24). In particular, SurP was adjusted at pH 7.0 by using 6 M of NaOH while MHB was adjusted to pH 4.3 (the same pH of SurP) by using 2 M of acetic acid. The following conditions were examined:

(i) *S. aureus* grown in MHB plus SurP (dil. 1:4); (ii) *S. aureus* grown in MHB plus SurP (dil. 1:4) pH 7.0 (iii) *S. aureus* grown in MHB (control); and (iv) *S. aureus* grown in MHB plus MRSB (dil. 1:4) to demonstrate that MRSB do not affect *S. aureus* growth (control).

### 2.7. Determination of Minimum Inhibitory Concentration and Minimum Bactericidal Concentration of pMVs and bMVs

The MIC and MBC of bMVs and pMVs were evaluated on the bacterial strains as mentioned before. The number of MVs isolated by the two phenotypes of *L. reuteri* DSM 17938 were determined as previously described [38]. The pMVs and bMVs were added to the broth cultures in the following amounts of 1 × 10^5^, 2 × 10^5^, 3 × 10^5^, 4 × 10^5^, and 1 × 10^6^ MVs per well. The protocol for the determination of the MIC and MBC was set up following the indications reported by the Clinical & Laboratory Standards Institute (CLSI) guidelines by replacing the drug with different amounts of bMVs or pMVs.

### 2.8. Samples Preparation and FT-ICR Untargeted Metabolomics

All reagents and standards were supplied with MS grade purity by commercial sources (Sigma Aldrich, Milan, Italy) and used as received. 2 mL of EtOH were added to pMVs and bMVs and then the pellet rupture was ultrasound-assisted at 45 ± 5 KHz for 4 min in a Sonica 2200M (Soltec, Milan, Italy), thus obtaining a white, turbid emulsion. Filtration through GHP Acrodisc 0.450 µm filters allowed the removal of macromolecular residues, i.e., polymers of lipid residues, proteins, or nucleic acid macrostructures known to interfere with MS investigations. A 10 µL aliquot of each solution was diluted in 10 mL of methanol to be analyzed by ESI FT-ICR mass analyses [43]. 1% *v*/*v* of formic acid was added as ionization dopant in positive mode. Additionally, both SurE 10K and SurM 10K supernatants obtained by ultracentrifugation were analyzed. Filtration of 1 µL was carried out as described for the MVs solutions. Assessment of possible ionic suppression phenomena revealed 1:1000 dilution in methanol as the most suitable final dilution for direct injection into the mass spectrometer. As previously mentioned, also in these samples, 1% *v*/*v* of formic acid was used as ionization dopant.

Samples were measured on a Bruker BioApex FT-ICR mass spectrometer (Bremen, Germany) equipped with an Electrospray Ionization (ESI) source, a 4.7 T superconducting magnet, and an Infinity Cell. Analyte solutions were directly infused at a rate of 120 µL h^−1^. A m/z 50–1050 range was investigated, and 150 spectra were co-added with an acquisition size of 1M points. Both positive and negative polarity modes were investigated. Spectra were frequency-to-m/z internally calibrated with respect of internal standards: Arginine and hesperidin in ESI(+), and glutamic acid and caffeoylquinic acid in ESI(−). All mass measurements refer to monoisotopic ions possessing a signal/noise ratio higher than 4. Peaks annotations were obtained by comparison between exact mass and experimental mass submitting extracted calibrated m/z to Masstrix [44]. Chloride, sodium, and potassium adducts, acquired in either negative or positive ion mode, were considered in a mass deviation range of ± 3 ppm referring to Kegg [45], HMDB [46], and LipidMaps [47] databases. The selected libraries are free and currently publicly available [48]. A large amount of the bulk chemical formulas contained in these databases, referring to exact mass values, makes them appropriate for metabolites research in different biological fields, including microbial metabolomics [49].

Further metabolites annotation assessments were achieved by: (i) Collision Induced Dissociation (CID) experiments, conducted in a Paul Ion Trap (Esquire 3000+, Bruker Daltonics, Bremen, Germany), that allows a comparison of experimental fragmentation data with those contained in Metlin database library [50]; (ii) comparison between experimental and theoretical isotopic pattern by means of Isotope Pattern tool (Bruker Daltonics, Bremen); and (iii) confirmation of molecular formula by means of Molecular Formula Finder tool, contained in ChemCalc [51]. The chemical formulas identified for each sample were clustered in two-dimensional van Krevelen diagrams [52] by plotting the molar hydrogen to carbon (H/C) ratio vs the molar oxygen to carbon (O/C) ratio for each data point. Thereby, a qualitative representation of the identified compounds in the main families of expressed metabolites can be obtained. The common and unique features from the SurE and SurM and the pMVs and bMVs samples have been displayed in a Venn diagram, while the frequency histograms of CH, CHN, CHNO, CHNOP, CHNOS, CHO, CHOP, and CHOS have been depicted in order to evaluate the elemental distribution. For each sample, redundant molecular formulas were removed in order to avoid overestimate the same compounds revealed with different ionization agents (i.e., H^+^, Na^+^, and K^+^).

### 2.9. Cytotoxicity Studies in Human Cell Lines

Cytotoxicity of bacterial vesicles (SurM 10K), medium (MRSB), and supernatants (SurP, SurE 10K, and SurE 3K) was assessed in two human epithelial cell lines, H69 and BEAS-2B, through the MTT assay, applying long-term exposure protocols of 24 h and 48 h (Figure S6). H69 cholangiocytes, kindly provided by Prof. G. Alpini (Indiana University School of Medicine, Indianapolis, IN, USA) and Prof. R. Mancinelli (Department of Anatomical, Histological, Forensic and Orthopedic Sciences, Sapienza University of Rome), were grown as previously described [34]. BEAS-2B cells, obtained from Sigma-Aldrich (Sigma-Aldrich, St. Louis, MO, USA), were cultivated under standard conditions (37 °C and 5% CO_2_) using a 1:1 mixture of LHC-9 and RPMI-1640 media, supplemented with 1% penicillin and streptomycin, 1% L-glutamine, and 10% FBS. To perform the cytotoxicity assay, 2 × 10^4^ cells were seeded in each well of 96-well microplates and allowed to grow for 24 h. Thereafter, they were treated with progressive volumes of the tested samples and then incubated for another 24 or 48 h. After treatment, the cytotoxicity was detected by the MTT assay [53]. Three independent experiments were carried out in triplicate. A change in cell viability due to the treatment was evaluated by comparison of cell viability in the treatment and in the vehicle control. A treatment was considered cytotoxic when giving ≥30% inhibition of cell viability.

### 2.10. Statistical Analysis

The differences in the means of the results between untreated and treated bacterial strains were evaluated by Student’s *t*-test. The probability value of *p* ≤ 0.05 was considered significantly different. Analysis of cytotoxicity data (expressed as mean ± standard error) was performed by GraphPad Prism TM 6.00 software (GraphPad Software, San Diego, CA, USA). The significance in the cytotoxic effects of the tested samples compared control was evaluated by one-way ANOVA plus Dunnett’s Multiple Comparison Post Test (GraphPad Prism TM 6.00, GraphPad Software, San Diego, CA, USA). The IC_50_ values were obtained from the concentration-response curve, according to a previous method [34].

## 3. Results

### 3.1. Evaluation of L. reuteri Biofilm Formation and Characterization of the Bacterial Strains Used in the Study

*L. reuteri* DSM 17938 developed a biofilm after 24 h of incubation (data not shown) similar to the biofilm-producing *L. reuteri* strains previously studied. The bacterial strains, used in the present study and corresponding to reference and clinical strains of *E. coli*, *S. aureus*, *P. aeruginosa*, *F. nucleatum*, and *S. mutans*, were characterized for their clinical isolation and susceptibility patterns versus the antimicrobial drugs more commonly used in therapies as reported in Table 1.

### 3.2. Determination of Minimum Inhibitory Concentration and Minimum Bactericidal Concentration of bMVs and pMVs

By adding different amounts of pMVs and bMVs it was demonstrated that MVs did not have any antimicrobial activities versus the bacterial species tested (data not shown).

### 3.3. Determination of the MIC and MBC of SurP, SurE 10K, and SurM 10K Versus the Different Bacterial Strains Used in the Study

**Figure 2:**
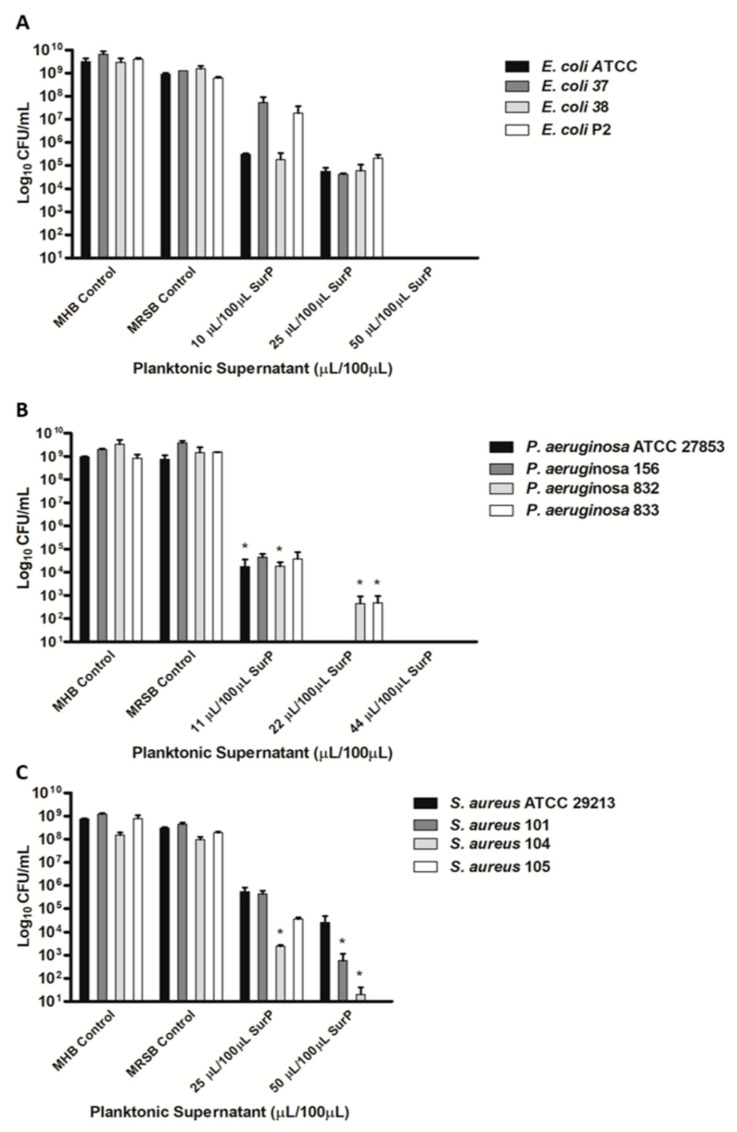
Determination of the MBC of SurP versus *E. coli* (**A**); *P. aeruginosa* (**B**); and *S. aureus* (**C**) through Colony-Forming Units (CFU) counts. The CFU counts have been evaluated at concentrations corresponding to the MIC and higher than the MIC of SurP. Data are presented as the mean of three replicates of three independent experiments. * *p* < 0.05 vs. the initial inoculum.

**Figure 3:**
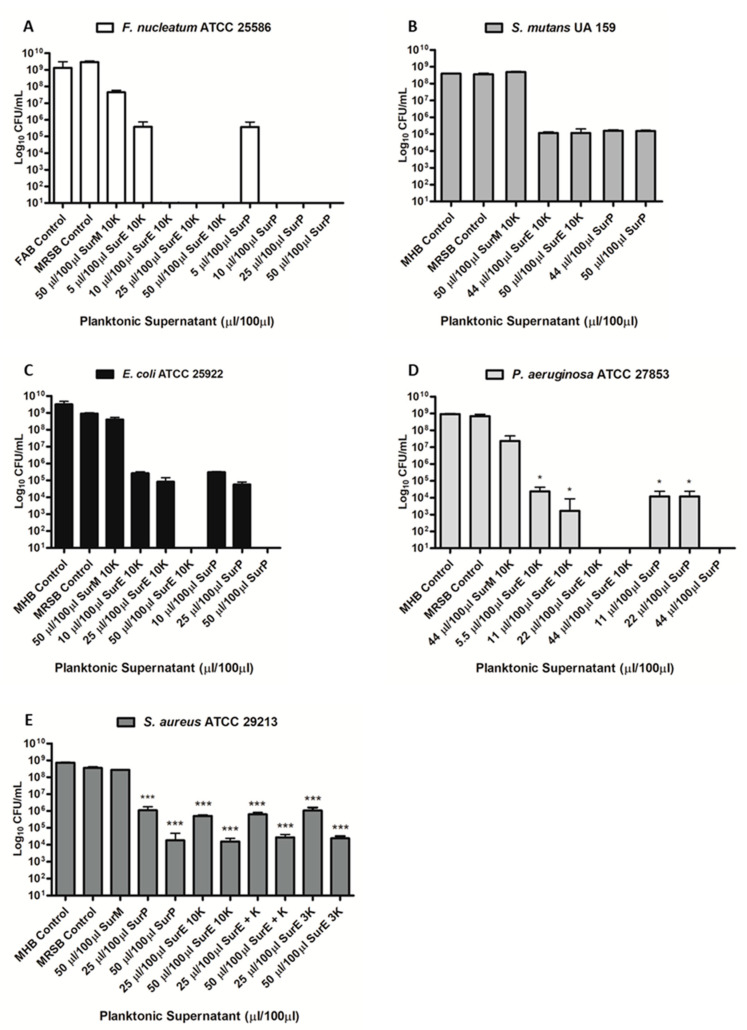
Determination of the MBC of SurP, SurM 10K, and SurE 10K versus *F. nucleatum* ATCC25586 (**A**); *S. mutans* UA159 (**B**); *E. coli* ATCC25922 (**C**); *P. aeruginosa* ATCC27853 (**D**); and *S. aureus* ATCC29213 (**E**) through CFU counts. The CFU counts have been evaluated at concentrations corresponding to the MIC and higher than the MIC. Data are presented as the mean of three replicates of three independent experiments. * *p* < 0.05 vs. the initial inoculum; and *** *p* < 0.001 vs. the initial inoculum.

The MIC of the total CFS (SurP; Figure 1) and its fractions (SurM and SurE) was determined by both the broth microdilution method (Table 1 and Table 2) and XTT assay. It was shown that SurP inhibited growth at dilutions 1:20 (MIC of 5 µL/100 µL) versus *F. nucleatum*; dilution 1:10 (MIC of 10 µL/100 µL) versus two strains of *E. coli*, and similarly, dilution 1:9 (MIC of 11 µL/100 µL) were recorded against *P. aeruginosa* reference and clinical strains. On the contrary, SurP showed an inhibition of the growth at dilution 1:4 (MIC of 25 µL/100 µL) versus *S. aureus* and two *E. coli* strains and dilution 1:4.5 (MIC of 22 µL/100 µL) versus *S. mutans* UA 159, respectively (Table 1). Regarding the evaluation of the MBC, SurP showed a MBC at ≥50 µL/100 µL versus *E. coli*, *S. aureus*, and *S. mutans*, and at 22 µL/100 µL and 10 µ/100 µL, versus *P. aeruginosa* and *F. nucleatum*, respectively (Table 1, Figure 2 and Figure 3). However, a CFU reduction was detected, as in the case of *P. aeruginosa* ATCC 27853 and *S. aureus* 104 in which a CFU a ~1-log_10_ decrease in cell count was detected at 25 µL/100 µL and 5.5 µL/100 µL, respectively (Figure 3D,E). The data obtained demonstrated a greater efficacy of SurP towards Gram-negative bacterial species rather than towards Gram-positive species as also confirmed by the MBC values, regardless of the pattern of resistance to antimicrobial drugs. In particular, the evaluation of the CFU demonstrated a ~1-log_10_ decrease in cell count at 25 µL for *S. aureus* 104, a multidrug resistant strain, however, the CFU counts confirm the MIC evaluated by the broth microdilution method and XTT assay as shown in Figure 2 and Figure 3. To determine the molecular weight of the compound/compounds having the antimicrobial activity the SurP was size fractionated and the fractions of SurM >10 KDa and SurE <10 KDa were obtained.

The MIC and MBC were evaluated against the reference strains belonging to each bacterial species and SurP was inserted as a positive control. SurM 10K did not show any antimicrobial activity, on the contrary SurE 10K displayed the same antimicrobial activity of SurP (Table 2), therefore, the candidate compound/compounds with an antimicrobial activity should have a molecular weight lower than 10 kDa. The 10K filter device excluded also the extracellular vesicles free in the planktonic supernatant (SurP), whose characteristics have been previously determined [15,38].

### 3.4. Evaluation Assay of pH on SurP and Proteinase K on SurE 10K Antimicrobial Activity

To investigate the impact of pH on the antimicrobial activity, we measured the pH at T_0_ and after 24 h of incubation of different samples of *S. aureus* ATCC 25922 which was chosen as a test strain.

The results showed that SurP diluted at 1:4 inhibits the growth of *S. aureus* ATCC 25922, while neutralized SurP allow the growth of the microorganism. Since the acidification of MHB (pH 5.2) allows the growth of *S. aureus*, we can speculate the existence of a compound/compounds having an antimicrobial activity, probably due to the ionization/protonation state which makes them pH-dependent. Moreover, to investigate if the antimicrobial activity could be associated with protein-like compounds (e.g., bacteriocins), we added Proteinase K to the SurE 10K and, in addition, we fractionated the SurE 10K by using a 3K column to exclude proteins with a molecular weight greater than 3 kDa. The data obtained showed that both SurE 10K treated with Proteinase K and SurE 3K showed a MIC at 25 µL/100 µL and a MBC ≥ 50 µL/100 µL. Therefore, the efficacy of the sample was preserved demonstrating that the component with the antimicrobial activity is not likely a proteinaceous compound.

### 3.5. Metabolic Fingerprint

**Figure 4:**
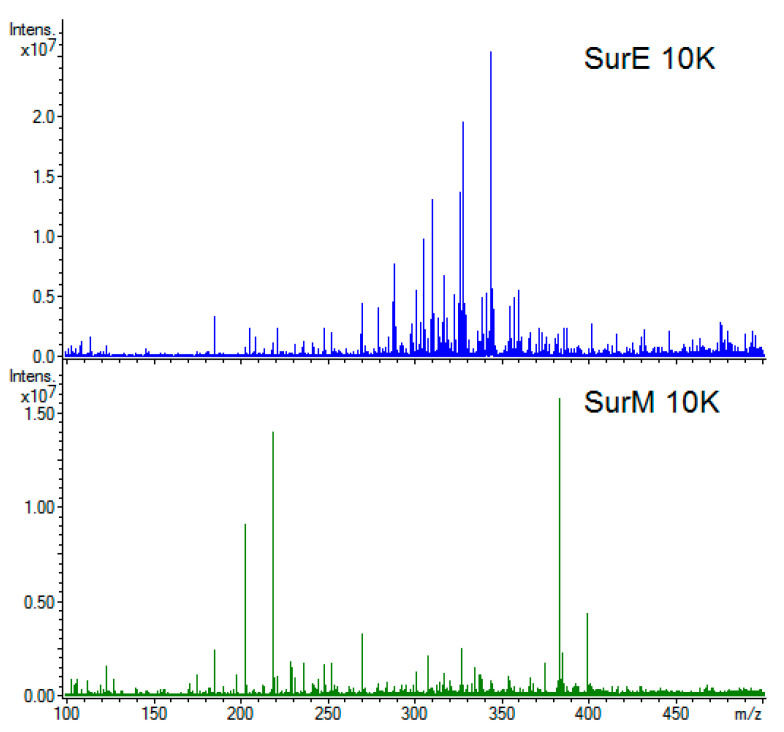
Representative mass spectra for SurE 10K (**upper** panel) and SurM 10K (**lower** panel) supernatants in the m/z 100–450 mass range.

Next, we determined the chemical composition of both bacterial vesicles and supernatants by direct infusion ESI FT-ICR MS and investigated possible correlations to the antimicrobial activity. Representative examples of ESI(+) FT-ICR spectra are shown in Figure 4 for SurE 10K (blue profile) and SurM 10K (green profile). Figures S1 and S2 show exemplary high-resolution mass spectra of SurE 10K and SurM 10K obtained in positive and negative polarity mode, respectively. Overall, a total amount of 1029 molecular formulas were identified in the two supernatants (SurE 10K and SurM 10K), whereas the analyses of vesicles (bMVs and pMVs) were allowed to assign 345 molecular formulas (Table 3). Here, the application of direct ESI injection coupled to FT-ICR MS allowed an annotation of a large miscellany of compounds, avoiding time-consuming preliminary chromatographic separations.

**Figure 5:**
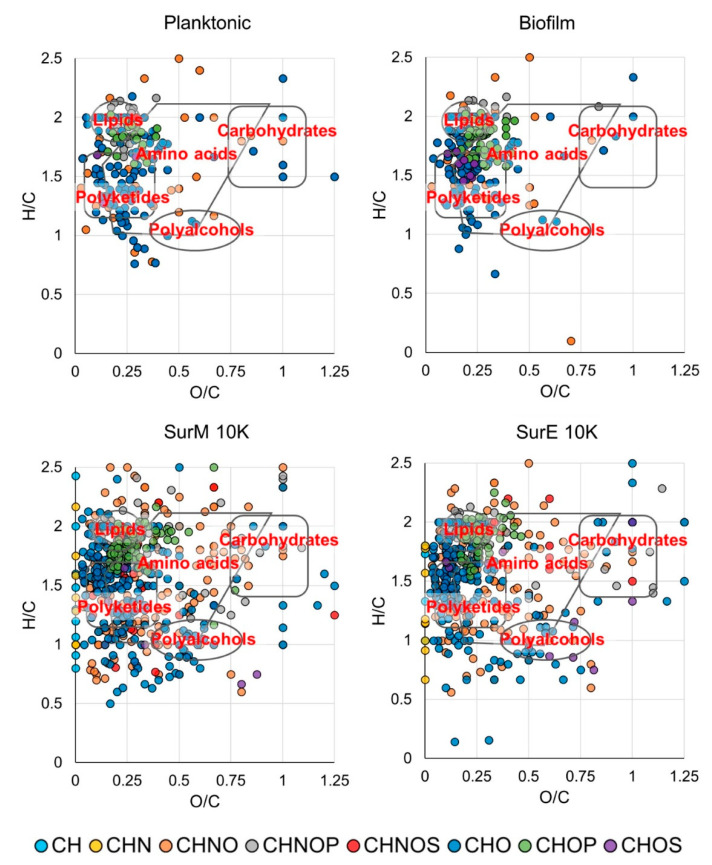
Van Krevelen diagrams of planktonic/biofilm vesicles extracts (**upper** panels) and SurM 10K/SurE 10K supernatants (**lower** panel).

The chemical space of SurM 10K appears to be more populated than its counterpart SurE 10K with respect to the already known compounds available in the databases, above all presenting a wider diversity of metabolites at a high molecular weight. Several compounds assayed by CID experiments have provided orthogonal information with accurate mass determination, thus supplying additional structural information in the absence of commercial standards. An example is shown in Figure S3 where a CID assay on peak at m/z 299 allowed to unequivocally annotate this signal as deprotonated hydroxy stearic acid. The complete list of assigned elemental formulas is reported in Tables S1–S4, along with the theoretical and experimental m/z ratios, mass deviation (ppm), ion abundance, and putative compound annotations. The employment of van Krevelen diagrams (vKDs), a visualization tool commonly used in metabolomics to assist information recovery, has allowed to more easily visualize molecular classes and compare the investigated chemical spaces. In Figure 5, vKDs of vesicles and supernatants are reported in upper and lower panels, respectively. All samples cover numerous classes of compounds, including lipids, polyketides, amino acids, and polyalcohols as the most populated areas. SurE 10K and SurM 10K showed a relevant presence of further classes of metabolites, including as example lipids, carbohydrates, and polyketides also found by other authors [53,54,55]. The lipids class mainly comprises: (i) Medium chain linear fatty acids, as deprotonated palmitic and myristic acid; (ii) lipids with biosurfactant activity, like phosphocholine PC(O-16:0/18:0) and acyltrehaloses DAT(19:0/24:0) found in SurE 10K; and (iii) lipopeptides, such as oleoyl glycine recorded in SurM 10K. In addition, SCFAs and derivatives produced during carbohydrates fermentation processes have been identified, including valeric (SurM 10K) and hydroxy-valeric acids (pMVs). A significant diversity of organic acids has also emerged in negative mode ionization with deprotonated lactic, citric, and malic acids, discovered in both supernatants and quinic acid found in bMVs and pMVs.

### 3.6. Cytotoxicity Studies in Human Cell Lines

Under our experimental conditions, the bacterial medium MRSB gave a significant biological cytotoxicity (about 42% cell viability reduction with respect to the control) in H69 cholangiocytes starting from the concentration of 30 μL/100 μL after 24 h exposure (Figure S4A). The cytotoxicity of MRSB was enhanced by a prolonged exposure of 48 h, achieving a 36% and 38% reduction in cell viability compared to the control at concentrations of 10 and 20 μL/100 μL (Figure S4A). BEAS-2B cells were highly susceptible to MRSB cytotoxicity, being the sample already cytotoxic (about 46% cell viability reduction compared to the control) at 2 μL/100 μL after 24 h (Figure S5A). According to this behavior, the IC_50_ of MRSB in H69 was about 10-fold higher than that in BEAS-2B cells (Table 4). A similar trend was also found for SurP, SurM 10K/MRSB, SurE 10K, and SurE 3K, which produced a comparable or slightly lower cytotoxicity than MRSB in both cell lines under both time-exposures (Figures S4 and S5). Accordingly, their IC_50_ values did not significantly differ in neither of the cell lines and time-schedule (Table 4). Conversely, when SurM 10K was assessed as a RPMI-1640 dilution, its cytotoxicity was markedly reduced in both cell lines, resulting in non-toxic up to 40 μL and 10 μL/100 μL in H69 and BEAS-2B cells after 24 h, respectively (Figures S4D and S5D). A prolonged exposure of 48 h increased the cytotoxicity of the sample, which significantly affected cell viability starting from 10 and 5 μL/100 μL in H69 and BEAS-2B cells, respectively (Figures S4D and S5D). Comparing IC_50_ values, SurM 10K/RPMI-1640 resulted in up to 2- and 10-fold less toxicity than SurM 10K/MRSB in H69 and BEAS-2B cells, respectively (Table 4).

## 4. Discussion

The data obtained in the present study demonstrated that *L. reuteri* DSM 17938 can produce substances endowed with antimicrobial activity, which are released in the extracellular environment. It is well known that, lactobacilli yield lactic acid and other organic acids, such as acetic, propionic, and phenyl-lactic acids, which contribute to the environmental pH decrease, thus inhibiting the proliferation of pathogens. Moreover, lactobacilli can also produce different antimicrobial compounds of low molecular weight as hydrogen peroxide, carbon dioxide, ethanol, diacetyl, and acetaldehyde, as well as high-molecular mass metabolites such as bacteriocins [56]. Recently, it has been shown that *L. reuteri* DSM 17938 produces MVs during its growth, generating both planktonic and biofilm phenotypes: pMVs and bMVs, whose physicochemical characteristics have been previously determined [15,17,38], and which have been tested here for their potential antimicrobial activity. In particular, we tested the potential antimicrobial activity of both MVs isolated by ultracentrifugation and filtration and MVs released by *L. reuteri* DSM 17938 in the extracellular environment.

The present data are in line with a previous report showing that both MVs and pellets from *L*. *reuteri* ATCC 23272 do not contain detectable antimicrobial compounds, for example bacteriocins [57]. Therefore, it is likely that MVs produced by *L. reuteri* DSM 17938 have a different role in the gut like the modulation of the immune system or cell-signaling or biofilm formation, therefore further studies should be performed to investigate the role of MVs produced by *L. reuteri* DSM 17938 during planktonic or biofilm cultivation. On the contrary, CFS (SurP) and especially its fraction SurE 10K derived from *L. reuteri* DSM 17938 displayed antimicrobial activity. The antimicrobial compounds contained in these fractions showed greater efficacy towards Gram-negative bacteria rather than towards Gram-positive bacteria. In fact, SurP and Sur E 10K displayed MIC values in the range 5–11 µL/100 µL (except for two clinical strains of *E. coli*) *versus E. coli*, *P. aeruginosa*, and *F. nucleatum*, and MIC of 22 and 25 µL/100 µL versus *S. mutans* and *S. aureus*, respectively. We investigated, the antimicrobial activity of SurP and its fractions on both reference and clinical strains belonging to the above-mentioned species, some of which were multi-drug resistant, to demonstrate that the efficacy of CFS is independent of the characteristics of the strain in terms of drug resistance patterns. The activity of CFS versus both reference and clinical isolates supported the hypothesis that the efficacy is related to the species and not to the individual strains.

The results obtained versus *P. aeruginosa* do not differ much from the results obtained by Aminnezhad et al., who demonstrated the antimicrobial activity of CFS produced by *L. casei* and *L. rhamnosus* against *P. aeruginosa* (MIC of 62.5 µL/mL) whose antimicrobial activity has been hypothesized to be associated with the presence of lactic acid, acetic acid, and hydrogen peroxide [58]. Moreover, Alakomi et al., (2000) demonstrated that lactic acid, in addition to exerting its antimicrobial effect due to a pH decrease, also acts as a membrane-permeabilizing of Gram-negative bacterial outer membrane thus favoring the entry of other antimicrobial agents [59]. Knysh and colleagues evaluated the effect of CFS of *Bifidobacterium bifidum* and *L. reuteri* DSM 17938 against *E. coli* and *P. aeruginosa* [60] demonstrating that CFS containing metabolites, obtained when the microorganism was grown with or without supplementation with glycerol and glucose, showed good antimicrobial activity against *E. coli* and *P. aeruginosa*. Differently, here we exclude that the antimicrobial agents produced by *L. reuteri* DSM 17938 are associated or delivered by MVs, but rather are freely released to the extracellular environment. The maintenance of the antimicrobial activity of the SurE 10K, after treatment with proteinase K and 3K columns confirmed that the substances with antimicrobial activity in SurP were not protein-like compounds and had a molecular less than 3 kDa. This information also suggests that the antimicrobial effect could be associated with a synergistic activity of a milieu of several substances contained in the SurP, as previously hypothesized by others [59].

The effect of the pH on the antimicrobial activity versus *S. aureus* ATCC 29213, used as test strain, confirmed that the induced acidic environment did not cause the inhibition of bacterial growth but probably affected the activity of the antimicrobial compounds. In fact, we demonstrated that SurP, when added to MHB, inhibited the growth of *S. aureus.* Conversely, the acidification of MHB till pH 4.3 as well as the neutralization till pH 7.0 allowed the growth of the microorganisms. These data suggested a pH-dependent antimicrobial activity. On the other hand, Poppi et al., displayed that *L. reuteri* supernatant showed bactericidal activity versus *E. coli* O157:H7, whereas a pH adjustment (alkalinization) induced a decrease of antimicrobial activity of the supernatant. The authors demonstrated no direct relationship between the amount of lactic acid and the degree of antimicrobial efficacy, confirming that the antimicrobial activity of lactobacilli was not only related to the organic acids production [61].

In line with the pH-dependent effect (although it is not the only hypothesized mechanism) of the antibacterial activity, usually ascribed to the aliphatic organic acids content, when human epithelial cells were also exposed to non-buffered solutions of bacterial vesicles and supernatants, a marked cytotoxicity occurred (data not shown) likely as a consequence of an intolerable acid stress related to the medium. As expected, after physiological buffering, the cytotoxicity of the tested samples was highly reduced especially in H69 cells. Accordingly, other authors reported the need of a pH adjustment for cell free supernatants from *Lactobacillus* spp. in order to avoid that pH variation could affect the response of human monocytic THP-1 cells [62]. The higher tolerance of cholangiocytes with respect bronchial epithelial cells could be due to their enzymatic and antioxidant defenses, which ensure cell survival from the injury of toxic metabolites in bile and/or hepatic blood [63]. On the other hand, airway epithelial cells have been reported to be cells with a short half-life with a slight tolerance to oxidative injury [64]. The concentration range here tested is in line with other studies on *Lactobacillus* spp. vesicles or supernatants [62,65]. In both cell lines, the cytotoxicity profile of the supernatants SurP, SurE 10K, and SurE 3K and SurM 10K/MRSB were found similar to that of MRSB, thus suggesting that the bacterial medium can contain some cytotoxic components (beef extracts, yeast extract, and sorbitan monooleate) for human cells that can interfere with the test compounds. Indeed, a replacement of MRSB with RPMI-1640 significantly lowered the cytotoxicity of SurM 10K, especially in BEAS-2B cells. Therefore, further studies aimed at improving the cultivation conditions of *L. reuteri* by the replacement of MRSB with more suitable alternatives represent an important point to be addressed in order to exploit the antimicrobial properties of SurM 10K, SurE 10K, and SurE 3K, retaining a safe profile in human cells.

The analysis of the metabolic profiles, carried out on the one hand to identify possible candidate molecules responsible of the antimicrobial activity of SurE 10 K, and on the other to better understand the metabolic content of pMVs and bMVs to highlight possible differences of such structures in the two phenotypes but also with the SurP, showed interesting data. The production of MVs as well as their content is the result of active metabolism in bacteria, therefore, the study of the metabolic profile of MVs, particularly in the two phenotypes, represents valuable information on the possible activities that such lipidic structures, released by probiotic strain, can perform in the host.

Notably, reuterin and its derivatives, which were shown to exhibit an inhibitory activity against non-probiotic Gram-positive and Gram-negative bacteria [66,67], lack in all samples. This result is likely a direct consequence of the absence of glycerol in the medium used, which prevents the formation of 3-HPA and derivatives, the so-called reuterin system, by bacterial metabolism [68].

The relative frequency distributions histograms of the identified molecular formulas are detailed in Figure S7. Interestingly, all samples contain a major number of CHO species (49% in bMVs and 55% in pMVs extracts), followed by CHNO (more hits, 27%, in SurE 10K), CHOP (more entries, 22%, in bMVs), and CHNOP (more hits, 14%, in SurM 10K). Differences between samples have been also highlighted by Venn diagrams showing that (only) 67 hits (20%) of the total elemental features are shared between pMVs and bMVs (Figure S8, upper panel) and 73 entries (7%) between SurE 10K and SurM 10K (Figure S8, lower panel).

Among the metabolites shared by the two supernatants, essential amino acids and derivatives, as protonated proline, leucine, arginine, and *N*-acetyl-tryptophan may be included. Conversely, protonated aspartame and sodiated tryptophan have been revealed exclusively in SurE 10K [29,69]. Some non-essential amino acids and small peptides, like cyclo(Ala-Val) and glutathione have also been recorded in SurE 10K as protonated and potassiated adducts, respectively. Moreover, SurE 10K has shown the presence of proline betaine, whose high concentration has been alleged to adverse cardiovascular diseases [70]. The non-proteic amino acid carnitine has been detected as potassiated and chlorinated adducts in SurM 10K and SurE 10K, respectively, while lacking in MVs extracts. Carnitine is already known for its contribution in bacterial metabolism to fatty acid transport into mitochondria [71,72].

Interestingly, both bMVs and pMVs extracts have shown the presence of sulfur amino acid derivatives and protonated lipoyllysine, known to play a key role in post-translational protein lipoylation reactions [73]. Moreover, CHNO components of both supernatants contain biogenic amines and their derivatives, including putrescine and dopamine glucuronide, end product of lactic acid bacteria metabolism [74], both identified in SurM 10K, and histamine, detected only in SurE 10K. Conversely, no representative compound of this class has been identified in vesicles extracts.

Putrescine and tyramine were proposed by Colosimo et al., to be important bacterial metabolites interacting with histamine receptor (gut-brain axis) [75]. These results are also in accordance with the previous report from Greifová et al., who described a different pattern of biogenic amines within a set of *L. reuteri* strains [56]. Histamine, among other low-molecular mass molecules, can modulate specific host immune and physiological responses. The secretion of histamine has been related to the innate immunity suppression by intestinal bacteria through TNF production inhibition via the H_2_ receptor, which increased cAMP and PKA activity, and reduced activation of MEK/ERK MAPK signaling [76]. Furthermore, histamine-containing probiotics could show anti-inflammatory effects inhibiting the production of pro-inflammatory cytokines (IL-1 and 12) and could act as a neurotransmitter impacting on the enteric nervous system [77]. Lastly, phenyllactid acid was reported to be the most potent and naturally occurring agonist of hydroxycarboxylic acid subtype 3 receptor (HCA_3_) in immune cells (e.g., monocytes) and an antibacterial metabolite in LAB (Lactic Acid Bacteria)-fermented foods. Conversely, HCA_1_ is activated by lactic acid and HCA_2_ by butyrate and 3-hydroxybutyrate [78].

Overall, organic acids (lactic acid, phenyllactic acid), biogenic amines (histamine, tyramine, putrescine), neurotransmitters (GABA, dopamine), vitamins and their derivatives (A, E, D, pantothenic acid), fatty acids and their hydroxyl- or keto-derivatives, antibiotics (macrocyclic lactones, aminoglycosides, macrolides, cyclic ionophores), antioxidants (glutathione, *N*-acetylcysteine, vitamin E derivatives), and microbial surfactants (3-*O*-L-rhamnosyl-3-hydroxydecanoyl-3-hydroxydecanoic acid and methyl 13-sophorosyloxydocosanoate) have been found to characterize the vesicles and supernatants isolated by *L. reuteri* DSM 17938 as well as antimicrobial activity and other biological properties (anti-inflammatory effects, immunomodulation, regulation of enteric musculature function, survival of other probiotic species). Numerous metabolites have been collected in Tables S1–S4 and most of them can participate towards antimicrobial activity as also previously reported by other studies on different strains of *L. reuteri* [2,30,56,60]. Furthermore, in agreement with Georgieva et al., the antimicrobial compounds present in this matrix are pH-dependent being active only at acidic pH values in which, probably, the indissociate form is more membrane- or cell wall-permeant [79].

## 5. Conclusions

In the last few years, the compounds released/produced by probiotics have attracted attention for their potential use for clinical, food, and industrial applications. In particular, MVs may deliver beneficial nutritional compounds such as proteins, vitamins, neurotransmitters precursors, as well as antimicrobial substances that reach host cells and play several roles that include the modulation of the immune host system, the protection of pathogens colonization, the interaction with the nervous systems, and other organs affecting the hosts in an efficient manner. Consequently, the use of purified and well characterized components derived by MVs or CFS might represent an alternative for clinical applications, particularly in infection treatment.

Direct infusion electrospray ionization coupled with high-resolution mass spectrometry was confirmed as a fast and sensitive tool for an extensive investigation of complex mixtures, and to have potential in antimicrobial drug research like in the present study on CSFs and MVs originated by *L. reuteri* DSM 17938. The high variety and wide range of secondary metabolites simultaneously detected, including amino acids, polyalcohols, fatty acids, and signaling molecules, could provide a more precise snapshot of the actual physiological state of the cell. This qualitative description could provide a useful benchmark to relate the complex microbial metabolomics of *L. reuteri* with its healthy biological effects, being not only related to the antimicrobial properties. The data obtained in the present research work represent the starting point for more in-depth studies aimed at the use of CFS or MVs derived from probiotic strains, used alone or in combination with probiotic bacterial cells, as an innovative strategy used as food supplement, for the treatment of several pathologies in clinical field.

## Figures and Tables

**Table 1:** List of the bacterial strains characterized for: (i) Clinical isolation, (ii) antimicrobial susceptibility pattern by disk diffusion testing against the drugs commonly used in therapy, (iii) Minimum Inhibitory Concentration (MIC) and Minimum Bactericidal Concentration (MBC) of SurP. AML, amoxycillin; AMP, ampicillin; CAZ, ceftazidime; CEC, cefaclor; CFM, cefixime; CLA, clarithromycin; CIP, ciprofloxacin; CN, gentamicin; CRO, ceftriaxone; CXM, cefuroxime; ERY, erythromycin; F, nitrofurantoin; FLO, flomoxef; FOS, fosfomycin; FOX, cefoxitin; IPM, imipenem; KAN, kanamycin; LEV, levofloxacin; LNZ, linezolid; MNZ, metronidazole; NET, netilmicin; NOR, norfloxacin; PEN, penicillin G; PRL, piperacillin; RD, rifampicin; SXT, trimethoprim-sulfamethoxazole; TE, tetracycline. S, sensitive; I, intermediate; R, resistant. SurP = Total planktonic supernatant, pEVs included.

Bacterial Strains	Clinical Isolation	Antimicrobial Susceptibility	SurP MIC/MBC (µL/100 µL)
*E. coli* ATCC 25922	Clinical isolate	PRL^R^, AML^R^	10/50
*E. coli* 37	Urinary sample	CEC^R^	25/50
*E. coli* 38	Urinary sample	CRO^R^, CEC^R^, PRL^R^, CFM^R^, AML^R^, LEV^R^	10/50
*E. coli* P2	Urinary sample	PRL^R^, CEC^I^	25/50
*F. nucleatum* ATCC 25586	Oral cavity	MNZ^s^, PEN^s^	5/10
*S. aureus* ATCC 29213	Wound	ERY^S^, TE^S^, NET^S^, LEV^S^, FOX^S^, LNZ^S^, RD^S^, CN^I^	25/≥50
*S. aureus* 101	Vaginal swab	ERY^S^, TE^S^, NET^S^, LEV^S^, FOX^S^, LNZ^S^, RD^S^, CN^S^	25/50
*S. aureus* 104	Pharyngeal swab	ERY^R^, TE^S^, NET^I^, LEV^R^, FOX^R^, LNZ^S^, RD^S^, CN^S^	25/50
*S. aureus* 105	Urinary sample	ERY^S^, TE^S^, NET^S^, LEV^S^, FOX^S^, LNZ^S^, RD^S^, CN^S^	25/50
*S. mutans* UA 159	Dental caries	AMP^S^	22/>50
*P. aeruginosa* ATCC 27853	Blood culture	AMP^R^, CAZ^S^, CXM^R^, CN^S^ F^R^, LEV^S^, NOR^S^, SXT^R^	11/22
*P. aeruginosa* 156	Urinary sample	AMP^R^, CAZ^I^, CXM^R^, CN^S^ F^R^, LEV^S^, NOR^S^, SXT^R^	11/22
*P. aeruginosa* 832	Urinary sample	AMP^R^, CAZ^I^, CXM^R^, CN^I^ F^R^, LEV^S^, NOR^S^, SXT^R^	11/22
*P. aeruginosa* 833	Urinary sample	AMP^R^, CAZ^S^, CXM^R^, CN^S^ F^R^, LEV^S^, NOR^S^, SXT^R^	11/22

**Table 2:** Evaluation of the MIC and MBC of SurM 10K = just vesicles (>10K); SurE 10K = fractionated planktonic supernatant (<10K), and SurP = Total planktonic supernatant (pMVs included) versus *E. coli* ATCC25922, *S. aureus* ATCC 29233; *S. mutans* UA159; *P. aeruginosa* ATCC 27853; and *F. nucleatum* ATCC 25586.

Bacterial Strains	SurM 10K MIC (µL/100 µL)	SurM 10K MBC (µL/100 µL)	SurE 10K MIC (µL/100 µL)	SurE 10K MBC (µL/100 µL)	SurP MIC (µL/100 µL)	**SurP** **MBC** **(µL/100 µL)**
*E. coli* ATCC 25922	≥50	≥50	10	50	10	50
*S. aureus* ATCC 29213	≥50/	≥50	25	50	25	≥50
*S. mutans* UA 159	≥50	50	44	>50	44	>50
*P. aeruginosa* ATCC 27853	44	>44	5.5	22	11	22
*F. nucleatum* ATCC 25586	≥50	≥50	5	10	5	10

**Table 3:** Number of molecular formulas assigned by Electrospray Ionization (ESI) Fourier Transform Ion Cyclotron Resonance Mass Spectrometry (FT-ICR MS).

Sample	ESI(+)	ESI(−)	Total
SurE 10K	304	205	489
SurM 10K	446	204	619
Biofilm vesicles	193	49	219
Planktonic vesicles	160	50	192

**Table 4:** IC_50_ values of the tested samples in human epithelial H69 cholangiocytes and bronchial BEAS-2B cells after 24 and 48 h exposure. Data represent the mean ± SE (standard error) of at least two experiments in which each treatment was tested in triplicate (*n* = 6).

Time Exposure	IC_50_ [µL/100 µL] (CL)			
	H69		BEAS-2B	
	**24 h**	**48 h**	**24 h**	**48 h**
MRSB	32.6 (29.0–36.7)	22.3 (14.7–34.0)	3.2 (2.0–5.1)	1.7 (1.2–2.3)
SurP	37.8 (32.2–44.4)	20.7 (15.7–27.3)	2.9 (1.8–4.5)	1.6 (1.2–2.2)
SurM 10K/MRSB	31.4 (27.5–35.9)	13.0 (10.5–16.4)	1.3 (0.5–3.3)	1.6 (1.2–2.2)
SurM 10K/RPMI	69.7 (46.6–104.1) *** ^§^	26.2 (16.7–36.8) ^§^	11.3 (8.5–15.5) *** ^§^	9.1 (7.5–11.0) *** ^§^
SurE 10K	32.8 (26.9–40.0)	20.7 (11.2–27.8)	3.0 (2.1–4.1)	1.5 (1.1–2.0)
SurE 3K	39.2 (32.5–47.4)	22.4 (17.5–28.7)	2.1 (1.6–2.7)	1.6 (1.2–2.1)

CL, confidential limits. *** *p* < 0.001 (ANOVA + multiple Dunnett’s comparison post-test), significant difference from the IC_50_ value of MRSB in the same time exposure. ^§^ *p* < 0.001 (*t*-Student test), significantly higher than SurM 10K/RPMI.

## Data Availability

The datasets generated and analyzed in the current study are available from the corresponding author on reasonable request.

## Supplementary Materials

The following are available online at https://www.mdpi.com/2076-2607/8/11/1653/s1, Figure S1: Representative ESI(+) FT-ICR mass spectra obtained from biofilm and planktonic vesicles extracts (bMVs and pMVs, respectively) and SurE 10K and SurM 10K supernatants; Figure S2. Representative ESI(-) FT-ICR mass spectra obtained for biofilm and planktonic vesicles extracts (bMVs and pMVs, respectively) and SurE 10K and SurM 10K supernatants; Figure S3. ESI(-) CID experiment conducted on peak m/z 299 allowed the identification of hydroxy stearic acid; Figure S4. Cytotoxic effect in human epithelial H69 cholangiocytes after 24 and 48 h exposure compared the control; Figure S5. Cytotoxic effect in human bronchial epithelial BEAS-2B cells after 24 and 48 h exposure compared the control; Figure S6. Time-schedules of the long-term exposures of 24 and 48 h; Figure S7. Histograms of the relative frequency of CH, CHN, CHNO, CHNOP, CHNOS, CHO, CHOP, and CHOS compounds; Figure S8. The number of common and uncommon features in pMVs and bMVs (upper panel) and SurE 10K and SurM 10K (lower panel) are summarized in Venn diagrams; Table S1: ESI FT-ICR MS comprehensive list of metabolites detected in bMVs extract; Table S2: ESI FT-ICR MS comprehensive list of metabolites detected in pMVs extract; Table S3: ESI FT-ICR MS comprehensive list of metabolites detected in SurE 10K; Table S4: ESI FT-ICR MS comprehensive list of metabolites detected in SurM 10K.
