# Supplementary material for: Correlation between the Antimicrobial Activity and Metabolic Profiles of Cell Free Supernatants and Membrane Vesicles Produced by Lactobacillus reuteri DSM 17938
Source: Microorganisms. 2020 Oct 24;8(11):1653. doi: 10.3390/microorganisms8111653 (PMC7692313; doi:10.3390/microorganisms8111653)
Supplement: Supplementary file 1 [file microorganisms-08-01653-s001.pdf]

Supplementary Material

p2 Figure S1. Representative ESI(+) FT-ICR mass spectra obtained from biofilm and planktonic vesicles extracts (bMVs and pMVs, respectively) and SurE 10K and SurM 10K supernatants.
p3 Figure S2. Representative ESI(-) FT-ICR mass spectra obtained for biofilm and planktonic vesicles extracts (bMVs and pMVs, respectively) and SurE 10K and SurM 10K supernatants.
p4 Figure S3. ESI(-) CID experiment conducted on peak *m*/*z* 299 allowed the identification of hydroxy stearic acid.
p5 Figure S4. Cytotoxic effect in human epithelial H69 cholangiocytes after 24 and 48 h exposure compared the control. MRSB, bacterial medium. RPMI, cell culture medium. SurP, total planktonic supernatant, pMVs included. SurM 10K, just vesicles (>10K). SurE 10K, fractionated planktonic supernatant (<10K). SurE 3K, fractionated planktonic supernatant (<3K). Data are expressed as mean ± SE (standard error) of at least two experiments in which each treatment was tested at least in triplicate (n = 6). Data are expressed as mean ± SE (standard error) of at least two experiments in which each treatment was tested at least in triplicate (n = 6). $^{*}p < 0.05$, $^{**}p < 0.01$, $^{***}p < 0.001$ (ANOVA + Multiple Dunnett's Comparison Post-test), significantly lower than the control after the same time exposure. $^{§}p < 0.001$ (t-Student test), significantly lower than 24 h.
p6 Figure S5. Cytotoxic effect in human bronchial epithelial BEAS-2B cells after 24 and 48 h exposure compared the control. MRSB, bacterial medium. RPMI, cell culture medium. SurP, total planktonic supernatant, pMVs included. SurM 10K, just vesicles (>10K). SurE 10K, fractionated planktonic supernatant (<10K). SurE 3K, fractionated planktonic supernatant (<3K). Data are expressed as mean ± SE (standard error) of at least two experiments in which each treatment was tested at least in triplicate (n = 6). $^{***}p < 0.001$ (ANOVA + Multiple Dunnett's Comparison Post-test), significantly lower than the control after the same time exposure. $^{§}p < 0.001$ (t-Student test), significantly lower than 24 h.
p7 Figure S6. Time-schedules of the long-term exposures of 24 and 48 h.
p8 Figure S7. Histograms of the relative frequency of CH, CHN, CHNO, CHNOP, CHNOS, CHO,CHOP, and CHOS compounds.
p9 Figure S8. The number of common and uncommon features in pMVs and bMVs (upper panel) and SurE 10K and SurM 10K (lower panel) are summarized in Venn diagrams.
p10 Table S1. ESI FT-ICR MS comprehensive list of metabolites detected in bMVs extract.
p19 Table S2. ESI FT-ICR MS comprehensive list of metabolites detected in pMVs extract.
p26 Table S3. ESI FT-ICR MS comprehensive list of metabolites detected in SurE 10K.
p40 Table S4. ESI FT-ICR MS comprehensive list of metabolites detected in SurM 10K.

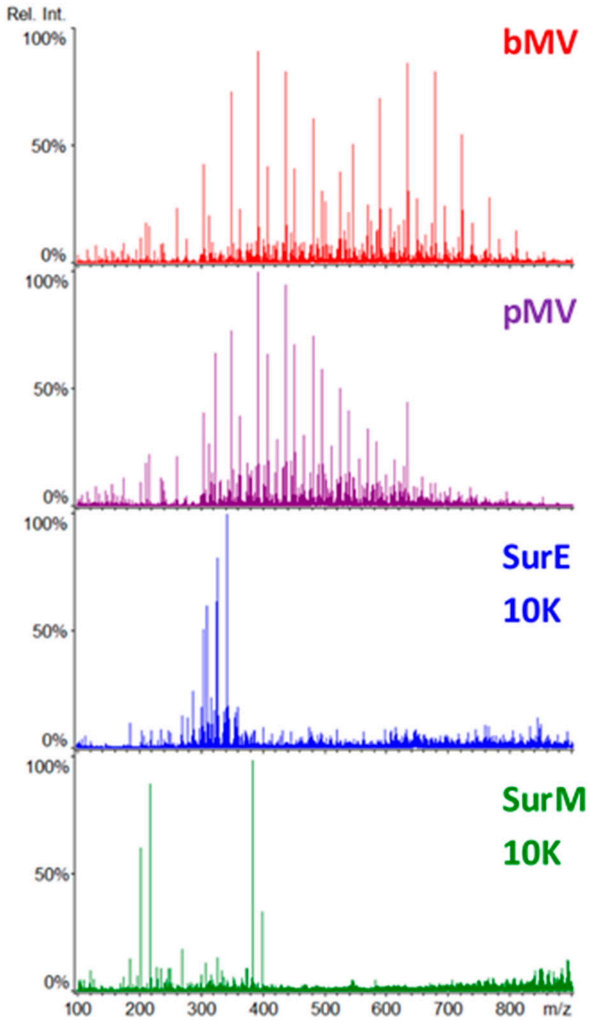

Figure S1. Representative ESI(+) FT-ICR mass spectra obtained for biofilm and planktonic vesicles extracts (bMVs and pMVs, respectively) and SurE 10K and SurM 10K supernatants.

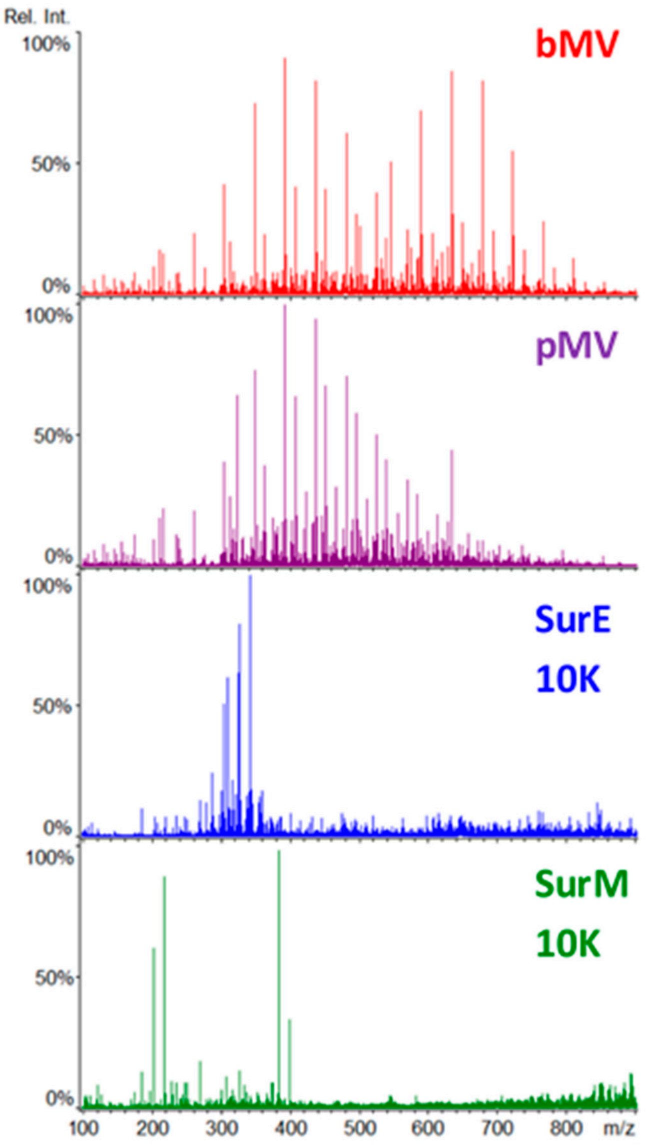

Figure S2. Representative ESI(-) FT-ICR mass spectra obtained for biofilm and planktonic vesicles extracts (bMVs and pMVs, respectively) and SurE 10K and SurM 10K supernatants.

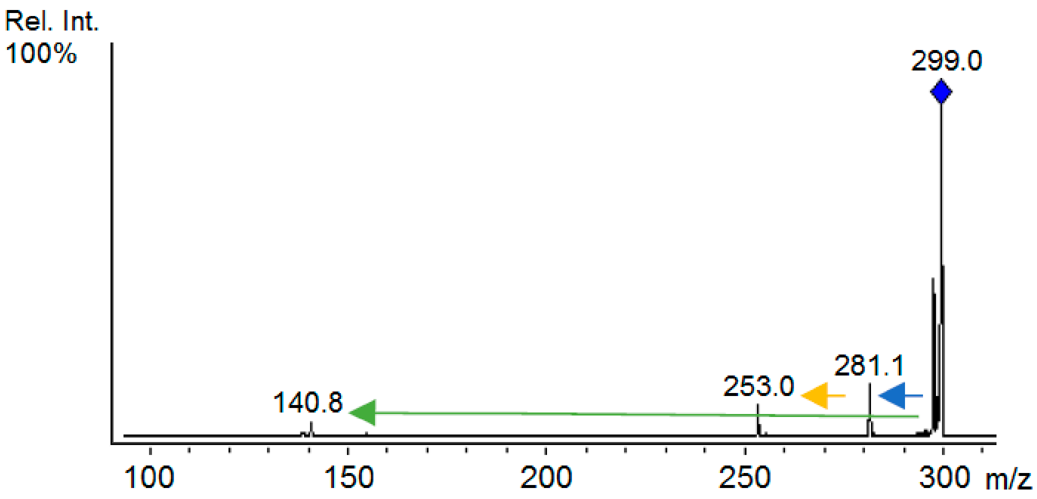

Figure S3. ESI(-) CID experiment conducted on peak *m*/*z* 299 allowed the identification of hydroxy stearic acid.

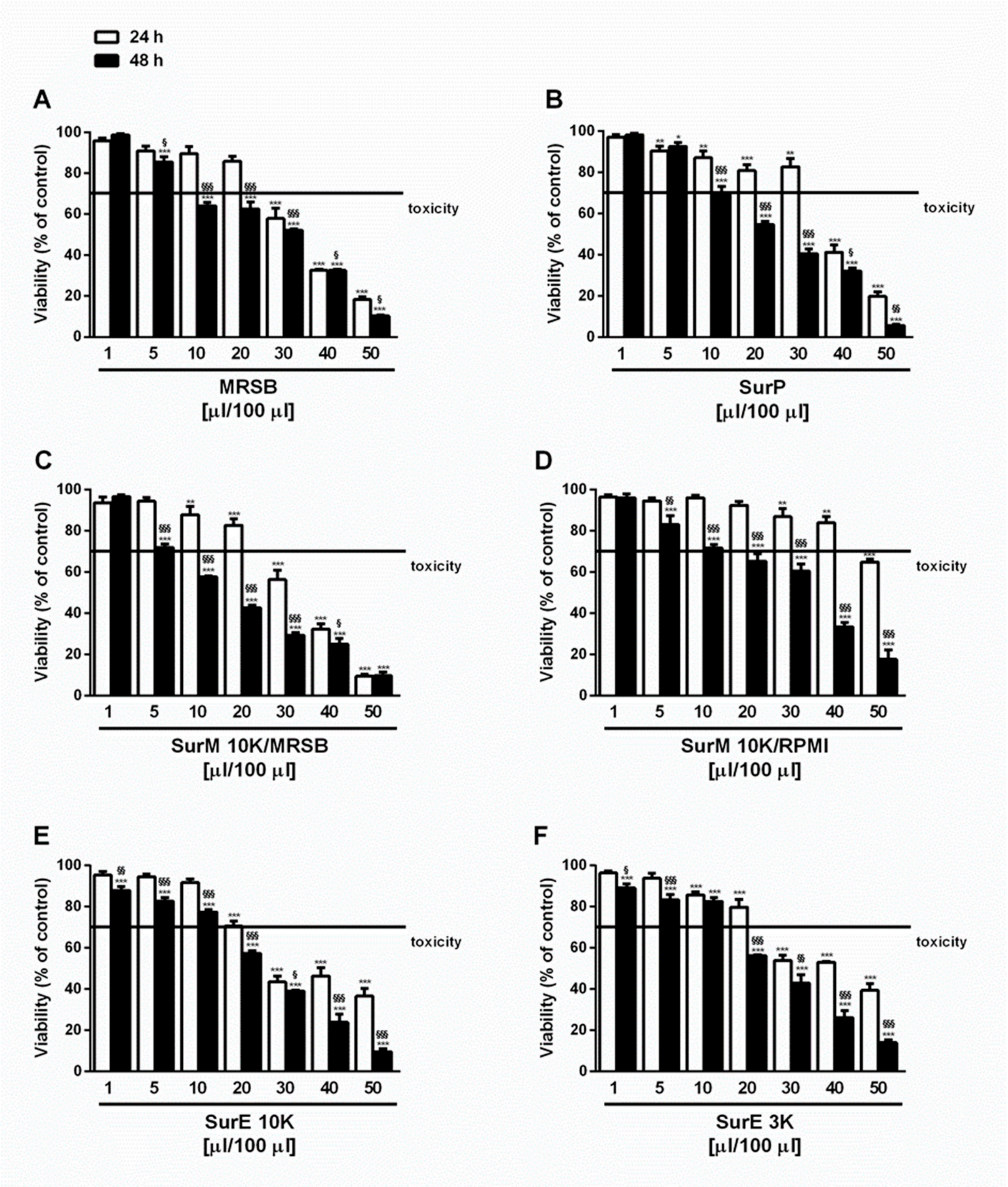

Figure S4. Cytotoxic effect in human epithelial H69 cholangiocytes after 24 and 48 h exposure compared the control. MRSB, bacterial medium. RPMI, cell culture medium. SurP, total planktonic supernatant, pMVs included. SurM 10K, just vesicles (>10K). SurE 10K, fractionated planktonic supernatant (<10K). SurE 3K, fractionated planktonic supernatant (<3K). Data are expressed as mean ± SE (standard error) of at least two experiments in which each treatment was tested at least in triplicate (n = 6). Data are expressed as mean ± SE (standard error) of at least two experiments in which each treatment was tested at least in triplicate (n = 6). $^{*}p < 0.05$, $^{**}p < 0.01$, $^{***}p < 0.001$ (ANOVA + Multiple Dunnett's Comparison Post-test), significantly lower than the control after the same time exposure. $^{\S}p < 0.001$ (t-Student test), significantly lower than 24 h.

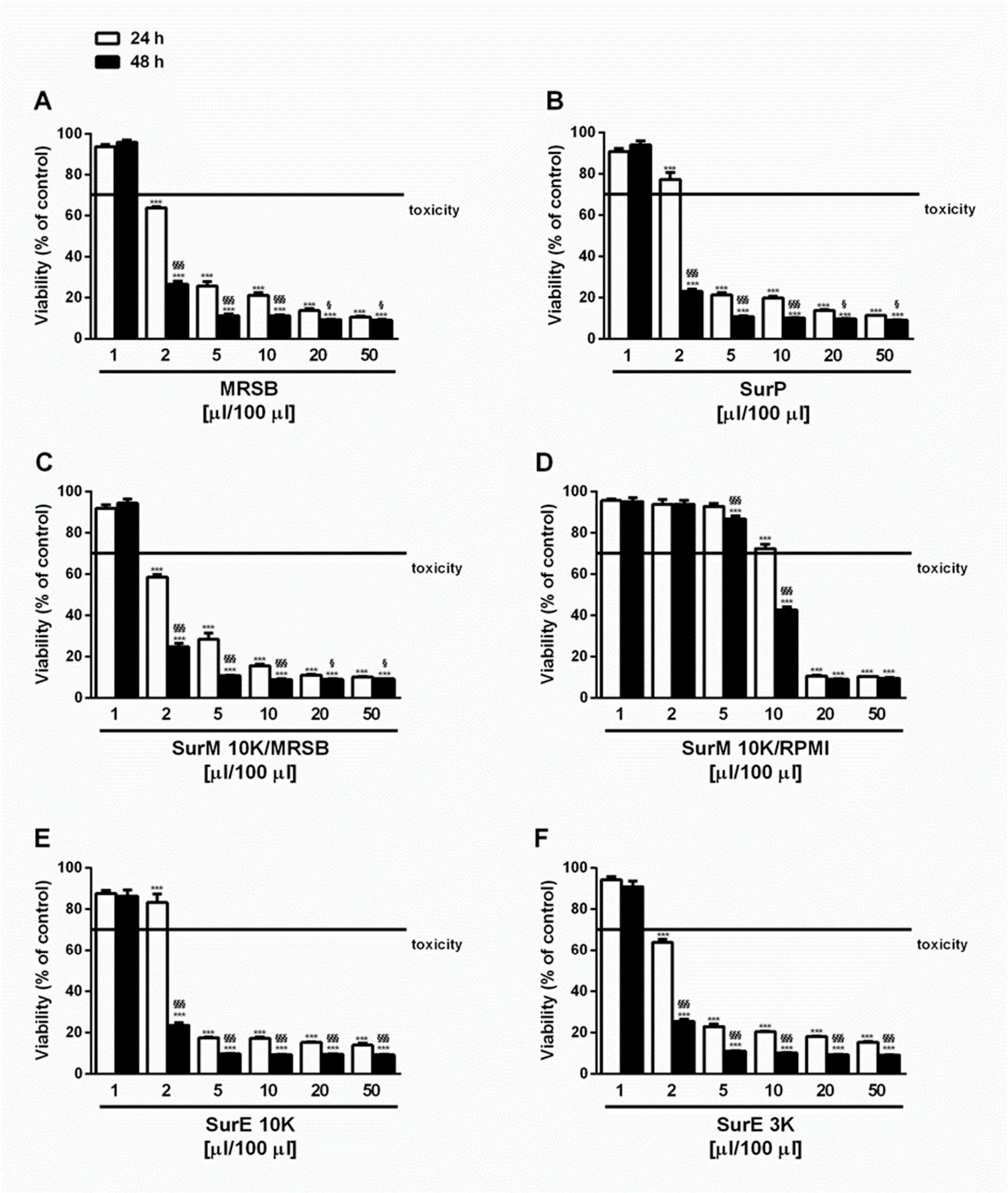

Figure S5. Cytotoxic effect in human bronchial epithelial BEAS-2B cells after 24 and 48 h exposure compared the control. MRSB, bacterial medium. RPMI, cell culture medium. SurP, total planktonic supernatant, pMVs included. SurM 10K, just vesicles (>10K). SurE 10K, fractionated planktonic supernatant (<10K). SurE 3K, fractionated planktonic supernatant (<3K). Data are expressed as mean ± SE (standard error) of at least two experiments in which each treatment was tested at least in triplicate (n = 6). $^{***}p < 0.001$ (ANOVA + Multiple Dunnett's Comparison Post-test), significantly lower than the control after the same time exposure. $^{\S}p < 0.001$ (t-Student test), significantly lower than 24 h.

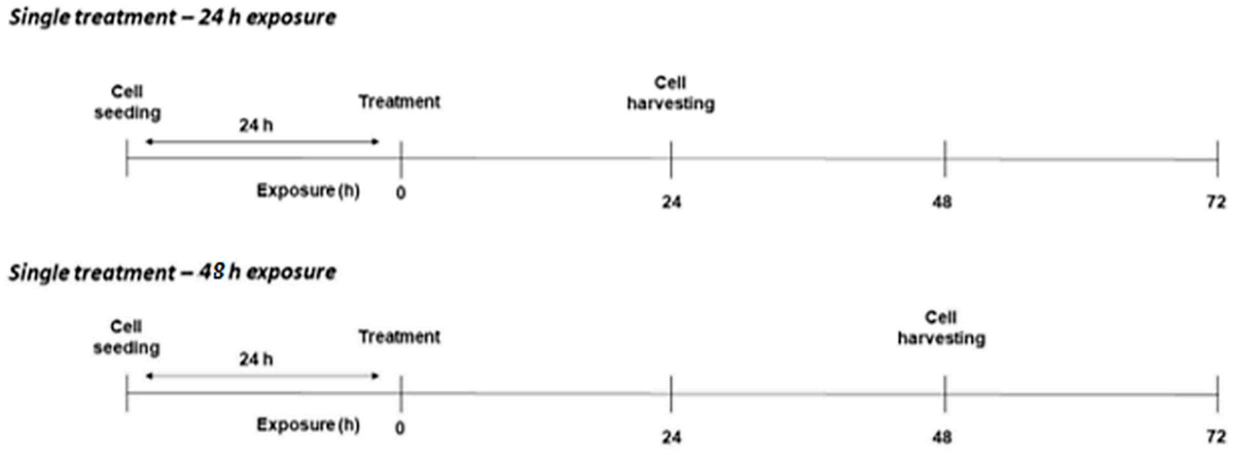

Figure S6. Time-schedules of the long-term exposures of 24 and 48 h.

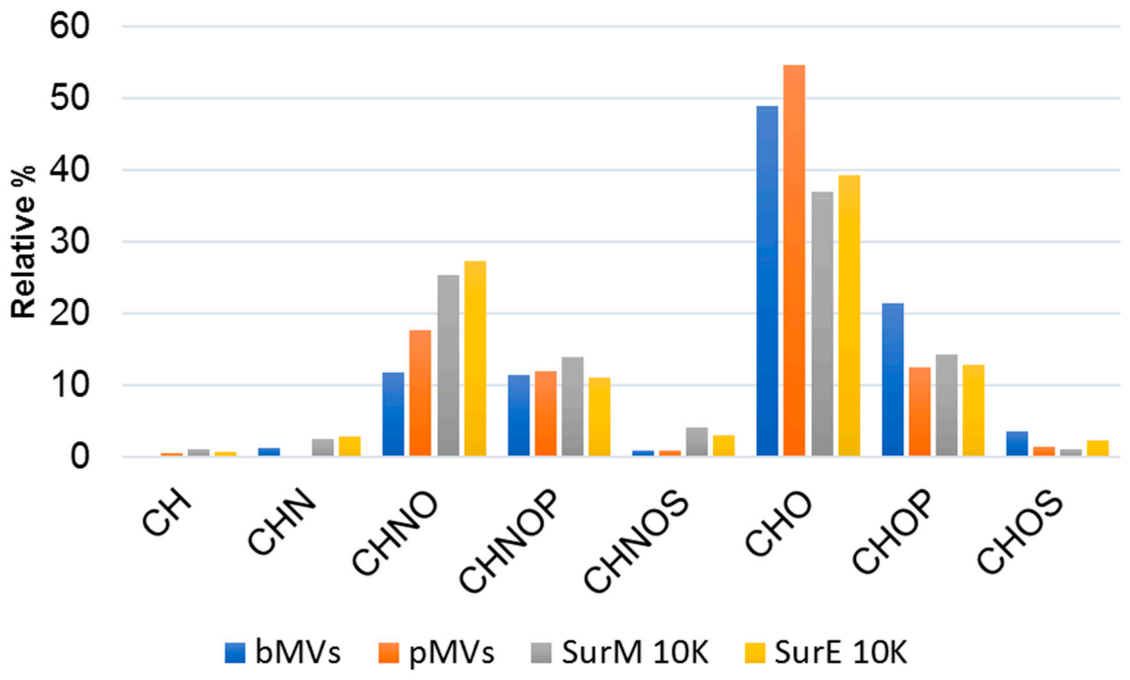

Figure S7. Histograms of the relative frequency of CH, CHN, CHNO, CHNOP, CHNOS, CHO, CHOP, and CHOS compounds.

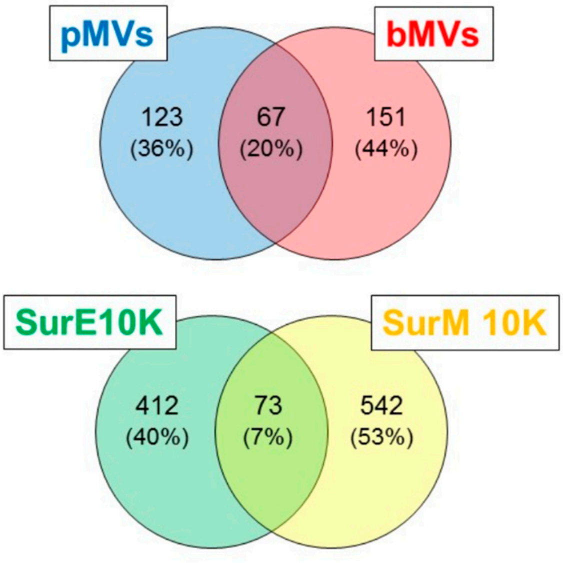

Figure S8. The number of common and uncommon features in pMVs and bMVs (upper panel) and SurE 10K and SurM 10K (lower panel) are summarized in Venn diagrams.

Table S2. ESI FT-ICR MS comprehensive list of metabolites detected in bMVs extract.

#	Putative Annotation (M)[a]	Ion	Formula	Theor. *m/z*[b]	Exp. *m/z*[c]	ppm[d]	Peak_Height
1	Lactic acid	[M-H]-	C3H6O3	89.02442	89.02438	-0.4	2.3E+06
2	Acetyl-imidazole	[M+Cl]-	C5H6N2O	145.01741	145.01746	0.3	2.3E+06
3	Adipic acid	[M-H]-	C6H10O4	145.05063	145.05071	0.5	3.4E+05
4	Glutamic acid	[M-H]-	C5H9NO4	146.04588	146.04589	0.1	9.3E+06
5	Diethylphosphate	[M-H]-	C4H11O4P	153.03222	153.03226	0.3	7.3E+05
6	3-hydroxy valeric acid	[M+Cl]-	C5H10O3	153.03240	153.03226	-0.9	7.3E+05
7	Nonanoic acid	[M-H]-	C9H18O2	157.12340	157.12320	-1.3	4.5E+05
8	D-Glutamate	[M+Na]+	C5H9NO4	170.04238	170.04189	-2.9	7.2E+05
9	9-Oxononanoic acid	[M-H]-	C9H16O3	171.10267	171.10276	0.5	3.1E+05
10	Triethanolamine	[M+Na]+	C6H15NO3	172.09441	172.09398	-2.5	6.9E+05
11	Arginine	[M+H]+	C6H14N4O2	175.11895	175.11893	-0.1	1.8E+06
12	Aldohexose	[M-H]-	C6H12O6	179.05611	179.05605	-0.3	7.3E+05
13	Dicyclohexylamine	[M+H]+	C12H23N	182.19033	182.18998	-1.9	1.0E+06
14	Oxodecenoic acid	[M-H]-	C10H16O3	183.10267	183.10267	0.0	5.3E+05
15	Azelaic acid	[M-H]-	C9H16O4	187.09758	187.09752	-0.3	1.0E+06
16	Quinic acid	[M-H]-	C7H12O6	191.05611	191.05615	0.2	6.2E+05
17	L-Tyrosine methyl ester	[M-H]-	C10H13NO3	194.08227	194.08239	0.6	3.4E+05
18	D-Arginine	[M+Na]+	C6H14N4O2	197.10090	197.10036	-2.7	5.4E+05
19	Dodecanoic acid	[M-H]-	C12H24O2	199.17035	199.17069	1.7	1.6E+06
20	11-amino-undecanoic acid	[M+H]+	C11H23NO2	202.18016	202.17962	-2.6	8.1E+05
21	Aldohexose	[M+Na]+	C6H12O6	203.05261	203.05234	-1.3	1.7E+07
22	D-Sorbitol	[M+Na]+	C6H14O6	205.06826	205.06846	1.0	5.2E+05
23	5-oxo-7E-decenoic acid	[M+Na]+	C10H16O3	207.09917	207.09865	-2.5	8.7E+05
24	cis-2-Carboxycyclohexyl-acetic acid	[M+Na]+	C9H14O4	209.07843	209.07814	-1.4	5.5E+05
25	2-hydroxy pelargonic acid	[M+Cl]-	C9H18O3	209.09500	209.09553	2.6	8.5E+05
26	D-Glucose	[M+Cl]-	C6H12O6	215.03279	215.03297	0.8	5.0E+06
27	Aldohexose	[M+K]+	C6H12O6	219.02655	219.02676	1.0	6.6E+05
28	4-*n*-Nonylphenol	[M-H]-	C15H24O	219.17544	219.17582	1.7	6.5E+05
29	2E-Decenedioic acid	[M+Na]+	C10H16O4	223.09408	223.09403	-0.2	7.7E+05

#	Putative Annotation (M)[a]	Ion	Formula	Theor. *m/z*[b]	Exp. *m/z*[c]	ppm[d]	Peak_Height
30	5-Acetylamino-6-formylamino-3-methyluracil	[M-H]-	C8H10N4O4	225.06293	225.06253	-1.8	5.0E+06
31	10-hydroxy-undecanoic acid	[M+Na]+	C11H22O3	225.14612	225.14556	-2.5	7.3E+05
32	4-(Dimethylamino)azobenzene	[M+H]+	C14H15N3	226.13387	226.13362	-1.1	1.4E+06
33	Myristic acid	[M-H]-	C14H28O2	227.20165	227.20175	0.4	8.5E+05
34	Sinapyl alcohol	[M+Na]+	C11H14O4	233.07843	233.07853	0.4	6.6E+05
35	Guanethidine	[M+Cl]-	C10H22N4	233.15385	233.15419	1.5	6.4E+05
36	alpha-Cyperone	[M-H]-	C15H22O2	233.15470	233.15419	-2.2	6.4E+05
37	10-hydroxy-undecanoic acid	[M+Cl]-	C11H22O3	237.12630	237.12649	0.8	4.5E+05
38	10-hydroxy-11-dodecenoic acid	[M+Na]+	C12H22O3	237.14612	237.14673	2.6	5.5E+05
39	(*R*)-3-Hydroxydodecanoic acid	[M+Na]+	C12H24O3	239.16177	239.16196	0.8	4.6E+05
40	Pentadecanoic acid	[M-H]-	C15H30O2	241.21730	241.21742	0.5	7.3E+05
41	Apiole	[M+Na]+	C12H14O4	245.07843	245.07781	-2.5	8.3E+05
42	Myristoleic acid	[M+Na]+	C14H26O2	249.18250	249.18204	-1.8	5.4E+05
43	Myristic acid	[M+Na]+	C14H28O2	251.19815	251.19832	0.7	1.3E+06
44	Palmitoleic acid	[M-H]-	C16H30O2	253.21730	255.21768	1.4	9.3E+05
45	Encecalin	[M+Na]+	C14H16O3	255.09917	255.09903	-0.5	1.8E+06
46	Palmitic acid	[M-H]-	C16H32O2	255.23295	267.23295	0.2	5.9E+06
47	Macrophyllic acid A	[M+Na]+	C15H22O2	257.15120	257.15088	-1.2	6.1E+05
48	3-Dimethylallyl-4-hydroxymandelic acid	[M+Na]+	C13H16O4	259.09408	259.09364	-1.7	5.6E+05
49	(+)-12-methyl myristic acid	[M+Na]+	C15H30O2	265.21380	265.21384	0.1	1.2E+06
50	Heptadecenoic acid	[M-H]-	C17H32O2	267.23295	269.08903	0.0	1.8E+06
51	6-Hydroxyl-1,6-dihydropurine ribonucleoside	[M-H]-	C10H14N4O5	269.08914	269.24885	-0.4	1.8E+06
52	Methyl palmitate	[M-H]-	C17H34O2	269.24860	279.23338	0.9	5.7E+05
53	3,6,4'-Trihydroxyflavone	[M+H]+	C15H10O5	271.06010	271.05932	-2.9	5.6E+05
54	Podocarpic acid	[M+H]+	C17H22O3	275.16417	275.16342	-2.7	1.4E+06
55	Glu-Glu	[M+H]+	C10H16N2O7	277.10303	277.10259	-1.6	2.2E+06
56	Palmitoleic acid	[M+Na]+	C16H30O2	277.21380	277.21420	1.4	1.5E+06
57	Palmitic acid	[M+Na]+	C16H32O2	279.22945	279.22972	1.0	1.5E+06
58	Linoleic Acid	[M-H]-	C18H32O	279.23295	281.24865	1.5	3.8E+06
59	Oleic acid	[M-H]-	C18H34O2	281.24860	283.26466	0.2	7.0E+06

#	Putative Annotation (M)[a]	Ion	Formula	Theor. *m/z*[b]	Exp. *m/z*[c]	ppm[d]	Peak_Height
60	Stearic acid	[M-H]-	C18H36O2	283.26425	287.22309	1.4	6.6E+06
61	Dihydroxy-palmitic acid	[M-H]-	C16H32O4	287.22278	291.21045	1.1	7.3E+05
62	C17 Sphinganine	[M+H]+	C17H37NO2	288.28971	288.28902	-2.4	7.0E+05
63	(1*R*,2*R*)-3-oxo-2-pentyl-cyclopentanehexanoic acid	[M+Na]+	C16H28O3	291.19307	291.19286	-0.7	6.4E+05
64	Palmitic acid	[M+Cl]-	C16H32O2	291.20963	293.21226	2.8	7.8E+05
65	4'-Hydroxy-7-methoxy-8-methylflavan	[M+Na]+	C17H18O3	293.11482	293.11478	-0.1	1.2E+06
66	17-Hydroxylinolenic acid	[M-H]-	C18H30O3	293.21222	295.22818	0.1	5.1E+05
67	12*R*-hydroxy-9*Z*,15*Z*-octadecadienoic acid	[M-H]-	C18H32O3	295.22787	298.15718	1.1	1.5E+06
68	Annofoline	[M+Cl]-	C16H25NO2	298.15793	299.25950	-2.5	7.7E+05
69	(*R*)-2-Hydroxystearate	[M-H]-	C18H36O3	299.25917	301.23844	1.1	5.9E+06
70	Alpha-Linolenic acid	[M+Na]+	C18H30O2	301.21380	301.21439	2.0	7.3E+05
71	MG(0:0/14:0/0:0)	[M-H]-	C17H34O4	301.23843	312.17227	0.0	3.9E+06
72	Phaseic acid	[M+Na]+	C15H20O5	303.12029	303.11964	-2.2	1.7E+06
73	Linoleic acid	[M+Na]+	C18H32O2	303.22945	303.22966	0.7	5.1E+06
74	4-Oxo-13-*cis*-retinoate	[M-H]-	C20H25O3	312.17309	315.20907	-2.6	3.6E+06
75	Linoleic acid	[M+Cl]-	C18H32O2	315.20963	327.25485	-1.8	5.7E+05
76	12*R*-hydroxy-9*E*-octadecenoic acid	[M+Na]+	C18H34O3	321.24002	321.24011	0.3	1.1E+06
77	5,7,4'-Trimethoxyflavan	[M+Na]+	C18H20O4	323.12538	323.12504	-1.1	7.1E+05
78	(*R*)-10-hydroxystearic acid	[M+Na]+	C18H36O3	323.25567	323.25537	-0.9	6.8E+06
79	Tributyrin	[M+Na]+	C15H26O6	325.16216	325.16228	0.4	2.3E+06
80	4,7,10,13-Docosatetraynoic acid	[M+H]+	C22H28O2	325.21621	325.21539	-2.5	7.8E+05
81	MG(0:0/16:1(9Z)/0:0)	[M-H]-	C19H36O4	327.25408	329.27050	2.3	2.4E+06
82	10-nitro-9*E*-octadecenoic acid	[M+H]+	C18H33NO4	328.24824	328.24883	1.8	1.0E+06
83	(*Z*)-11beta,21-Dihydroxypregna-1,4,17(20)-trien-3-one	[M+H]+	C21H28O3	329.21112	329.21053	-1.8	1.3E+06
84	MG(0:0/16:0/0:0)	[M-H]-	C19H38O4	329.26973	340.20549	2.3	2.8E+06
85	Lipoyllysine	[M+H]+	C14H26N2O3S2	335.14576	335.14676	3.0	1.0E+06
86	(9*Z*)-(7*S*,8*S*)-Dihydroxyoctadecenoic acid	[M+Na]+	C18H34O4	337.23493	337.23497	0.1	4.6E+06
87	17beta-Nitro-5alpha-androstane	[M+Cl]-	C19H31NO2	340.20488	353.08776	1.8	2.7E+06
88	2-hydroxy-eicosanoic acid	[M+Na]+	C20H40O3	351.28697	351.28790	2.7	8.1E+05
89	Chlorogenic acid	[M-H]-	C16H18O9	353.08781	367.25003	-0.1	9.0E+06

#	Putative Annotation (M)[a]	Ion	Formula	Theor. *m/z*[b]	Exp. *m/z*[c]	ppm[d]	Peak_Height
90	MG(0:0/16:0/0:0)	[M+Na]+	C19H38O4	353.26623	353.26651	0.8	1.4E+07
91	18-hydroxy-9*S*,10*R*-dihydroxy-stearic acid	[M+Na]+	C18H36O5	355.24549	355.24492	-1.6	1.4E+06
92	MG(0:0/18:2(9Z,12Z)/0:0)	[M+H]+	C21H38O4	355.28429	355.28323	-3.0	9.8E+05
93	2-alpha-D-Glucosyl-D-glucose	[M+Na]+	C12H22O11	365.10543	365.10434	-3.0	4.4E+06
94	16,17-epoxy-DHA	[M+Na]+	C22H30O3	365.20872	365.20815	-1.5	1.3E+06
95	Cortol	[M-H]-	C21H36O5	367.24900	387.11735	2.8	6.6E+05
96	(3*S*,4*S*)-3-Hydroxytetradecane-1,3,4-tricarboxylate	[M+Na]+	C17H30O7	369.18837	369.18805	-0.9	3.4E+06
97	(20*S*)-3beta-Hydroxychola-5,16-dien-24-oic Acid	[M+H]+	C24H36O3	373.27372	373.27317	-1.5	1.5E+06
98	6,7-dihydro-5-oxo-12-epi-LTB4	[M+K]+	C20H31O4	374.18539	374.18569	0.8	1.5E+06
99	Trimethylolpropane trimethacrylate	[M+K]+	C18H26O6	377.13610	377.13715	2.8	1.3E+06
100	1alpha,5alpha-Dimercaptoandrostane-3alpha,17beta-diol	[M+Na]+	C19H32O2S2	379.17359	379.17330	-0.8	7.5E+06
101	2,3-Dinor-6-keto-prostaglandin F1 a	[M+K]+	C18H30O6	381.16740	381.16738	0.0	9.1E+05
102	MG(0:0/18:0/0:0)	[M+Na]+	C21H42O4	381.29753	381.29757	0.1	1.4E+07
103	Fructoselysine 6-phosphate	[M-H]-	C12H25N2O10P	387.11741	393.27707	-0.1	6.3E+05
104	Fulvinervin B	[M+H]+	C25H22O4	387.15909	387.15924	0.4	9.3E+05
105	Rehmaionoside A	[M+H]+	C19H34O8	391.23264	391.23177	-2.2	3.5E+06
106	MG(18:0/0:0/0:0)	[M+Cl]-	C21H42O4	393.27771	546.13727	-1.6	1.7E+06
107	9'-carboxy-gamma-tocotrienol	[M+Na]+	C23H31O4	394.21146	394.21252	2.7	2.4E+07
108	2,4,2'-Trihydroxy-6",6"-dimethyl-3'-prenylpyrano[2",3":4',5']chalcone	[M+H]+	C25H26O5	407.18530	407.18515	-0.4	1.4E+07
109	9'-Carboxy-alpha-tocotrienol	[M+Na]+	C24H33O4	408.22711	408.22807	2.4	1.2E+07
110	9'-carboxy-gamma-tocotrienol	[M+K]+	C23H31O4	410.18539	410.18578	0.9	2.8E+06
111	Prebarbigerone	[M+H]+	C24H26O6	411.18022	411.18029	0.2	1.4E+06
112	Rehmaionoside B	[M+Na]+	C19H34O8	413.21459	413.21427	-0.8	5.5E+06
113	Diisooctyl phthalate	[M+Na]+	C24H38O4	413.26623	413.26721	2.4	2.9E+06
114	9'-Carboxy-gamma-chromanol	[M+K]+	C23H35O4	414.21669	414.21674	0.1	1.0E+06
115	Distigmine	[M+H]+	C22H32N4O4	417.24963	417.24879	-2.0	1.9E+06
116	Sphingosine 1-phosphate	[M+K]+	C18H38NO5P	418.21192	418.21243	1.2	2.2E+06
117	Gangetinin	[M+H]+	C26H26O5	419.18530	419.18457	-1.7	1.2E+06
118	(10E)-19-methylvitamin D3	[M+Na]+	C28H46O	421.34409	421.34337	-1.7	1.1E+06
119	9'-Carboxy-alpha-tocotrienol	[M+K]+	C24H33O4	424.20104	424.20191	2.0	3.4E+06

#	Putative Annotation (M)[a]	Ion	Formula	Theor. *m/z*[b]	Exp. *m/z*[c]	ppm[d]	Peak_Height
120	(22E)-3alpha,6beta,7beta-Trihydroxy-5beta-chol-22-en-24-oic Acid	[M+K]+	C24H38O5	445.23508	445.23641	3.0	4.1E+06
121	3',4',6'-Trihydroxy-2'-oxo-3',5'-diprenyldihydrochalcone	[M+K]+	C25H30O5	449.17248	449.17321	1.6	1.6E+06
122	LysoPC(10:0)	[M+K]+	C18H39NO7P	451.20957	451.21000	0.9	1.4E+07
123	17-phenyl-trinor-PGF2alpha isopropyl ester	[M+Na]+	C26H38O5	453.26115	453.26088	-0.6	3.5E+06
124	1alpha-hydroxy-24-methylsulfonyl-25,26,27-trinorvitamin D3	[M+Na]+	C25H40O4S	459.25395	459.25444	1.1	1.4E+06
125	Glutathionylspermidine	[M+K]+	C17H34N6O5S	473.19430	473.19553	2.6	2.9E+06
126	C17 sphingosine-1-phosphocholine	[M+Na]+	C22H47N2O5P	473.31148	473.31018	-2.7	1.3E+06
127	Gentamicin X2	[M+H]+	C19H38N4O10	483.26607	483.26678	1.5	4.6E+06
128	cholesterol sulfate	[M+Na]+	C27H46O4S	489.30090	489.30017	-1.5	3.6E+06
129	Gentamicin C1	[M+Na]+	C21H43N5O7	500.30547	500.30430	-2.3	2.1E+06
130	3-Geranyl-4,2',4',6'-tetrahydroxy-5-prenyldihydrochalcone	[M+Na]+	C30H38O5	501.26115	501.26258	2.9	1.4E+07
131	(6R)-1alpha,25-dihydroxyvitamin D3 6,19-sulfur dioxide adduct	[M+Na]+	C27H44O5S	503.28017	503.27936	-1.6	4.7E+06
132	N-arachidonoyl tyrosine	[M+K]+	C29H41NO4	506.26672	506.26562	-2.2	3.2E+06
133	29-demethylgeodisterol-O-sulfite	[M+Na]+	C27H40O6S	515.24378	515.24243	-2.6	1.7E+06
134	27-nor-campestan-nonol	[M+H]+	C27H48O9	517.33711	517.33569	-2.7	2.7E+06
135	Mycalamide B	[M+H]+	C25H43NO10	518.29597	518.29484	-2.2	2.2E+06
136	LysoPE(0:0/20:3(5Z,8Z,11Z))	[M+Na]+	C25H46NO7P	526.29041	526.28952	-1.7	1.6E+07
137	geodisterol-O-sulfite	[M+Na]+	C28H42O6S	529.25943	529.26016	1.4	2.2E+06
138	PG(20:4(5Z,8Z,11Z,14Z)/0:0)	[M+H]+	C26H45O9P	533.28740	533.28790	0.9	4.8E+06
139	11-(3-acetoxy-1-propynyl)-1alpha,25-dihydroxy-9,11-didehydrovitamin D3	[M+Na]+	C32H46O5	533.32375	533.32498	2.3	4.3E+06
140	Dihydromethanophenazine	[M+H]+	C37H52N2O	541.41524	541.41468	-1.0	3.2E+06
141	2-deoxy-20-hydroxyecdysone 22-phosphate	[M+H]+	C27H45O9P	545.28740	545.28751	0.2	2.0E+07
142	Hygromycin A	[M+Cl]-	C23H29NO12	546.13838	546.25951	-2.0	1.1E+06
143	PS(17:0/0:0)	[M+Cl]-	C23H46NO9P	546.26042		-1.7	4.9E+05
144	Trihydroxy-27-carboxymethyl-5beta-cholestan-26-oic acid	[M+K]+	C29H48O7	547.30316	547.30454	2.5	6.2E+06
145	DG(14:1(9Z)/14:1(9Z)/0:0)	[M+K]+	C31H56O5	547.37593	547.37517	-1.4	6.1E+06
146	(+)-Myristinin A	[M+H]+	C33H40O7	549.28468	549.28590	2.2	9.6E+06
147	PS(O-18:0/0:0)	[M+K]+	C24H50NO8P	550.29056	550.28924	-2.4	2.7E+06
148	Enkephalin L	[M+H]+	C28H37N5O7	556.27658	556.27758	1.8	3.1E+06
149	PG(10:0/10:0)	[M+Na]+	C26H51O10P	577.31121	577.31234	2.0	2.7E+06

#	Putative Annotation (M)[a]	Ion	Formula	Theor. *m/z*[b]	Exp. *m/z*[c]	ppm[d]	Peak_Height
150	Prednisolone 21-all-*cis*-farnesylate	[M+H]+	C36H50O6	579.36802	579.36847	0.8	6.3E+06
151	2-deoxy-20-hydroxyecdysone 22-phosphate	[M+K]+	C27H45O9P	583.24328	583.24263	-1.1	5.1E+06
152	5-Oxoavermectin "1a" aglycone	[M+H]+	C34H46O8	583.32654	583.32731	1.3	2.1E+07
153	3-O-acetylecdysone 2-phosphate	[M+H]+	C29H47O10P	587.29796	587.29968	2.9	2.6E+06
154	PC(O-20:0/O-1:0)	[M+K]+	C29H62NO6P	590.39463	590.39401	-1.1	3.4E+07
155	Triterpenoid	[M+K]+	C30H48O7S	591.27523	591.27404	-2.0	4.1E+06
156	PA(12:0/16:1(9Z))	[M+H]+	C31H59O8P	591.40203	591.40140	-1.1	9.3E+06
157	Avermectin B1b aglycone	[M+K]+	C33H46O8	609.28243	609.28175	-1.1	6.7E+06
158	PI(20:4(5Z,8Z,11Z,14Z)/0:0)	[M+H]+	C29H49O12P	621.30344	621.30412	1.1	2.9E+06
159	25-Hydroxyvitamin D2 25-(beta-glucuronide)	[M+K]+	C34H52O8	627.32938	627.32987	0.8	1.0E+07
160	DG(18:4(6Z,9Z,12Z,15Z)/18:4(6Z,9Z,12Z,15Z)/0:0)	[M+Na]+	C39H60O5	631.43330	631.43177	-2.4	2.8E+06
161	4-Keto-4'-hydroxyalloxanthin	[M+K]+	C40H50O4	633.33407	633.33318	-1.4	2.0E+07
162	PI(19:0/0:0)	[M+Na]+	C28H55O12P	637.33233	637.33374	2.2	4.2E+06
163	Avermectin B2a aglycone	[M+K]+	C34H50O9	641.30864	641.30857	-0.1	4.8E+06
164	PI(22:6(4Z,7Z,10Z,13Z,16Z,19Z)/0:0)	[M+H]+	C31H49O12P	645.30344	645.30476	2.0	2.1E+06
165	6-Hydroxykaempferol 3,6-diglucoside	[M+Na]+	C27H30O17	649.13752	649.13846	1.4	4.2E+06
166	6-O-(Glcb)-(25R)-5alpha-spirostan-3beta,6alpha,23S-triol	[M+Na]+	C34H58O10	649.39222	649.39101	-1.9	5.4E+07
167	PG(14:1(9Z)/14:1(9Z))	[M+H]+	C34H63O10P	663.42316	663.42225	-1.4	1.8E+07
168	PG(12:0/16:1(9Z))	[M+H]+	C34H65O10P	665.43881	665.43831	-0.8	5.2E+06
169	DG(15:0/22:4(7Z,10Z,13Z,16Z)/0:0)	[M+K]+	C40H70O5	669.48548	669.48727	2.7	2.7E+06
170	Oleanoic acid 3-O-glucuronide	[M+K]+	C36H56O9	671.35559	671.35386	-2.6	1.1E+07
171	PI(22:2(13Z,16Z)/0:0)	[M+Na]+	C31H57O12P	675.34798	675.34806	0.1	3.0E+06
172	PG(O-16:0/12:0)	[M+Na]+	C34H69O9P	675.45714	675.45800	1.3	2.4E+06
173	PA(12:0/20:5(5Z,8Z,11Z,14Z,17Z))	[M+K]+	C35H59O8P	677.35791	677.35665	-1.9	1.8E+07
174	PG(12:0/17:2(9Z,12Z))	[M+H]+	C35H65O10P	677.43881	677.43757	-1.8	1.2E+08
175	PS(P-16:0/12:0)	[M+Na]+	C34H66NO9P	686.43674	686.43504	-2.5	3.3E+06
176	PI(22:2(13Z,16Z)/0:0)	[M+K]+	C31H57O12P	691.32192	691.32373	2.6	2.4E+06
177	PI(22:1(11Z)/0:0)	[M+K]+	C31H59O12P	693.33757	693.33659	-1.4	1.3E+07
178	PC(O-16:0/12:0)	[M+K]+	C36H74NO7P	702.48345	702.48509	2.3	2.4E+06
179	Pepstatin	[M+Na]+	C34H63N5O9	708.45180	708.45113	-0.9	3.9E+06

#	Putative Annotation (M)[a]	Ion	Formula	Theor. *m/z*[b]	Exp. *m/z*[c]	ppm[d]	Peak_Height
180	DG(20:3(5Z,8Z,11Z)/22:6(4Z,7Z,10Z,13Z,16Z,19Z)/0:0)	[M+Na]+	C45H70O5	713.51155	713.50991	-2.3	5.8E+06
181	PG(12:0/20:2(11Z,14Z))	[M+H]+	C38H71O10P	719.48576	719.48549	-0.4	2.0E+06
182	PG(O-16:0/16:1(9Z))	[M+Na]+	C38H75O9P	729.50409	729.50403	-0.1	3.9E+06
183	PC(O-14:0/16:0)	[M+K]+	C38H78NO7P	730.51475	730.51403	-1.0	2.2E+06
184	PG(13:0/20:1(11Z))	[M+H]+	C39H75O10P	735.51706	735.51801	1.3	5.1E+06
185	PC(14:1(9Z)/P-18:1(11Z))	[M+Na]+	C40H76NO7P	736.52516	736.52712	2.7	1.8E+06
186	PG(12:0/20:4(5Z,8Z,11Z,14Z))	[M+Na]+	C38H67O10P	737.43641	737.43759	1.6	3.5E+07
187	PS(13:0/18:2(9Z,12Z))	[M+Na]+	C37H68NO10P	740.44730	740.44578	-2.1	2.2E+06
188	1-(ladderane-hexanoyl)-2-(ladderane-octanyl)-sn-glycerophosphoethanolamine	[M+H]+	C43H72NO7P	746.51192	746.51190	0.0	2.2E+06
189	PS(P-16:0/17:2(9Z,12Z))	[M+Na]+	C39H72NO9P	752.48369	752.48144	-3.0	1.9E+06
190	Delavaine A	[M+K]+	C38H54N2O11	753.33592	753.33529	-0.8	1.9E+06
191	PS(14:0/20:5(5Z,8Z,11Z,14Z,17Z))	[M+H]+	C40H68NO10P	754.46536	754.46421	-1.5	1.8E+06
192	Spinoside A	[M+K]+	C39H56O12	755.34034	755.34193	2.1	1.8E+06
193	2,3-Bis-O-(geranylgeranyl)glycerol 1-phosphate	[M+K]+	C43H73O6P	755.47764	755.47793	0.4	2.0E+06
194	PC(16:0/O-16:0)	[M+K]+	C40H82NO7P	758.54605	758.54577	-0.4	2.9E+06
195	Myxochromide S1	[M+K]+	C38H54N6O8	761.36347	761.36457	1.4	5.2E+06
196	DG(22:1(13Z)/22:6(4Z,7Z,10Z,13Z,16Z,19Z)/0:0)	[M+K]+	C47H78O5	761.54809	761.54865	0.7	4.3E+06
197	1-Hydroxyvitamin D3 cellobioside	[M+K]+	C39H64O12	763.40294	763.40498	2.7	2.4E+06
198	PG(13:0/20:5(5Z,8Z,11Z,14Z,17Z))	[M+K]+	C39H67O10P	765.41034	765.40849	-2.4	7.2E+06
199	Mycolactone	[M+Na]+	C44H70O9	765.49120	765.49264	1.9	4.4E+07
200	MGDG(18:5(3Z,6Z,9Z,12Z,15Z)/18:4(6Z,9Z,12Z,15Z))	[M+H]+	C45H68O10	769.48852	769.49049	2.6	1.6E+06
201	PG(14:1(9Z)/22:2(13Z,16Z))	[M+H]+	C42H77O10P	773.53271	773.53138	-1.7	3.6E+06
202	PE(20:3(5Z,8Z,11Z)/P-18:1(11Z))	[M+Na]+	C43H78NO7P	774.54081	774.54043	-0.5	2.2E+06
203	PI(P-16:0/15:1(9Z))	[M+H]+	C40H75O12P	779.50689	779.50882	2.5	1.9E+06
204	MGDG-O(16:3(7Z,10Z,13Z))	[M+Na]+	C43H68O11	783.46538	783.46357	-2.3	4.3E+06
205	1-(ladderane-hexanoyl)-2-(ladderane-octanyl)-sn-glycerophosphoethanolamine	[M+K]+	C43H72NO7P	784.46780	784.46574	-2.6	1.6E+06
206	PG(O-16:0/22:4(7Z,10Z,13Z,16Z))	[M+H]+	C44H81O9P	785.56910	785.56776	-1.7	3.0E+06
207	PG(O-16:0/20:4(5Z,8Z,11Z,14Z))	[M+K]+	C42H77O9P	795.49368	795.49303	-0.8	2.5E+06
208	GalCer(d18:0/20:0)	[M+K]+	C44H87NO8	796.60633	796.60572	-0.8	1.1E+06

#	Putative Annotation (M)[a]	Ion	Formula	Theor. *m/z*[b]	Exp. *m/z*[c]	ppm[d]	Peak_Height
209	PG(O-16:0/20:3(8Z,11Z,14Z))	[M+K]+	C42H79O9P	797.50933	797.50929	-0.1	1.2E+06
210	PG(13:0/22:2(13Z,16Z))	[M+K]+	C41H77O10P	799.48859	799.48946	1.1	1.4E+06
211	PG(17:0/20:4(5Z,8Z,11Z,14Z))	[M+H]+	C43H80NO10P	802.55926	802.56084	2.0	3.1E+06
212	PG(14:1(9Z)/22:6(4Z,7Z,10Z,13Z,16Z,19Z))	[M+K]+	C42H69O10P	803.42599	803.42543	-0.7	4.6E+06
213	PC(16:1(7Z)/22:6(4Z,7Z,10Z,13Z,16Z,19Z))	[M+H]+	C46H78NO8P	804.55378	804.55613	2.9	5.2E+06
214	*N*-Acetyl-leu-leu-leu-leu-leu-tyr-amide	[M+Na]+	C41H69N7O8	810.50998	810.51210	2.6	8.8E+06
215	PG(14:0/22:2(13Z,16Z))	[M+K]+	C42H79O10P	813.50424	813.50630	2.5	1.2E+06
216	6'-Hydroxysiphonaxanthin dodecenoate	[M+H]+	C53H80O6	813.60277	813.60147	-1.6	1.2E+06
217	PC(15:0/22:4(7Z,10Z,13Z,16Z))	[M+Na]+	C45H82NO8P	818.56703	818.56564	-1.7	1.7E+06
218	PI(13:0/20:1(11Z))	[M+H]+	C42H79O13P	823.53311	823.53554	3.0	1.1E+06
219	Trihydroxyspirostenyl O-alpha-L-rhamnopyranosyl-(1-2)-beta-D-glucopyranoside	[M+K]+	C40H66O15	825.40333	825.40413	1.0	3.3E+06
220	Oligomycin B	[M+Na]+	C45H72O12	827.49160	827.49246	1.0	2.1E+06
221	PG(16:0/22:0)	[M+Na]+	C44H87O10P	829.59291	829.59346	0.7	2.6E+06
222	PI(P-18:0/17:2(9Z,12Z))	[M+H]+	C44H81O12P	833.55384	833.55275	-1.3	2.1E+06
223	PA(22:0/22:6(4Z,7Z,10Z,13Z,16Z,19Z))	[M+K]+	C47H81O8P	843.53007	843.53109	1.2	9.6E+05
224	PC(19:1(9Z)/22:6(4Z,7Z,10Z,13Z,16Z,19Z))	[M+H]+	C49H84NO8P	846.60073	846.59977	-1.1	2.1E+06
225	PE(20:5(5Z,8Z,11Z,14Z,17Z)/22:6(4Z,7Z,10Z,13Z,16Z,19Z))	[M+K]+	C47H72NO8P	848.46271	848.46155	-1.4	1.0E+06
226	Thyroxine sulfate	[M+H]+	C15H11I4NO7S	857.65079	857.64999	-0.9	8.7E+05
227	PI(P-20:0/17:2(9Z,12Z))	[M+H]+	C46H85O12P	861.58514	861.58685	2.0	1.6E+06
228	PC(22:2(13Z,16Z)/P-18:1(11Z))	[M+K]+	C48H90NO7P	862.60865	862.60788	-0.9	1.1E+06
229	PGP(16:0/18:2(9Z,12Z))	[M+K]+	C40H76O13P2	865.43928	865.43753	-2.0	1.3E+06
230	PG(20:2(11Z,14Z)/22:6(4Z,7Z,10Z,13Z,16Z,19Z))	[M+Na]+	C48H79O10P	869.53031	869.52905	-1.4	2.6E+06
231	PI(15:1(9Z)/22:4(7Z,10Z,13Z,16Z))	[M+H]+	C46H79O13P	871.53311	871.53215	-1.1	1.2E+06
232	PG(O-20:0/21:0)	[M+K]+	C47H95O9P	873.63453	873.63346	-1.2	1.7E+06
233	PG(20:1(11Z)/22:4(7Z,10Z,13Z,16Z))	[M+Na]+	C48H85O10P	875.57726	875.57491	-2.7	8.8E+05
234	PGP(16:1(9Z)/20:4(5Z,8Z,11Z,14Z))	[M+K]+	C42H74O13P2	887.42363	887.42363	0.0	9.7E+05
235	PI(18:4(6Z,9Z,12Z,15Z)/18:4(6Z,9Z,12Z,15Z))	[M+K]+	C45H71O13P	889.42639	889.42660	0.2	9.6E+05
236	PI(15:0/22:6(4Z,7Z,10Z,13Z,16Z,19Z))	[M+Na]+	C46H77O13P	891.49940	891.49691	-2.8	1.1E+06
237	PGP(18:0/18:1(11Z))	[M+K]+	C42H82O13P2	895.48623	895.48553	-0.8	1.1E+06
238	PS(21:0/22:1(11Z))	[M+Na]+	C49H94NO10P	910.65076	910.64966	-1.2	9.8E+05

#	Putative Annotation (M)[a]	Ion	Formula	Theor. *m/z*[b]	Exp. *m/z*[c]	ppm[d]	Peak_Height
239	PI(18:3(6Z,9Z,12Z)/20:5(5Z,8Z,11Z,14Z,17Z))	[M+K]+	C47H75O13P	917.45769	917.45512	-2.8	1.4E+06
240	PI(19:0/22:2(13Z,16Z))	[M+H]+	C50H93O13P	933.64266	933.64068	-2.1	9.3E+05
241	PI(O-20:0/20:3(8Z,11Z,14Z))	[M+K]+	C49H91O12P	941.58797	941.58682	-1.2	1.3E+06

[a]Cer: Ceramide; GalCer: Galactosylceramide; GlcCer: Glucosylceramide; Glc-GP: phosphatidylglucose; LacCer: Lactosylceramide; MG: Monoacylglycerol; DAT: Acyltrehaloses; DG: Diacylglycerol; TG: Triacylglycerol; MGDG: Monoacyldiacylglycerol; PA: Phosphatidic acid; PC: Phosphatidylcholine; PE: Phosphatidylethanolamine; PG: Glycerophospholipids; PI: Phosphatidylinositol; PIP2: phosphatidylinositol bisphosphate; PS: Phosphatidylserine; SM: Sphingomyelin.
[b]Theor. stands for calculated exact mass to charge ratio.
[c]Exp. stands for experimental *m*/*z* value.
[d]The error expressed in parts per million (ppm).

Table S2. ESI FT-ICR MS comprehensive list of metabolites detected in pMVs extract.

#	Putative Annotation (M)[a]	Ion	Formula	Theor. *m/z*[b]	Exp. *m/z*[c]	ppm[d]	Peak_Height
1	Succinic acid	[M-H]-	C4H6O4	117.01933	117.01951	1.5	7.4E+05
2	(R)-Malate	[M-H]-	C4H6O5	133.01425	133.01426	0.1	2.9E+05
3	(*R*)-3-Hydroxy-3-methyl-2-oxopentanoate	[M-H]-	C6H10O4	145.05063	145.05051	-0.8	2.3E+05
4	D-Glutamate	[M-H]-	C5H9NO4	146.04588	146.04588	0.0	4.3E+06
5	(*R*)-2-Hydroxyglutarate	[M-H]-	C5H8O5	147.02990	147.02955	-2.4	2.7E+05
6	Triethanolamine	[M+H]+	C6H15NO3	150.11247	150.11243	-0.3	5.4E+05
7	Diethylphosphate	[M-H]-	C4H11O4P	153.03222	153.03198	-1.6	4.8E+05
8	5-Hydroxypentanoate	[M+Cl]-	C5H10O3	153.03240	153.03198	-2.7	4.8E+05
9	D-Glutamic acid	[M+Na]+	C5H9NO4	170.04238	170.04202	-2.1	4.3E+05
10	L-Arginine	[M+H]+	C6H14N4O2	175.11895	175.11888	-0.4	9.4E+06
11	*cis*-2-Carboxycyclohexyl-acetic acid	[M-H]-	C9H14O4	185.08193	185.08150	-2.3	3.0E+05
12	Quinic acid	[M-H]-	C7H12O6	191.05611	191.05610	-0.1	2.1E+06
13	2-Aminomuconate	[M+Cl]-	C6H7NO4	192.00691	192.00714	1.2	3.2E+05
14	3-Hydroxy-L-glutamate	[M+K]+	C5H9NO5	202.01123	202.01183	3.0	4.5E+05
15	Aldohexose	[M+Na]+	C6H12O6	203.05261	203.05271	0.5	6.8E+06
16	Mannitol	[M+Na]+	C6H14O6	205.06826	205.06859	1.6	3.7E+05
17	(+)-3-hydroxy pelargonic acid	[M+Cl]-	C9H18O3	209.09500	209.09442	-2.8	6.4E+05
18	Aldohexose	[M+Cl]-	C6H12O6	215.03279	215.03218	-2.8	8.8E+05
19	Myristic acid	[M-H]-	C14H28O2	227.20175	227.20165	0.4	8.5E+05
20	Sinapyl alcohol	[M+Na]+	C11H14O4	233.07843	233.07905	2.7	7.2E+05
21	Dimethylenetriurea	[M+Cl]-	C5H12N6O3	239.06649	239.06706	2.4	3.9E+05
22	Glutinosone	[M+Na]+	C14H20O2	243.13555	243.13572	0.7	3.8E+05
23	1-Octylglycerol	[M+K]+	C11H24O3	243.13570	243.13572	0.1	3.8E+05
24	1,3,7-Trimethyluric acid	[M+Cl]-	C8H10N4O3	245.04469	245.04514	1.8	2.6E+05
25	Pimpinellin	[M-H]-	C13H10O5	245.04555	245.04514	-1.7	2.6E+05
26	2E,4E,8E,10E-Dodecatetraenedioic acid	[M+Na]+	C12H14O4	245.07843	245.07862	0.8	4.0E+05
27	Encecalin	[M+Na]+	C14H16O3	255.09917	255.09924	0.3	5.6E+05
28	Undecanedioic acid	[M+K]+	C11H20O4	255.09932	255.09997	2.6	5.4E+05
29	Palmitic acid	[M-H]-	C16H32O2	255.23295	255.23271	-1.0	2.2E+06

#	Putative Annotation (M)[a]	Ion	Formula	Theor. *m/z*[b]	Exp. *m/z*[c]	ppm[d]	Peak_Height
30	6-Hydroxyl-1,6-dihydropurine ribonucleoside	[M-H]-	C10H14N4O5	269.08914	269.08850	-2.4	1.0E+06
31	(+)-15S-hydroxy-hexadecanoic acid	[M-H]-	C16H32O3	271.22787	271.22718	-2.5	2.8E+05
32	11-hydroxy palmitic acid	[M-H]-	C16H32O3	271.22787	271.22718	-2.5	2.8E+05
33	1,2-Bis(4-nitrophenyl)ethane	[M+H]+	C14H12N2O4	273.08698	273.08770	2.6	6.4E+05
34	Oleic acid	[M-H]-	C18H34O2	281.24860	281.24894	1.2	4.4E+06
35	(11E)-Octadecenoic acid	[M+H]+	C18H34O2	283.26316	283.26290	-0.9	5.3E+05
36	Stearic acid	[M-H]-	C18H36O2	283.26425	283.26489	2.2	2.0E+06
37	*N*-Glycosyl-L-asparagine	[M-H]-	C10H18N2O8	293.09904	293.09827	-2.6	1.1E+06
38	(*R*)-10-Hydroxystearate	[M-H]-	C18H36O3	299.25917	299.25922	0.2	2.5E+06
39	Dibutyl phthalate	[M+Na]+	C16H22O4	301.14103	301.14078	-0.8	3.1E+06
40	MG(0:0/14:0/0:0)	[M-H]-	C17H34O4	301.23843	301.23780	-2.1	6.8E+05
41	3"-Deamino-3"-oxonicotianamine	[M+H]+	C12H18N2O7	303.11868	303.11817	-1.7	5.9E+05
42	Linoleic acid	[M+Na]+	C18H32O2	303.22945	303.23007	2.0	2.0E+06
43	alpha-Obscurine	[M+Cl]-	C17H26N2O	309.17392	309.17482	2.9	4.5E+05
44	17beta-Hydroxy-3-methoxyestra-1,3,5(10)-triene-17-carbonitrile	[M-H]-	C20H25NO2	310.18125	310.18053	-2.3	3.4E+05
45	Spiradine A	[M-H]-	C20H25NO2	310.18125	310.18053	-2.3	3.4E+05
46	Phytosphingosine	[M+H]+	C18H39NO3	318.30027	318.30049	0.7	3.3E+06
47	1-Carbazol-9-yl-3-(3,5-dimethylpyrazol-1-yl)-propan-2-ol	[M+H]+	C20H21N3O	320.17574	320.17597	0.7	1.3E+06
48	16-oxo-octadecanoic acid	[M+Na]+	C18H34O3	321.24002	321.24022	0.6	6.8E+06
49	(*R*)-10-Hydroxystearate	[M+Na]+	C18H36O3	323.25567	323.25537	-0.9	4.9E+07
50	Galactosylhydroxylysine	[M+H]+	C12H24N2O8	325.16054	325.15984	-2.2	8.3E+05
51	Tributyrin	[M+Na]+	C15H26O6	325.16216	325.16285	2.1	8.5E+05
52	(Z)-11beta,21-Dihydroxypregna-1,4,17(20)-trien-3-one	[M+H]+	C21H28O3	329.21112	329.21040	-2.2	1.3E+06
53	Lipoyllysine	[M+H]+	C14H26N2O3S2	335.14576	335.14657	2.4	1.3E+06
54	(R)-10-Hydroxystearate	[M+Cl]-	C18H36O3	335.23585	335.23600	0.5	3.3E+05
55	(9Z)-(7S,8S)-Dihydroxyoctadecenoic acid	[M+Na]+	C18H34O4	337.23493	337.23533	1.2	3.1E+06
56	(20R)-20-hydroxypregn-4-en-3-one	[M+Na]+	C21H32O2	339.22945	339.22926	-0.6	3.3E+06
57	Palustrine	[M+K]+	C17H31N3O2	348.20479	348.20567	2.5	9.0E+05
58	2-hydroxy-eicosanoic acid	[M+Na]+	C20H40O3	351.28697	351.28738	1.2	1.2E+06
59	Biflorin	[M-H]-	C16H18O9	353.08781	353.08775	-0.2	5.6E+07

#	Putative Annotation (M)[a]	Ion	Formula	Theor. *m/z*[b]	Exp. *m/z*[c]	ppm[d]	Peak_Height
60	MG(0:0/16:0/0:0)	[M+Na]+	C19H38O4	353.26623	353.26639	0.5	1.1E+07
61	6,7-Dimethyl-8-(1-D-ribityl)lumazine	[M+Cl]-	C13H18N4O6	361.09204	361.09281	2.1	4.3E+05
62	5,7,3'-Trihydroxy-6,4',5'-trimethoxyflavanone	[M-H]-	C18H18O8	361.09289	361.09281	-0.2	4.3E+05
63	5-Hydroxy-3',4'-methylenedioxy-6",6"-dimethylpyrano[2",3":7,8]isoflavone	[M+H]+	C21H16O6	365.10196	365.10164	-0.9	1.7E+06
64	(+)-18-methyl-eicosanoic acid	[M+K]+	C21H42O2	365.28164	365.28119	-1.2	8.2E+05
65	(2S)-5,7-Dimethoxy-3',4'-methylenedioxyflavanone	[M+K]+	C18H16O6	367.05785	367.05864	2.2	1.6E+06
66	3,4-Dimethoxylonchocarpin	[M+H]+	C22H22O5	367.15400	367.15379	-0.6	8.6E+05
67	(3S,4S)-3-Hydroxytetradecane-1,3,4-tricarboxylate	[M+Na]+	C17H30O7	369.18837	369.18749	-2.4	1.2E+06
68	3-Oxochola-1,4,6-trien-24-oic Acid	[M+H]+	C24H32O3	369.24242	369.24198	-1.2	9.1E+05
69	Sativic acid	[M+Na]+	C18H36O6	371.24041	371.24059	0.5	8.3E+05
70	17beta-(Acetylthio)estra-1,3,5(10)-trien-3-ol acetate	[M+H]+	C22H28O3S	373.18319	373.18209	-3.0	2.5E+06
71	2,3-di-*O*-hexanoyl-alpha-glucopyranose	[M+H]+	C18H32O8	377.21699	377.21615	-2.2	8.7E+05
72	1alpha,5alpha-Dimercaptoandrostane-3alpha,17beta-diol	[M+Na]+	C19H32O2S2	379.17359	379.17312	-1.2	1.0E+07
73	9,10-Dihydrokadsurenone	[M+Na]+	C21H26O5	381.16724	381.16735	0.3	7.8E+05
74	2,3-Dinor-6-keto-prostaglandin F1 a	[M+K]+	C18H30O6	381.16740	381.16735	-0.1	7.8E+05
75	MG(0:0/18:0/0:0)	[M+Na]+	C21H42O4	381.29753	381.29751	-0.1	1.0E+07
76	16-Methoxy-2,3-dihydro-3-hydroxytabersonine	[M-H]-	C22H28N2O4	383.19763	383.19864	2.6	3.9E+05
77	11-deoxy-11-methylene-15-keto-PGD2	[M+Cl]-	C21H32O4	383.19946	383.19864	-2.1	3.9E+05
78	Macrozamin	[M+H]+	C13H24N2O11	385.14529	385.14416	-2.9	1.0E+06
79	6-Deoxyerythronolide B	[M+H]+	C21H38O6	387.27412	387.27477	1.7	1.2E+06
80	N,N-(2,2-dihydroxy-ethyl)arachidonoyl amine	[M-H]-	C24H41NO3	390.30137	390.30104	-0.8	8.2E+05
81	11-Dehydro-thromboxane B2	[M+Na]+	C20H32O6	391.20911	391.20957	1.2	1.3E+06
82	5,7,3',4'-Tetrahydroxy-8-prenylflavone	[M+K]+	C20H18O6	393.07350	393.07449	2.5	2.7E+06
83	9'-carboxy-gamma-tocotrienol	[M+Na]+	C23H31O4	394.21146	394.21234	2.2	1.2E+07
84	3-Deoxyvitamin D3	[M+Cl]-	C27H44	403.31370	403.31433	1.6	4.7E+05
85	3',5'-Diprenylgenistein	[M+H]+	C25H26O5	407.18530	407.18519	-0.3	7.6E+06
86	Propantheline	[M+K]+	C23H30NO3	407.18573	407.18519	-1.3	7.6E+06
87	9'-Carboxy-alpha-tocotrienol	[M+Na]+	C24H33O4	408.22711	408.22792	2.0	8.3E+06
88	1-Isomangostin	[M+H]+	C24H26O6	411.18022	411.18027	0.1	1.3E+06
89	Rehmaionoside A	[M+Na]+	C19H34O8	413.21459	413.21340	-2.9	2.0E+06

#	Putative Annotation (M)[a]	Ion	Formula	Theor. *m/z*[b]	Exp. *m/z*[c]	ppm[d]	Peak_Height
90	(22E)-3alpha,12alpha-Dihydroxy-5beta-chol-22-en-24-oic Acid	[M+Na]+	C24H38O4	413.26623	413.26655	0.8	3.4E+06
91	25-azavitamin D3	[M+Cl]-	C26H43NO	420.30387	420.30360	-0.6	7.5E+05
92	(2S)-5,7,2',4'-Tetrahydroxy-8-prenyl-5'-(1,1-dimethylallyl)flavanone	[M+H]+	C25H28O6	425.19587	425.19690	2.4	1.7E+06
93	LysoPC(10:0)	[M+Na]+	C18H39NO7P	435.23563	435.23535	-0.7	1.6E+06
94	Lunarine	[M+H]+	C25H31N3O4	438.23873	438.23919	1.0	1.3E+07
95	(24E)-24,26-dimethyldesmosterol	[M+Cl]-	C29H48O	447.33992	447.34092	2.2	2.8E+05
96	3'-Prenylrubranine	[M+H]+	C30H34O4	459.25299	459.25432	2.9	1.9E+06
97	Artonin E	[M+Cl]-	C25H24O7	471.12160	471.12087	-1.6	2.7E+05
98	Glabrescione B	[M+Na]+	C27H30O6	473.19346	473.19458	2.4	1.3E+06
99	Glutathionylspermidine	[M+K]+	C17H34N6O5S	473.19430	473.19503	1.5	1.3E+06
100	7-O-Methyllicoricidin	[M+K]+	C27H34O5	477.20378	477.20471	1.9	2.4E+06
101	Gentamicin X2	[M+H]+	C19H38N4O10	483.26607	483.26733	2.6	2.4E+06
102	Microlenin	[M+H]+	C29H34O7	495.23773	495.23727	-0.9	8.3E+06
103	3-Sulfodeoxycholic acid	[M+Na]+	C24H40O7S	495.23870	495.23727	-2.9	8.3E+06
104	1-O-all-trans-retinoyl-beta-glucuronic acid	[M+Na]+	C26H36O8	499.23024	499.23170	2.9	1.3E+06
105	Drummondin A	[M+H]+	C28H34O8	499.23264	499.23244	-0.4	1.6E+06
106	PC(P-15:0/0:0)	[M+K]+	C23H48NO6P	504.28508	504.28423	-1.7	2.1E+06
107	3-Geranyl-4,2',4',6'-tetrahydroxy-5-prenyldihydrochalcone	[M+K]+	C30H38O5	517.23508	517.23651	2.8	1.9E+06
108	Mycalamide B	[M+H]+	C25H43NO10	518.29597	518.29704	2.1	1.4E+06
109	3-*O*-L-rhamnosyl-3-hydroxydecanoyl-3-hydroxydecanoic acid	[M+H]+	C27H50O9	519.35276	519.35295	0.4	2.2E+06
110	LysoPE(0:0/20:3(11Z,14Z,17Z))	[M+Na]+	C25H46NO7P	526.29041	526.29007	-0.6	8.8E+06
111	LysoPC(18:3(6Z,9Z,12Z))	[M+Na]+	C26H48NO7P	540.30606	540.30489	-2.2	7.7E+06
112	Cucurbitacin F	[M+Na]+	C30H46O7	541.31357	541.31274	-1.5	2.2E+06
113	dolichyl diphosphate	[M+Na]+	C25H46O7P2	543.26110	543.26123	0.2	1.5E+06
114	2alpha-(benzyloxy)-1alpha,25-dihydroxy-19-norvitamin D3	[M+Cl]-	C33H50O4	545.34031	545.34111	1.5	2.9E+05
115	1-octadecyl-11E-hexadecenoate	[M+K]+	C34H66O2	545.46944	545.47005	1.1	1.8E+06
116	Coumermic acid	[M-H]-	C27H21N3O10	546.11542	546.11523	-0.3	2.6E+05
117	LysoPE(0:0/20:1(11Z))	[M+K]+	C25H50NO7P	546.29565	546.29467	-1.8	2.5E+06
118	3-O-Mycarosylerythronolide B	[M+H]+	C28H50O10	547.34767	547.34856	1.6	3.0E+06
119	LysoPC(17:0)	[M+K]+	C25H52NO7P	548.31130	548.31178	0.9	2.4E+06

#	Putative Annotation (M)[a]	Ion	Formula	Theor. *m/z*[b]	Exp. *m/z*[c]	ppm[d]	Peak_Height
120	(+)-Myristinin A	[M+H]+	C33H40O7	549.28468	549.28565	1.8	2.5E+06
121	Enkephalin L	[M+H]+	C28H37N5O7	556.27658	556.27760	1.8	4.1E+06
122	PG(22:4(7Z,10Z,13Z,16Z)/0:0)	[M+H]+	C28H49O9P	561.31870	561.31999	2.3	4.6E+06
123	1-Oleoylglycerophosphocholine	[M+K]+	C26H53NO7P	561.31912	561.31999	1.5	4.6E+06
124	LysoPE(0:0/22:4(7Z,10Z,13Z,16Z))	[M+K]+	C27H48NO7P	568.28000	568.28152	2.7	2.1E+06
125	PC(O-18:0/O-2:0)	[M+K]+	C28H60NO6P	576.37898	576.37812	-1.5	1.3E+06
126	6,8a-Seco-6,8a-deoxy-5-oxoavermectin "1b" aglycone	[M+Na]+	C33H46O7	577.31357	577.31461	1.8	2.8E+06
127	Prednisolone 21-all-cis-farnesylate	[M+H]+	C36H50O6	579.36802	579.36906	1.8	4.6E+06
128	Dihydromethanophenazine	[M+K]+	C37H52N2O	579.37112	579.37014	-1.7	4.6E+06
129	2-deoxy-20-hydroxyecdysone 22-phosphate	[M+K]+	C27H45O9P	583.24328	583.24213	-2.0	3.5E+06
130	5-Oxoavermectin "1a" aglycone	[M+H]+	C34H46O8	583.32654	583.32712	1.0	1.9E+07
131	(3Z)-Phycocyanobilin	[M+H]+	C33H38N4O6	587.28641	587.28549	-1.6	1.3E+06
132	LysoPC(22:6(4Z,7Z,10Z,13Z,16Z,19Z))	[M+Na]+	C30H50NO7P	590.32171	590.32076	-1.6	2.3E+06
133	PC(16:0/3:0)	[M+K]+	C27H54NO8P	590.32186	590.32076	-1.9	2.3E+06
134	LysoPC(22:5(4Z,7Z,10Z,13Z,16Z))	[M+Na]+	C30H52NO7P	592.33736	592.33812	1.3	2.0E+06
135	Docosyl-palmitate	[M+Cl]-	C38H76O2	599.55393	599.55252	-2.4	2.7E+06
136	3-O-acetylecdysone 2-phosphate	[M+Na]+	C29H47O10P	609.27991	609.28142	2.5	2.7E+06
137	Avermectin B1b aglycone	[M+K]+	C33H46O8	609.28243	609.28183	-1.0	2.7E+06
138	7,8,7',8'-Tetradehydroastaxanthin	[M+Na]+	C40H48O4	615.34448	615.34602	2.5	1.1E+06
139	PC(O-12:0/O-12:0)	[M+Na]+	C32H68NO6P	616.46765	616.46640	-2.0	1.2E+06
140	DG(14:0/22:4(7Z,10Z,13Z,16Z)/0:0)	[M+H]+	C39H68O5	617.51395	617.51550	2.5	1.1E+06
141	5-Oxoavermectin "2b" aglycone	[M+K]+	C33H46O9	625.27734	625.27694	-0.6	1.3E+06
142	Gambogic acid	[M+H]+	C38H44O8	629.31089	629.31050	-0.6	5.7E+06
143	Mancinellin	[M+Na]+	C36H52O8	635.35544	635.35524	-0.3	2.8E+06
144	26-O-[beta-D-glucopyranosyl]-25R-furostan-3beta,22alpha,26-triol	[M+K]+	C33H56O9	635.35559	635.35524	-0.6	2.8E+06
145	Avermectin A2b aglycone	[M+K]+	C34H50O9	641.30864	641.30908	0.7	1.7E+06
146	6-O-(Glcb)-(25R)-5alpha-spirostan-3beta,6alpha,23S-triol	[M+Na]+	C34H58O10	649.39222	649.39351	2.0	5.2E+06
147	PI(22:2(13Z,16Z)/0:0)	[M-H]-	C31H57O12P	651.35149	651.35238	1.4	2.7E+05
148	Kurilensoside H	[M+K]+	C32H54O11	653.32977	653.33143	2.5	2.7E+06
149	Gnididilatin	[M+H]+	C37H48O10	653.33202	653.33320	1.8	2.9E+06

#	Putative Annotation (M)[a]	Ion	Formula	Theor. *m/z*[b]	Exp. *m/z*[c]	ppm[d]	Peak_Height
150	PA(13:0/18:2(9Z,12Z))	[M+Na]+	C34H63O8P	653.41528	653.41417	-1.7	1.7E+06
151	PG(12:0/16:1(9Z))	[M+H]+	C34H65O10P	665.43881	665.44072	2.9	2.6E+06
152	1-(O-alpha-D-galactopyranosyl)-3-keto-(1,27R,29R)-triacontanetriol	[M+Na]+	C36H70O9	669.49120	669.49061	-0.9	1.1E+06
153	1-(O-alpha-D-galactopyranosyl)-(1,3R,27S,29R)-triacontanetetrol	[M+Na]+	C36H72O9	671.50685	671.50816	1.9	1.5E+06
154	SM(d18:0/12:0)	[M+Na]+	C35H73N2O6P	671.50985	671.50816	-2.5	1.5E+06
155	Pandaroside B	[M+K]+	C35H54O10	673.33486	673.33538	0.8	4.0E+06
156	PI(22:2(13Z,16Z)/0:0)	[M+Na]+	C31H57O12P	675.34798	675.34693	-1.6	1.2E+06
157	PG(12:0/17:2(9Z,12Z))	[M+H]+	C35H65O10P	677.43881	677.43866	-0.2	4.5E+06
158	Purpureacin-1	[M+K]+	C37H66O8	677.43893	677.43866	-0.4	4.5E+06
159	Abrusoside A	[M+K]+	C36H54O10	685.33486	685.33681	2.8	1.5E+06
160	1-Palmitoyl-2-(5-hydroxy-8-oxo-6-octenoyl)-sn-glycero-3-phosphatidylcholine	[M+K]+	C32H60NO10P	688.35864	688.35682	-2.6	1.4E+06
161	methyl 13-sophorosyloxydocosanoate	[M+H]+	C35H66O13	695.45762	695.45841	1.1	1.5E+06
162	Sanggenon D	[M-H]-	C40H36O12	707.21340	707.21154	-2.6	3.8E+06
163	PE(14:1(9Z)/P-18:1(11Z))	[M+K]+	C37H70NO7P	710.45215	710.45107	-1.5	1.3E+06
164	DG(20:3(5Z,8Z,11Z)/22:6(4Z,7Z,10Z,13Z,16Z,19Z)/0:0)	[M+Na]+	C45H70O5	713.51155	713.51179	0.3	1.8E+06
165	PA(15:1(9Z)/22:2(13Z,16Z))	[M+H]+	C40H73O8P	713.51158	713.51179	0.3	1.8E+06
166	DG(22:6(4Z,7Z,10Z,13Z,16Z,19Z)/22:6(4Z,7Z,10Z,13Z,16Z,19Z)/0:0)	[M+H]+	C47H68O5	713.51395	713.51402	0.1	1.9E+06
167	PC(12:0/18:3(6Z,9Z,12Z))	[M+Na]+	C38H70NO8P	722.47313	722.47171	-2.0	1.2E+06
168	PA(O-16:0/20:3(8Z,11Z,14Z))	[M+K]+	C39H73O7P	723.47255	723.47460	2.8	1.0E+06
169	PS(P-16:0/17:2(9Z,12Z))	[M-H]-	C39H72NO9P	728.48719	728.48532	-2.6	3.5E+05
170	Quercetin 3-(3",4"-diacetylrhamnosyl)-(1->6)-glucoside	[M+Cl]-	C31H34O18	729.14392	729.14496	1.4	2.4E+05
171	OH-Chlorobactene glucoside ester	[M+H]+	C47H68O6	729.50887	729.51004	1.6	7.3E+05
172	PG(13:0/20:1(11Z))	[M+H]+	C39H75O10P	735.51706	735.51789	1.1	5.1E+06
173	PC(14:1(9Z)/P-18:1(11Z))	[M+Na]+	C40H76NO7P	736.52516	736.52315	-2.7	2.1E+06
174	Delavaine A	[M+Na]+	C38H54N2O11	737.36198	737.36391	2.6	2.7E+06
175	PI(12:0/12:0)	[M+K]+	C33H63O13P	737.36379	737.36391	0.2	2.7E+06
176	PC(12:0/18:2(9Z,12Z))	[M+K]+	C38H72NO8P	740.46271	740.46189	-1.1	1.1E+06
177	Spinosyn P	[M+K]+	C39H61NO10	742.39271	742.39078	-2.6	1.0E+06
178	PA(15:0/22:6(4Z,7Z,10Z,13Z,16Z,19Z))	[M+K]+	C40H67O8P	745.42052	745.41828	-3.0	3.2E+06
179	Chikusetsusaponin Ia	[M+H]+	C41H70O12	755.49400	755.49187	-2.8	7.2E+05

#	Putative Annotation (M)[a]	Ion	Formula	Theor. *m/z*[b]	Exp. *m/z*[c]	ppm[d]	Peak_Height
180	PA(O-16:0/22:0)	[M+K]+	C41H83O7P	757.55080	757.54956	-1.6	1.2E+06
181	Avermectin A1a monosaccharide	[M+Na]+	C42H62O11	765.41843	765.41616	-3.0	1.8E+06
182	Nonactin	[M+K]+	C40H64O12	775.40294	775.40293	0.0	1.4E+06
183	Mycolactone	[M+K]+	C44H70O9	781.46514	781.46564	0.6	1.1E+06
184	PA(P-20:0/22:6(4Z,7Z,10Z,13Z,16Z,19Z))	[M+Cl]-	C45H77O7P	795.51009	795.50948	-0.8	3.9E+05
185	PG(14:0/22:0)	[M+Na]+	C42H83O10P	801.56161	801.56260	1.2	8.9E+05
186	PG(16:1(9Z)/22:2(13Z,16Z))	[M+H]+	C44H81O10P	801.56401	801.56260	-1.8	8.9E+05
187	Trihydroxyspirosten-yl O-L-rhamnopyranosyl-(1-2)-beta-D-glucopyranoside	[M+Na]+	C40H66O15	809.42939	809.43117	2.2	1.0E+06
188	PI(13:0/18:3(6Z,9Z,12Z))	[M+Na]+	C40H71O13P	829.42639	829.42742	1.2	1.3E+06
189	Gymnemic acid I	[M+K]+	C43H66O14	845.40842	845.40879	0.4	7.4E+05
190	PI(12:0/20:1(11Z))	[M+K]+	C41H77O13P	847.47334	847.47198	-1.6	7.8E+05
191	PS(P-20:0/22:4(7Z,10Z,13Z,16Z))	[M+H]+	C48H86NO9P	852.61130	852.61364	2.7	1.1E+06
192	PI(17:0/22:0)	[M+H]+	C48H93O13P	909.64266	909.64511	2.7	1.8E+06
193	PS(21:0/22:1(11Z))	[M+Na]+	C49H94NO10P	910.65076	910.65108	0.4	9.7E+05
194	PGP(16:0/22:4(7Z,10Z,13Z,16Z))	[M+K]+	C44H80O13P2	917.47058	917.47086	0.3	5.7E+05
195	PS(22:2(13Z,16Z)/22:0)	[M+Cl]-	C50H94NO10P	934.63094	934.63278	2.0	5.3E+05
196	PGP(18:3(6Z,9Z,12Z)/22:6(4Z,7Z,10Z,13Z,16Z,19Z))	[M+K]+	C46H74O13P2	935.42363	935.42349	-0.1	8.1E+05
197	Ophiopogonin C	[M+K]+	C46H72O17	935.44011	935.43736	-2.9	1.0E+06
198	TG(17:2(9Z,12Z)/20:4(5Z,8Z,11Z,14Z)/20:5(5Z,8Z,11Z,14Z,17Z))	[M+Cl]-	C60H94O6	945.67444	945.67184	-2.8	3.7E+05

[a]Cer: Ceramide; GalCer: Galactosylceramide; GlcCer: Glucosylceramide; Glc-GP: phosphatidylglucose; LacCer: Lactosylceramide; MG: Monoacylglycerol; DAT: Acyltrehaloses; DG: Diacylglycerol; TG: Triacylglycerol; MGDG: Monoacyldiacylglycerol; PA: Phosphatidic acid; PC: Phosphatidylcholine; PE: Phosphatidylethanolamine; PG: Glycerophospholipids; PI: Phosphatidylinositol; PIP2: phosphatidylinositol bisphosphate; PS: Phosphatidylserine; SM: Sphingomyelin.
[b]Theor. stands for calculated exact mass to charge ratio.
[c]Exp. stands for experimental *m*/*z* value.
[d]The error expressed in parts per million (ppm).

Table S3. ESI FT-ICR MS comprehensive list of metabolites detected in SurE 10K.

#	Putative Annotation (M)[a]	Ion	Formula	Theor. *m/z*[b]	Exp. *m/z*[c]	ppm[d]	Peak_Height
1	L-Lactic acid	[M-H]-	C3H6O3	89.02442	89.02430	-1.3	2.2E+04
2	Acrolein	[M+Cl]-	C3H4O	90.99562	90.99559	-0.3	1.5E+04
3	Vinyl ether	[M+Na]+	C4H6O	93.03109	93.03099	-1.0	4.8E+04
4	Acrylamide	[M+Na]+	C3H5NO	94.02633	94.02658	2.6	5.3E+04
5	2-Propyn-1-ol	[M+K]+	C3H4O	94.98937	94.98962	2.6	4.1E+04
6	2-Methylpropanamine	[M+Na]+	C4H11N	96.07837	96.07818	-2.0	5.0E+04
7	Sulfate	[M-H]-	H2SO4	96.96010	96.96038	2.9	2.8E+04
8	Trimethylamine	[M+K]+	C3H9N	98.03666	98.03654	-1.2	6.5E+04
9	1-Amino-propan-2-ol	[M+Na]+	C3H9NO	98.05763	98.05752	-1.2	6.1E+04
10	Hydroxyurea	[M+Na]+	CH4N2O2	99.01650	99.01654	0.4	3.5E+04
11	Propan-2-ol	[M+K]+	C3H8O	99.02067	99.02067	0.0	3.9E+04
12	Propane-1-thiol	[M+Cl]-	C3H8S	111.00407	111.00393	-1.3	1.7E+04
13	Butanal	[M+K]+	C4H8O	111.02067	111.02070	0.2	4.1E+04
14	*N*-Acetylimidazole	[M+H]+	C5H6N2O	111.05529	111.05549	1.8	4.2E+04
15	Maleamate	[M-H]-	C4H5NO3	114.01967	114.01934	-2.9	1.9E+04
16	3-Mercaptopyruvic acid	[M-H]-	C3H4O3S	118.98084	118.98119	3.0	1.9E+04
17	D-Erythrose	[M+H]+	C4H8O4	121.04954	121.04931	-1.9	3.5E+04
18	Thymine	[M-H]-	C5H6N2O2	125.03565	125.03545	-1.6	2.2E+04
19	2,3,6-Trihydroxypyridine	[M-H]-	C5H5NO3	126.01967	126.01980	1.1	2.0E+04
20	2-Cyanopyridine	[M+Na]+	C6H4N2	127.02667	127.02654	-1.0	3.9E+04
21	Furfural	[M+Cl]-	C5H4O2	130.99053	130.99019	-2.6	3.0E+04
22	Cinnamaldehyde	[M-H]-	C9H8O	131.05024	131.05001	-1.7	2.7E+04
23	Cyclohexyl isocyanide	[M+Na]+	C7H11N	132.07837	132.07804	-2.5	3.8E+04
24	L-Malic acid	[M-H]-	C4H6O5	133.01425	133.01436	0.9	2.0E+05
25	Thiobenzamide	[M-H]-	C7H7NS	136.02264	136.02251	-1.0	1.7E+04
26	Threonic acid	[M+H]+	C4H8O5	137.04445	137.04477	2.3	4.4E+04
27	Pyruvate oxime	[M+Cl]-	C3H5NO3	137.99634	137.99660	1.9	3.3E+04
28	6-Hydroxynicotinate	[M-H]-	C6H5NO3	138.01967	138.01928	-2.8	2.6E+04
29	L-Proline	[M+Na]+	C5H9NO2	138.05255	138.05277	1.6	4.1E+04
30	Tyramine	[M+H]+	C8H11NO	138.09134	138.09173	2.8	7.8E+04
31	Cyclohexanol	[M+K]+	C6H12O	139.05197	139.05228	2.2	3.9E+04
32	8-Hydroxypurine	[M+H]+	C5H6N4O	139.06144	139.06135	-0.6	4.0E+04
33	Diethanolamine	[M+Cl]-	C4H11NO2	140.04838	140.04803	-2.5	2.4E+04
34	Thiocarbohydrazide	[M+Cl]-	CH6N4S	141.00072	141.00039	-2.3	1.8E+04
35	2-decene-4,6,8-triyn-1-al	[M-H]-	C10H6O	141.03459	141.03422	-2.6	2.5E+04
36	1-Hexenyl acetate	[M-H]-	C8H14O2	141.09210	141.09233	1.6	2.8E+04

37	5-Nitrofurfural	[M+H]+	C5H3NO4	142.01348	142.01361	0.9	4.3E+04
38	Proline betaine	[M-H]-	C7H13NO2	142.08735	142.08756	1.5	5.3E+04
39	(-)-Hygroline	[M-H]-	C8H17NO	142.12374	142.12339	-2.4	4.5E+04
40	Diallyl disulfide	[M-H]-	C6H10S2	145.01512	145.01481	-2.1	2.6E+04
41	N-Acetylimidazole	[M+Cl]-	C5H6N2O	145.01741	145.01773	2.2	3.4E+04
42	D-Threitol	[M+Na]+	C4H10O4	145.04713	145.04706	-0.5	4.4E+04
43	L-Lysine	[M-H]-	C6H14N2O2	145.09825	145.09868	3.0	2.1E+04
44	L-Glutamic acid	[M-H]-	C5H9NO4	146.04588	146.04586	-0.1	1.3E+05
45	Maleamate	[M+Cl]-	C4H5NO3	149.99634	149.99614	-1.4	1.9E+04
46	Histamine	[M+K]+	C5H9N3	150.04281	150.04313	2.2	5.0E+04
47	L-Methionine	[M+H]+	C5H11NO2S	150.05833	150.05837	0.3	5.3E+04
48	N-Nitroso-1,3-thiazolidine	[M+Cl]-	C3H6N2OS	152.98949	152.98941	-0.5	1.3E+04
49	(+)-Borneol	[M-H]-	C10H18O	153.12849	153.12861	0.8	1.6E+04
50	Leucine	[M+Na]+	C6H13NO2	154.08385	154.08361	-1.6	2.2E+05
51	3-Mercaptopyruvic acid	[M+Cl]-	C3H4O3S	154.95752	154.95775	1.5	1.5E+04
52	1,2,3-Trimethylbenzene	[M+Cl]-	C9H12	155.06330	155.06307	-1.5	1.8E+04
53	5-oxo-7-octenoic acid	[M-H]-	C8H12O3	155.07137	155.07146	0.6	3.0E+04
54	3Z-nonenoic acid	[M-H]-	C9H16O2	155.10775	155.10788	0.8	1.8E+04
55	3-Mercaptolactate	[M+Cl]-	C3H6O3S	156.97317	156.97291	-1.6	1.4E+04
56	Dihydropteridine	[M+Na]+	C6H6N4	157.04847	157.04867	1.3	7.3E+04
57	Oxoadipic acid	[M+H]+	C6H8O5	161.04445	161.04401	-2.7	4.4E+04
58	Aminoadipic acid	[M+H]+	C6H11NO4	162.07608	162.07576	-2.0	5.4E+04
59	Tetrahydropyridine-2-carboxylate	[M+K]+	C6H9NO2	166.02649	166.02695	2.8	6.4E+04
60	L-Phenylalanine	[M+H]+	C9H11NO2	166.08626	166.08609	-1.0	9.6E+04
61	Phosphonoalanine	[M-H]-	C3H8NO5P	168.00673	168.00698	1.5	1.4E+04
62	L-Aspartate	[M+Cl]-	C4H7NO4	168.00691	168.00698	0.4	1.4E+04
63	Agmatine	[M+K]+	C5H14N4	169.08501	169.08531	1.8	4.8E+04
64	Cyclo(Ala-Val)	[M+H]+	C8H12N2O2	169.09715	169.09761	2.7	5.9E+04
65	Acetylagmatine	[M-H]-	C7H16N4O	171.12513	171.12489	-1.4	2.4E+04
66	(-)-Carvone	[M+Na]+	C10H14O	173.09369	173.09347	-1.2	5.8E+04
67	2-Nitrophenol	[M+Cl]-	C6H5NO3	173.99634	173.99650	0.9	1.2E+04
68	2-Quinolinecarboxylic acid	[M+H]+	C10H7NO2	174.05495	174.05531	2.0	4.4E+04
69	1,3-Dimethyluracil	[M+Cl]-	C6H8N2O2	175.02798	175.02763	-2.0	1.7E+04
70	L-Arginine	[M+H]+	C6H14N4O2	175.11895	175.11895	0.0	3.7E+05
71	cis-Carboxymethylenebutenolide	[M+K]+	C6H4O4	178.97412	178.97377	-1.9	5.0E+04
72	2-Hydroxypropylphosphonate	[M+K]+	C3H9O4P	178.98700	178.98657	-2.4	6.5E+04
73	Aldohexose	[M-H]-	C6H12O6	179.05611	179.05568	-2.4	2.4E+05
74	Eugenol methyl ether	[M+H]+	C11H14O2	179.10666	179.10628	-2.1	5.4E+04
75	Carbamoyl phosphate	[M+K]+	CH4NO5P	179.94587	179.94633	2.6	4.8E+04

76	Caffeic acid	[M+H]+	C9H8O4	181.04954	181.04954	0.0	1.1E+05
77	L-Lathyrine	[M-H]-	C7H10N4O2	181.07310	181.07345	1.9	7.0E+04
78	5-Hydroxyisourate	[M-H]-	C5H4N4O4	183.01598	183.01562	-2.0	1.4E+04
79	3-Sulfinoalanine	[M+Cl]-	C3H6NO4S	186.97116	186.97153	2.0	1.3E+04
80	2-Amino-4-nitrotoluene	[M+Cl]-	C7H8N2O2	187.02798	187.02771	-1.4	1.3E+04
81	Undecanoic acid	[M+H]+	C11H22O2	187.16926	187.16924	-0.1	5.1E+04
82	Triethanolamine	[M+K]+	C6H15NO3	188.06835	188.06836	0.0	1.9E+05
83	Phosphoenolpyruvate	[M+Na]+	C3H5O6P	190.97160	190.97147	-0.7	7.6E+04
84	Citrate	[M-H]-	C6H8O7	191.01973	191.01985	0.6	1.8E+04
85	4-Nitroquinoline N-oxide	[M+H]+	C9H6N2O3	191.04512	191.04533	1.1	5.3E+04
86	2-Oxo-7-methylthioheptanoic acid	[M+H]+	C8H14O3S	191.07364	191.07390	1.4	5.6E+04
87	4-Aminohippuric acid	[M-H]-	C9H10N2O3	193.06187	193.06205	1.0	1.9E+04
88	Nonanoic acid	[M+Cl]-	C9H18O2	193.10008	193.10024	0.8	1.6E+04
89	L-Histidine	[M+K]+	C6H9N3O2	194.03264	194.03235	-1.5	5.2E+04
90	Indoleacetaldehyde	[M+Cl]-	C10H9NO	194.03782	194.03819	1.9	1.3E+04
91	Benzenamine sulfate	[M+Na]+	C6H6NO3S	194.99606	194.99601	-0.3	6.3E+04
92	1,6-Dimethylnaphthalene	[M+K]+	C12H12	195.05706	195.05670	-1.8	6.9E+04
93	(S)-Carnitine	[M+Cl]-	C7H15NO3	196.07459	196.07489	1.5	2.3E+04
94	N-Acetylornithine	[M+Na]+	C7H14N2O3	197.08966	197.08912	-2.8	7.6E+04
95	Acetylcysteine	[M+Cl]-	C5H9NO3S	197.99972	197.99951	-1.0	1.6E+04
96	Hydroxyatrazine	[M+H]+	C8H15N5O	198.13494	198.13469	-1.2	5.8E+04
97	4,5-Dihydroxyphthalate	[M+H]+	C8H6O6	199.02371	199.02327	-2.2	1.1E+05
98	1,5-Anhydro-D-glucitol	[M+Cl]-	C6H12O5	199.03788	199.03760	-1.4	1.4E+04
99	5-Acetylamino-6-amino-3-methyluracil	[M+H]+	C7H10N4O3	199.08257	199.08244	-0.6	8.7E+04
100	L-Methionine (R)-S-oxide	[M+Cl]-	C5H11NO3S	200.01537	200.01554	0.9	1.4E+04
101	Eugenol methyl ether	[M+Na]+	C11H14O2	201.08860	201.08857	-0.2	1.0E+05
102	4-Aminophenyl ether	[M+H]+	C12H12N2O	201.10224	201.10201	-1.1	6.0E+04
103	Diethyl adipate	[M-H]-	C10H18O4	201.11323	201.11346	1.1	3.3E+04
104	7-Aminomethyl-7-carbaguanine	[M+Na]+	C7H9N5O	202.06993	202.07022	1.4	7.0E+04
105	(5-Phenyl-1,2,4-triazol-3-yl)urea	[M-H]-	C9H9N5O	202.07343	202.07384	2.0	1.3E+04
106	D-Aldose	[M+Na]+	C6H12O6	203.05261	203.05260	0.0	7.1E+05
107	L-Phenylalanine	[M+K]+	C9H11NO2	204.04214	204.04256	2.1	1.8E+05
108	Propylthiouracil	[M+Cl]-	C7H10N2OS	205.02079	205.02018	-3.0	1.7E+04
109	Sorbitol	[M+Na]+	C6H14O6	205.06826	205.06843	0.8	2.3E+06
110	4-Octylphenol	[M-H]-	C14H22O	205.15979	205.15946	-1.6	2.8E+04
111	9-oxo-2E-decenoic acid	[M+Na]+	C10H16O3	207.09917	207.09975	2.8	7.7E+04
112	Dodecylaldehyde	[M+Na]+	C12H24O	207.17194	207.17214	1.0	6.0E+04
113	Cysteic acid	[M+K]+	C3H7NO5S	207.96765	207.96735	-1.5	9.9E+04
114	(S)-2-O-Sulfolactate	[M+K]+	C3H6O6S	208.95167	208.95149	-0.9	1.0E+05

115	Propanoylagmatine	[M+Na]+	C8H18N4O	209.13728	209.13680	-2.3	1.2E+05
116	N4-Phosphoagmatine	[M+H]+	C5H15N4O3P	211.09545	211.09579	1.6	9.1E+04
117	2-tridecene-4,7-diynal	[M+Na]+	C13H16O	211.10934	211.10988	2.6	9.7E+04
118	4-Hydroxylamino-2,6-dinitrotoluene	[M+H]+	C7H7N3O5	214.04585	214.04600	0.7	7.1E+04
119	Bergapten	[M-H]-	C12H8O4	215.03498	215.03558	2.8	4.1E+04
120	Adrenochrome o-semiquinone	[M+Cl]-	C9H10NO3	215.03547	215.03558	0.5	4.1E+04
121	L-Lathyrine	[M+Cl]-	C7H10N4O2	217.04978	217.04969	-0.4	2.0E+05
122	2,2',3-Trihydroxydiphenylether	[M-H]-	C12H10O4	217.05063	217.05062	-0.1	3.5E+05
123	5-O-Methyl-myo-inositol	[M+Na]+	C7H14O6	217.06826	217.06800	-1.2	7.1E+04
124	3-(3,4-Dihydroxyphenyl)pyruvate	[M+Na]+	C9H8O5	219.02639	219.02656	0.8	1.0E+06
125	2-tridecenal	[M+Na]+	C13H24O	219.17194	219.17140	-2.4	7.1E+04
126	3-(3,4-Dihydroxyphenyl)lactate	[M+Na]+	C9H10O5	221.04204	221.04229	1.1	2.3E+06
127	6-Acetyl-D-glucose	[M-H]-	C8H14O7	221.06668	221.06686	0.8	2.7E+04
128	7-Methyl-2-hydroxy-6-oxoocta-2,4-dienoate	[M+K]+	C9H12O4	223.03672	223.03685	0.6	1.8E+05
129	Diethyl phthalate	[M+H]+	C12H14O4	223.09649	223.09591	-2.6	3.5E+05
130	O-Phospho-L-serine	[M+K]+	C3H8NO6P	223.97208	223.97192	-0.7	1.0E+05
131	Cytisine	[M+Cl]-	C11H14N2O	225.08001	225.08012	0.5	2.1E+04
132	L-Glutamic acid 5-phosphate	[M-H]-	C5H10NO7P	226.01221	226.01236	0.7	2.2E+04
133	Deoxycytidine	[M-H]-	C9H13N3O4	226.08333	226.08268	-2.9	2.5E+04
134	L-Tryptophan	[M+Na]+	C11H12N2O2	227.07910	227.07963	2.3	9.4E+04
135	Myristic acid	[M-H]-	C14H28O2	227.20165	227.20130	-1.6	1.1E+05
136	Prolylhydroxyproline	[M+H]+	C10H16N2O4	229.11828	229.11779	-2.2	1.2E+05
137	Isovalerylglutamic acid	[M+H]+	C10H17NO5	232.11795	232.11729	-2.8	1.3E+05
138	Gamma-Aminobutyryl-lysine	[M+H]+	C10H21N3O3	232.16557	232.16623	2.9	1.6E+05
139	N-Acetylgalactosaminate	[M-H]-	C8H13NO7	234.06193	234.06176	-0.7	1.6E+04
140	Nitro-L-arginine methyl ester	[M+H]+	C7H15N5O4	234.11968	234.11962	-0.3	8.9E+04
141	D-Erythrose 4-phosphate	[M+Cl]-	C4H9O7P	234.97799	234.97830	1.3	3.1E+04
142	3,3'-Dimethylbenzidine	[M+Na]+	C14H16N2	235.12057	235.12040	-0.7	2.9E+05
143	11E-Tetradecen-1-ol	[M+Na]+	C14H28O	235.20324	235.20257	-2.8	8.1E+04
144	4-Sulfobenzoate	[M+Cl]-	C7H6O5S	236.96300	236.96256	-1.8	2.2E+04
145	N-Formyl-D-kynurenine	[M+H]+	C11H12N2O4	237.08698	237.08705	0.3	1.2E+05
146	10-hydroxy-undecanoic acid	[M+Cl]-	C11H22O3	237.12630	237.12605	-1.0	2.2E+04
147	Triethylenemelamine	[M+Cl]-	C9H12N6	239.08175	239.08202	1.1	2.6E+04
148	Thymidine	[M-H]-	C10H14N2O5	241.08300	241.08269	-1.3	1.7E+05
149	Pentadecanoic acid	[M-H]-	C15H30O2	241.21730	241.21736	0.2	1.5E+05
150	Dimethylallyl diphosphate	[M-H]-	C5H12O7P2	244.99855	244.99785	-2.9	2.7E+04
151	2,6-Dihydroxypseudooxynicotine	[M+Cl]-	C10H14N2O3	245.06984	245.06948	-1.5	2.9E+04
152	*p*-Methoxystilbene	[M+Cl]-	C15H14O	245.07387	245.07331	-2.3	3.1E+04
153	Monobutylphthalate	[M+Na]+	C12H14O4	245.07843	245.07856	0.5	6.2E+05

154	Heptadecadiene-4,6-diyn-3-ol	[M+H]+	C17H24O	245.18999	245.19015	0.6	1.3E+05
155	Cyclic 2,3-bisphospho-D-glycerate	[M-H]-	C3H6O9P2	246.94143	246.94175	1.3	4.5E+04
156	N-Acetyl-D-tryptophan	[M+H]+	C13H14N2O3	247.10772	247.10807	1.4	1.4E+05
157	1,4'-Bipiperidine-1'-carboxylic acid	[M+Cl]-	C11H20N2O2	247.12188	247.12244	2.3	3.1E+04
158	3,7,11-Trimethyl-6E,10-dodecadien-1-ol	[M+Na]+	C15H28O	247.20324	247.20287	-1.5	9.0E+04
159	1-Nitropyrene	[M+H]+	C16H9NO2	248.07060	248.07067	0.3	1.4E+05
160	2-Hydroxy-6-oxo-6-(2-hydroxyphenoxy)-hexa-2,4-dienoate	[M-H]-	C12H10O6	249.04046	249.04106	2.4	3.5E+04
161	6Z,9Z,12Z-hexadecatrienoic acid	[M-H]-	C16H26O2	249.18600	249.18549	-2.1	2.4E+04
162	L-Serine-phosphoethanolamine	[M+Na]+	C5H13N2O6P	251.04034	251.04083	1.9	1.5E+05
163	2,4,6-Trihydroxybenzophenone	[M+Na]+	C13H10O4	253.04713	253.04682	-1.2	2.6E+05
164	Dihydroxybibenzyl	[M+K]+	C14H14O2	253.06254	253.06276	0.9	1.6E+05
165	D-Lysopine	[M+Cl]-	C9H18N2O4	253.09606	253.09580	-1.0	4.7E+04
166	Pantothenic acid	[M+Cl]-	C9H17NO5	254.08007	254.07986	-0.8	3.0E+04
167	N-Acetyl-b-glucosaminylamine	[M+Cl]-	C8H16N2O5	255.07532	255.07564	1.2	5.4E+04
168	Palmitic acid	[M-H]-	C16H32O2	255.23295	255.23238	-2.2	3.3E+05
169	Hydroxypropionylcarnitine	[M+Na]+	C10H19NO5	256.11554	256.11548	-0.2	5.5E+05
170	2'-Hydroxyisoflavone	[M+Na]+	C15H10O3	261.05221	261.05238	0.6	5.6E+05
171	3,4-Diphenyltetrahydrofuran	[M+K]+	C16H16O	263.08327	263.08281	-1.8	1.3E+05
172	2Z-Dodecenedioic acid	[M+Cl]-	C12H20O4	263.10556	263.10518	-1.4	3.8E+04
173	N2-Succinyl-L-ornithine	[M+Cl]-	C9H16N2O5	267.07532	267.07556	0.9	1.5E+05
174	Tributyl phosphate	[M+H]+	C12H27O4P	267.17197	267.17181	-0.6	2.0E+05
175	Dihydroresveratrol	[M+K]+	C14H14O3	269.05745	269.05712	-1.2	1.4E+05
176	Doxylamine	[M-H]-	C17H22N2O	269.16594	269.16566	-1.0	1.1E+05
177	Hydroxypropionylcarnitine	[M+K]+	C10H19NO5	272.08948	272.08978	1.1	3.3E+05
178	N2-Succinyl-L-arginine	[M-H]-	C10H18N4O5	273.12044	273.11983	-2.2	4.2E+04
179	3,6,9,12,15-octadecapentaenoic acid	[M+H]+	C18H26O2	275.20056	275.19976	-2.9	2.3E+05
180	3-Oxosteroid	[M+H]+	C19H30O	275.23694	275.23660	-1.2	2.3E+05
181	Hydroxyhexanoycarnitine	[M+H]+	C13H25NO5	276.18055	276.18121	2.4	5.6E+05
182	Glu-Glu	[M+H]+	C10H16N2O7	277.10303	277.10308	0.2	3.3E+05
183	Palmitoleic acid	[M+Na]+	C16H30O2	277.21380	277.21330	-1.8	3.2E+05
184	2'-Hydroxy-4'-methoxydihydrochalcone	[M+Na]+	C16H16O3	279.09917	279.09864	-1.9	4.1E+05
185	1,11-Undecanedicarboxylic acid	[M+Cl]-	C13H24O4	279.13686	279.13616	-2.5	5.5E+04
186	Dibutyl phthalate	[M+H]+	C16H22O4	279.15909	279.15916	0.3	4.0E+06
187	5,8,11-heptadecatriynoic acid	[M+Na]+	C17H22O2	281.15120	281.15138	0.6	1.7E+05
188	all-trans-Dehydroretinal	[M+H]+	C20H26O	283.20564	283.20546	-0.6	7.4E+05
189	12S-hydroxy-16-heptadecynoic acid	[M+H]+	C17H30O3	283.22677	283.22677	0.0	4.3E+05
190	Retinol	[M+H]+	C20H30O	287.23694	287.23759	2.3	4.5E+06
191	L-Hyoscyamine	[M-H]-	C17H23NO3	288.16052	288.16105	1.8	4.3E+04
192	2,7-Anhydro-alpha-N-acetylneuraminic acid	[M-H]-	C11H17NO8	290.08814	290.08858	1.5	1.5E+05

193	Androsterone	[M+H]+	C19H30O2	291.23186	291.23245	2.0	9.0E+05
194	17-Methyl-18-norandrosta-4,13(17)-dien-3-one	[M+Na]+	C19H26O	293.18759	293.18760	0.0	2.4E+05
195	16alpha-Hydroxysteroid	[M+H]+	C19H32O2	293.24751	293.24759	0.3	8.5E+05
196	Tridecanoylglycine	[M+Na]+	C15H29NO3	294.20396	294.20314	-2.8	1.7E+05
197	Aspartame	[M+H]+	C14H18N2O5	295.12885	295.12949	2.2	2.5E+05
198	N(6)-(Octanoyl)lysine	[M+Na]+	C14H28N2O3	295.19921	295.19896	-0.9	2.2E+05
199	Aminoimidazole ribotide	[M+H]+	C8H14N3O7P	296.06421	296.06375	-1.6	2.2E+05
200	3,6,9,12,15-octadecapentaenoic acid	[M+Na]+	C18H26O2	297.18250	297.18313	2.1	5.0E+05
201	8-Hydroxylinoleic acid	[M+H]+	C18H32O3	297.24242	297.24156	-2.9	1.8E+06
202	7-Mercaptoheptanoylthreonine	[M+Cl]-	C11H21NO4S	298.08853	298.08767	-2.9	8.3E+04
203	Hydroxyhexanoycarnitine	[M+Na]+	C13H25NO5	298.16249	298.16282	1.1	2.6E+06
204	Pentadecanoylglycine	[M-H]-	C17H33NO3	298.23877	298.23954	2.6	4.1E+04
205	Benzoyl glucuronide	[M+H]+	C13H14O8	299.07614	299.07672	1.9	3.2E+05
206	3,11-dihydroxy myristoic acid	[M+K]+	C14H28O4	299.16192	299.16224	1.1	7.8E+05
207	4-Oxoretinal	[M+H]+	C20H26O2	299.20056	299.20135	2.7	1.0E+06
208	Trihexyphenidyl	[M-H]-	C20H31NO	300.23329	300.23405	2.5	5.4E+04
209	Dibutyl phthalate	[M+Na]+	C16H22O4	301.14103	301.14128	0.8	5.4E+06
210	3-Deoxy-D-glycero-D-galacto-2-nonulosonic acid	[M+Cl]-	C9H16O9	303.04883	303.04966	2.7	4.5E+04
211	Inosine	[M+Cl]-	C10H12N4O5	303.05017	303.04966	-1.7	4.5E+04
212	Evoxanthidine	[M+Cl]-	C15H11NO4	304.03821	304.03901	2.6	3.9E+04
213	4-(3-Methylbut-2-enyl)-L-tryptophan	[M+K]+	C16H20N2O2	311.11564	311.11483	-2.6	3.5E+05
214	Methyl 6-(4-isopropyl-4-methyl-5-oxo-2-imidazolin-2-yl)-m-toluate	[M+Na]+	C16H20N2O3	311.13661	311.13615	-1.5	4.8E+05
215	16-Dehydroprogesterone	[M+H]+	C21H28O2	313.21621	313.21538	-2.6	3.1E+06
216	16,17-Didehydropregnenolone	[M+H]+	C21H30O2	315.23186	315.23163	-0.7	1.1E+06
217	2-Ethylidene-1,5-dimethyl-3,3-diphenylpyrrolidine	[M+K]+	C20H23N	316.14621	316.14707	2.7	2.8E+05
218	Oleic acid	[M+Cl]-	C18H33O2	316.21746	316.21711	-1.1	3.9E+04
219	2-Methylaminoadenosine	[M+Na]+	C11H16N6O4	319.11252	319.11213	-1.2	3.9E+05
220	Leukotriene A4	[M+H]+	C20H30O3	319.22677	319.22655	-0.7	1.4E+06
221	Deoxy-5-methylcytidylate	[M-H]-	C10H16N3O7P	320.06531	320.06580	1.5	4.2E+04
222	N4-Acetylcytidine	[M+Cl]-	C11H15N3O6	320.06549	320.06580	1.0	4.2E+04
223	3-Epihydroxy-2'-deoxymugineic acid	[M+H]+	C12H20N2O8	321.12924	321.12884	-1.3	5.5E+05
224	11-cis-Dehydroretinal	[M+K]+	C20H26O	321.16152	321.16202	1.5	2.5E+05
225	(8)-Gingerol	[M-H]-	C19H30O4	321.20713	321.20770	1.8	3.3E+05
226	Linolenoyl ethanolamide	[M+H]+	C20H35NO2	322.27406	322.27501	3.0	5.1E+06
227	Retinal	[M+K]+	C20H28O	323.17717	323.17661	-1.7	7.2E+05
228	4-(3,5-Diphenylcyclohexyl)phenol	[M-H]-	C24H24O	327.17544	327.17593	1.5	3.3E+05
229	Methylpregna-4,9(11)-diene-3,20-dione	[M+H]+	C22H30O2	327.23186	327.23174	-0.4	1.9E+07
230	12-Keto-leukotriene B4	[M-H]-	C20H30O4	333.20713	333.20640	-2.2	7.7E+04
231	10-keto stearic acid	[M+Cl]-	C18H34O3	333.22020	333.21964	-1.7	9.5E+04

232	17-Ethynyl-5alpha-androstan-17beta-ol	[M+Cl]-	C21H32O	335.21472	335.21563	2.7	8.8E+04
233	1-Guanidino-1-deoxy-scyllo-inositol 4-phosphate	[M+Cl]-	C7H16N3O8P	336.03690	336.03678	-0.4	1.4E+05
234	(Ac)2-L-Lys-D-Ala	[M+Cl]-	C13H23N3O5	336.13317	336.13299	-0.5	5.1E+04
235	17-Ethynyl-10-hydroxy-19-nortestosterone	[M+Na]+	C20H26O3	337.17742	337.17711	-0.9	1.0E+06
236	Prostaglandin B1	[M+H]+	C20H32O4	337.23734	337.23708	-0.8	1.2E+06
237	11R-HEPE	[M+Na]+	C20H29O3	340.20089	340.20155	1.9	4.3E+05
238	17beta-Nitro-5alpha-androstane	[M+Cl]-	C19H31NO2	340.20488	340.20529	1.2	4.3E+05
239	3-Hydroxyquinine	[M+H]+	C20H24N2O3	341.18597	341.18611	0.4	5.3E+06
240	10-HETE	[M+Na]+	C20H31O3	342.21654	342.21667	0.4	1.1E+06
241	Arachidonic acid	[M+K]+	C20H32O2	343.20339	343.20424	2.5	2.5E+07
242	Glutathione	[M+K]+	C10H17N3O6S	346.04697	346.04732	1.0	2.2E+05
243	Affinisine	[M+K]+	C20H24N2O	347.15202	347.15116	-2.5	3.0E+05
244	3a,21-Dihydroxy-5b-pregnane-11,20-dione	[M-H]-	C21H32O4	347.22278	347.22196	-2.4	5.9E+04
245	(-)-Chimonanthine	[M+H]+	C22H26N4	347.22302	347.22374	2.1	5.1E+05
246	C25:1 Monocyclic highly branched isoprenoid	[M-H]-	C25H48	347.36833	347.36785	-1.4	9.0E+04
247	Arotinoid acid	[M+H]+	C24H28O2	349.21621	349.21579	-1.2	2.1E+05
248	19-hydroxy-nonadecanoic acid	[M+Cl]-	C19H38O3	349.25150	349.25101	-1.4	5.2E+04
249	N-Glycolyl-D-mannosamine 6-phosphate	[M+Cl]-	C8H16NO10P	352.02058	352.02136	2.2	1.1E+05
250	Gibberellin A12 aldehyde	[M+K]+	C20H28O3	355.16700	355.16653	-1.3	1.5E+06
251	(+-)-Trihydroxy-decipiadiene	[M+Cl]-	C20H32O3	355.20455	355.20397	-1.6	3.7E+05
252	cyclo-Dopa 5-O-glucoside	[M-H]-	C15H19NO9	356.09870	356.09804	-1.9	4.5E+04
253	Leukotriene A4	[M+K]+	C20H30O3	357.18265	357.18273	0.2	4.8E+06
254	Streptobiosamine	[M+Na]+	C13H23NO9	360.12650	360.12640	-0.3	3.3E+05
255	Benzyl viologen	[M+Na]+	C24H22N2	361.16752	361.16769	0.5	5.3E+05
256	(Hydroxymethyl)-5alpha-androstane-diol	[M+K]+	C20H34O3	361.21395	361.21490	2.6	1.6E+06
257	Hydroxy-methyl-androstan-3-one propionate	[M+H]+	C23H36O3	361.27372	361.27358	-0.4	4.4E+05
258	Decylubiquinol	[M+K]+	C19H32O4	363.19322	363.19420	2.7	2.3E+05
259	Docosahexaenoic acid	[M+Cl]-	C22H32O2	363.20963	363.21008	1.2	5.8E+04
260	Sucrose	[M+Na]+	C12H22O11	365.10543	365.10646	2.8	1.0E+06
261	2-hexadecanoyl-glycerol	[M+Cl]-	C19H38O4	365.24641	365.24571	-1.9	1.3E+05
262	Methyl-eicosanoic acid	[M+K]+	C21H42O2	365.28164	365.28079	-2.3	1.5E+06
263	Tetramethoxychalcone	[M+K]+	C19H20O5	367.09423	367.09419	-0.1	2.2E+05
264	Dicyclohexyl phthalate	[M+K]+	C20H26O4	369.14627	369.14545	-2.2	3.0E+05
265	Riboflavin reduced	[M+Na]+	C15H16N4O6	371.09620	371.09564	-1.5	2.9E+05
266	Nicotine glucuronide	[M+Cl]-	C16H22N2O6	373.11719	373.11661	-1.5	5.4E+04
267	Erucic acid	[M+Cl]-	C22H42O2	373.28788	373.28715	-2.0	5.8E+04
268	Spinochalcone B	[M+H]+	C25H26O3	375.19547	375.19453	-2.5	1.2E+06
269	alpha-Ribazole 5'-phosphate	[M+Na]+	C14H19N2O7P	381.08221	381.08116	-2.8	6.3E+05
270	Succinyladenosine	[M-H]-	C14H17N5O8	382.10044	382.09931	-2.9	1.6E+05

271	13,14-Dihydro-15-oxo-lipoxin A4	[M+Cl]-	C20H31O5	386.18655	386.18682	0.7	5.1E+04
272	Leukotriene B4 dimethylamide	[M+Na]+	C22H37NO3	386.26656	386.26548	-2.8	2.7E+05
273	N-arachidonoyl GABA	[M-H]-	C24H39NO3	388.28572	388.28650	2.0	4.2E+04
274	Paratocarpin A	[M+H]+	C25H24O4	389.17474	389.17390	-2.1	5.5E+05
275	5-Methoxy-7,8-diprenylflavone	[M+H]+	C26H28O3	389.21112	389.21202	2.3	3.2E+05
276	6-Butyryl-5-hydroxy-4-phenylseselin	[M+H]+	C24H22O5	391.15400	391.15448	1.2	3.0E+05
277	Licoflavone B	[M+H]+	C25H26O4	391.19039	391.18951	-2.2	4.7E+05
278	3-Oxochola-1,4,6-trien-24-oic Acid	[M+Na]+	C24H32O3	391.22437	391.22551	2.9	3.2E+05
279	2,2-Dimethyl-3-(4-methoxyphenyl)-4-ethyl-8-(1-pyrrolidinylmethyl)-2H-1-benzopyran-7-ol	[M-H]-	C25H31NO3	392.22312	392.22209	-2.6	5.6E+04
280	N-stearoyl serine	[M+Na]+	C21H41NO4	394.29278	394.29349	1.8	3.4E+05
281	gibberellin A28	[M+H]+	C20H26O8	395.17004	395.17114	2.8	5.0E+05
282	Tris(butoxyethyl)phosphate	[M-H]-	C18H39O7P	397.23606	397.23503	-2.6	2.1E+06
283	19-norcholestanol	[M+Na]+	C26H46O	397.34409	397.34412	0.1	2.3E+05
284	N-Benzoyl-D-arginine-4-nitroanilide	[M+H]+	C19H22N6O4	399.17753	399.17716	-0.9	2.6E+05
285	1-(1,2,3,4,5-pentahydroxypent-1-yl)-1,2,3,4-tetrahydro-beta-carboline-3-carboxylate	[M+Cl]-	C17H21N2O7	400.10428	400.10452	0.6	4.8E+04
286	Estradiol-17-phenylpropionate	[M-H]-	C27H32O3	403.22787	403.22794	0.2	9.1E+04
287	(-)-epicatechin sulfate	[M+Cl]-	C15H13O9S	403.99743	403.99822	2.0	1.4E+05
288	PA(14:0/0:0)	[M+Na]+	C17H35O7P	405.20126	405.20240	2.8	3.2E+05
289	Epicatechin 3-O-p-hydroxybenzoate	[M-H]-	C22H18O8	409.09289	409.09323	0.8	7.2E+04
290	Sterol 3-beta-D-glucoside	[M-H]-	C23H38O6	409.25956	409.25967	0.3	9.3E+04
291	19-norcholestenone	[M+K]+	C26H42O	409.28673	409.28556	-2.8	4.7E+05
292	Myxalamid A	[M+H]+	C26H41NO3	416.31592	416.31501	-2.2	4.2E+05
293	Dihydroxy-pregnan-one diacetate	[M+H]+	C25H38O5	419.27920	419.28045	3.0	2.0E+05
294	Vitamin D2	[M+Na]+	C28H44O	419.32844	419.32947	2.5	2.0E+05
295	Propylestra-triene-diol diacetate	[M+Na]+	C25H34O4	421.23493	421.23560	1.6	5.2E+05
296	Cholesterol	[M+Cl]-	C27H46O	421.32427	421.32510	2.0	6.5E+04
297	2'-Hydroxy-3,5,7,4',5'-pentamethoxyflavone	[M+Cl]-	C20H20O8	423.08522	423.08574	1.2	4.8E+04
298	1-Octen-3-ol-3-o-beta-D-xylopyranosyl(1->6)-beta-D-glucopyranoside	[M+H]+	C19H34O10	423.22247	423.22251	0.1	3.6E+05
299	Cycloprotobuxine C	[M+Na]+	C27H48N2	423.37097	423.37005	-2.2	6.1E+05
300	PGE2-dihydroxypropanylamine	[M-H]-	C23H39NO6	424.27046	424.27036	-0.2	7.9E+04
301	Inosine 2'-phosphate	[M-H]-	C10H14N4O11P2	427.00615	427.00735	2.8	6.1E+04
302	Dihydroxy-norvitamin D3	[M+Na]+	C26H44O3	427.31827	427.31919	2.2	2.8E+05
303	Anandamide 0-phosphate	[M+H]+	C22H38NO5P	428.25604	428.25637	0.8	1.8E+05
304	trans-Zeatin riboside monophosphate	[M-H]-	C15H22N5O8P	430.11332	430.11257	-1.8	9.2E+04
305	Veratramine	[M+Na]+	C27H39NO2	432.28730	432.28751	0.5	8.3E+05
306	22-methyl-5Z,9Z-octacosadienoic acid	[M-H]-	C29H54O2	433.40510	433.40397	-2.6	5.5E+04
307	MG(0:0/22:1(13Z)/0:0)	[M+Na]+	C25H48O4	435.34448	435.34565	2.7	2.8E+05

308	Deacetylvindoline	[M+Na]+	C23H30N2O5	437.20469	437.20571	2.3	3.2E+05
309	Cyclopropyl-21-nor-9,10-seco-cholestatetraene-1,3,25-triol	[M-H]-	C29H44O3	439.32177	439.32177	0.0	7.7E+04
310	N-(2-phenoxy-ethyl) arachidonoyl amine	[M+Na]+	C28H41NO2	446.30295	446.30288	-0.2	4.4E+05
311	alpha-Phocaecholic acid	[M+Na]+	C24H40O6	447.27171	447.27282	2.5	2.9E+05
312	(S)-3-hydroxyhexacosanoic acid	[M+Cl]-	C26H52O3	447.36105	447.36187	1.8	1.1E+05
313	C30:5 Monocyclic highly branched isoprenoid B	[M+K]+	C30H52	451.37006	451.37097	2.0	3.1E+05
314	Solanocapsine	[M+Na]+	C27H46N2O2	453.34515	453.34440	-1.7	4.9E+05
315	1alpha,25-dihydroxy-3-deoxy-3-thiavitamin D3 3-oxide	[M+Na]+	C26H42O3S	457.27469	457.27427	-0.9	5.6E+05
316	2alpha-(3-Hydroxypropyl)-1alpha,25-dihydroxy-19-norvitamin D3	[M-H]-	C29H50O4	461.36363	461.36408	1.0	7.8E+04
317	Z-Arg-Arg	[M+H]+	C20H32N8O5	465.25684	465.25801	2.5	8.7E+05
318	Sphingosine-1-phosphocholine	[M+H]+	C23H49N2O5P	465.34519	465.34518	0.0	5.5E+05
319	Obliquine	[M+Na]+	C26H28N2O5	471.18904	471.18770	-2.8	2.7E+05
320	11-trans-LTE4	[M+Cl]-	C23H37NO5S	474.20865	474.20868	0.1	9.0E+04
321	Docosa-4,7,10,13,16-pentaenoyl carnitine	[M+H]+	C29H47NO4	474.35779	474.35646	-2.8	3.7E+05
322	Palmityl oleate	[M-H]-	C32H62O2	477.46770	477.46711	-1.2	6.9E+04
323	CDP-glycerol	[M+H]+	C12H21N3O13P2	478.06224	478.06114	-2.3	2.5E+05
324	Ecalcidene	[M+Na]+	C29H45NO3	478.32917	478.33048	2.7	4.4E+05
325	3-Sulfodeoxycholic acid	[M+Na]+	C23H38O7S	481.22305	481.22251	-1.1	5.9E+05
326	13'-hydroxy-alpha-tocopherol	[M+Cl]-	C29H50O3	481.34540	481.34459	-1.7	1.0E+05
327	CTP	[M-H]-	C9H16N3O14P3	481.97724	481.97833	2.3	6.0E+04
328	4-Deoxy-beta-D-gluc-4-enuronosyl-(1,3)-N-acetyl-D-galactosamine-sulfate	[M+Na]+	C14H21NO14S	482.05750	482.05880	2.7	4.2E+05
329	sn-3-O-(Geranylgeranyl)glycerol 1-phosphate	[M+K]+	C23H41O6P	483.22724	483.22670	-1.1	9.0E+05
330	Galactosylsphingosine	[M+Na]+	C24H47NO7	484.32447	484.32337	-2.3	3.1E+05
331	1-(1Z-eicosenyl)-glycero-3-phosphate	[M+Cl]-	C23H47O6P	485.28043	485.28036	-0.1	5.5E+05
332	7-Dehydrocholesterol benzoate	[M-H]-	C34H48O2	487.35815	487.35670	-3.0	1.3E+05
333	Diethyl-1alpha,25-dihydroxy-20,21-methano-23-oxavitamin D3	[M+H]+	C31H50O4	487.37819	487.37901	1.7	3.6E+05
334	1-(5'-Phosphoribosyl)-5-amino-4-(N-succinocarboxamide)-imidazole	[M+Cl]-	C13H19N4O12P	489.04311	489.04256	-1.1	6.1E+04
335	1alpha-hydroxy-24-(dimethoxyphosphoryl)-25,26,27-trinorvitamin D3	[M+Na]+	C26H43O5P	489.27403	489.27466	1.3	8.8E+05
336	Vitamin K1 epoxide	[M+Na]+	C31H46O3	489.33392	489.33323	-1.4	1.8E+06
337	3-Oxoglycyrrhetinate	[M+Na]+	C30H44O4	491.31318	491.31203	-2.3	7.7E+05
338	PA(20:3(8Z,11Z,14Z)/0:0)	[M+Cl]-	C23H41O7P	495.22839	495.22791	-1.0	7.6E+04
339	Hydroxy-epi-brassinolide	[M+H]+	C28H48O7	497.34728	497.34658	-1.4	5.9E+05
340	1alpha,25-dihydroxy-24a-homo-26,27-dimethyl-22-thiavitamin D3	[M+Na]+	C29H48O3S	499.32164	499.32148	-0.3	4.8E+05
341	Dihydroxy-dimethyl-hexadehydro-dihomovitamin D3	[M+Cl]-	C31H46O3	501.31410	501.31297	-2.2	7.3E+04
342	Lucidine B	[M+Cl]-	C30H49N3O	502.35696	502.35821	2.5	5.7E+04
343	Mucronine A	[M+H]+	C29H38N4O4	507.29658	507.29758	2.0	7.9E+05
344	Diterpenoid EF-D	[M+Cl]-	C27H38O7	509.23116	509.23146	0.6	1.5E+05
345	N-stearoyl tryptophan	[M+K]+	C29H46N2O3	509.31400	509.31475	1.5	1.6E+06

346	6-Hydroxyluteolin 3'-methyl ether 6,7-disulfate	[M+Cl]-	C16H12O13S2	510.94133	510.94097	-0.7	5.9E+04
347	PA(21:4(6Z,9Z,12Z,15Z)/0:0)	[M+Na]+	C24H44NO7P	512.27476	512.27603	2.5	3.7E+05
348	PS(O-18:0/0:0)	[M+H]+	C24H50NO8P	512.33468	512.33529	1.2	5.4E+05
349	Dihydroxy-(hydroxypropoxy)-norvitamin D3	[M+Cl]-	C29H50O5	513.33523	513.33525	0.0	2.1E+05
350	26,27-diethyl-1alpha,25-dihydroxy-22-thiavitamin D3	[M+Na]+	C30H50O3S	513.33729	513.33645	-1.6	4.8E+05
351	11-O-Demethylpradinone II	[M+K]+	C24H16O11	519.03242	519.03150	-1.8	3.4E+05
352	Dolichyl diphosphate	[M+H]+	C25H46O7P2	521.27915	521.28068	2.9	4.0E+05
353	PG(19:0/0:0)	[M+H]+	C25H51O9P	527.33435	527.33367	-1.3	7.4E+05
354	1-docosanoyl-glycero-3-phosphate	[M+Cl]-	C25H51O7P	529.30664	529.30691	0.5	2.7E+05
355	11-trans-LTD4	[M+Cl]-	C25H40N2O6S	531.23011	531.22933	-1.5	1.1E+05
356	Arg-Pro-Lys-Pro	[M+Cl]-	C22H40N8O5	531.28157	531.28167	0.2	8.7E+04
357	1D-myo-Inositol 1,3,4,5-tetrakisphosphate	[M+K]+	C6H16O18P4	538.89187	538.89108	-1.5	3.0E+05
358	LysoPC(18:2(9Z,12Z))	[M+Na]+	C26H50NO7P	542.32171	542.32138	-0.6	3.4E+05
359	11alpha-Hemiglutaryloxy-1,25-dihydroxyvitamin D3	[M-H]-	C33H52O6	543.36911	543.36858	-1.0	8.7E+04
360	PS(17:1/0:0)	[M+K]+	C23H44NO9P	548.23853	548.23736	-2.1	4.6E+05
361	Octadienoyl-deoxyphorbol-acetate	[M+K]+	C30H40O7	551.24056	551.23901	-2.8	3.8E+05
362	Phoslactomycin B	[M+K]+	C25H40NO8P	552.21231	552.21161	-1.3	3.1E+05
363	11-Hydroxyiridodial glucoside pentaacetate	[M-H]-	C26H36O13	555.20831	555.20848	0.3	1.4E+05
364	Hypercalin B	[M+K]+	C33H42O5	557.26638	557.26505	-2.4	3.1E+05
365	PC(16:0/2:0)	[M+Na]+	C26H52NO8P	560.33227	560.33185	-0.8	3.1E+05
366	11-(4-acetoxymethylphenyl)-1alpha,25-dihydroxy-9,11-didehydrovitamin D3	[M+H]+	C36H50O5	563.37310	563.37373	1.1	2.2E+06
367	31-hydroxy-32,35-anhydrobacteriohopanetetrol	[M+Na]+	C35H60O4	567.43838	567.43796	-0.7	3.5E+05
368	Carboxy-hydroxy-norcholanyl-b-D-Glucopyranosiduronic acid	[M+H]+	C30H48O10	569.33202	569.33214	0.2	4.4E+05
369	Coroglaucigenin-3-o-alpha-L-rhamnopyranoside	[M+Cl]-	C29H44O9	571.26793	571.26902	1.9	7.1E+04
370	Isopentenyladenosine-5'-triphosphate	[M-H]-	C15H24N5O13P3	574.05107	574.05178	1.2	6.7E+04
371	beta-Carotene 5,6-epoxide	[M+Na]+	C40H56O	575.42234	575.42188	-0.8	3.9E+05
372	Coumermic acid	[M+Cl]-	C27H21N3O10	582.09210	582.09118	-1.6	7.1E+04
373	(+)-Myristinin A	[M+Cl]-	C33H40O7	583.24681	583.24624	-1.0	8.1E+04
374	35-aminobacteriohopane-32,33,34-triol	[M+K]+	C35H63NO3	584.44395	584.44283	-1.9	3.7E+05
375	DG(14:0/18:3/0:0)	[M+Na]+	C35H62O5	585.44895	585.44812	-1.4	5.6E+05
376	Cer(d18:2/20:0)	[M-H]-	C38H73NO3	590.55177	590.55237	1.0	6.5E+04
377	14:1 Cholesteryl ester	[M-H]-	C41H70O2	593.53031	593.53097	1.1	1.1E+05
378	stigmast-5-en-3beta-ol 3-0-beta-D-glucopyranoside	[M+Na]+	C35H60O6	599.42821	599.42875	0.9	7.6E+05
379	1-dodecanoyl-2-(9Z,12Z-heptadecadienoyl)-glycero-3-phosphate	[M-H]-	C32H59O8P	601.38748	601.38740	-0.1	1.1E+05
380	1-(4Z,7Z,10Z,13Z,16Z,19Z-docosahexaenoyl)-glycero-3-phosphoserine	[M+Cl]-	C28H44NO9P	604.24477	604.24481	0.1	7.5E+04
381	PS(22:0/0:0)	[M+Na]+	C28H56NO9P	604.35849	604.35875	0.4	4.6E+05
382	Tetrahydrofolyl-[Glu](2)	[M+Cl]-	C24H30N8O9	609.18298	609.18242	-0.9	5.6E+05
383	Ergocristine	[M+H]+	C35H39N5O5	610.30240	610.30173	-1.1	6.5E+05

384	Glutathione disulfide	[M-H]-	C20H32N6O12S2	611.14469	611.14480	0.2	1.9E+05
385	PI(19:1/0:0)	[M+H]+	C28H53O12P	613.33474	613.33522	0.8	6.2E+05
386	1-eicosyl-glycero-3-phospho-(1'-myo-inositol)	[M-H]-	C29H59O11P	613.37222	613.37271	0.8	6.1E+04
387	1-(1Z-hexadecenyl)-2-(9Z-pentadecenoyl)-glycero-3-phosphate	[M-H]-	C34H65O7P	615.43951	615.44043	1.5	1.4E+05
388	3,4-Dihydrospheroidenone	[M+Cl]-	C41H60O2	619.42873	619.42823	-0.8	1.0E+05
389	Squalamine	[M+H]+	C34H65N3O5S	628.47177	628.47238	1.0	1.0E+06
390	DG(14:0/22:6/0:0)	[M+Na]+	C39H64O5	635.46460	635.46520	1.0	9.1E+05
391	PI(19:0/0:0)	[M+Na]+	C28H55O12P	637.33233	637.33311	1.2	6.3E+05
392	Glycocholic acid 3-glucuronide	[M-H]-	C32H53NO12	642.34950	642.34822	-2.0	6.3E+04
393	1-Palmitoyl-2-(5-keto-8-oxo-6-octenoyl)-sn-glycero-3-phosphatidylcholine	[M-H]-	C32H58NO10P	646.37256	646.37121	-2.1	5.6E+04
394	Quercetagetin 4'-methyl ether 7-(6-(E)-caffeylglucoside)	[M-H]-	C31H28O16	655.13046	655.13039	-0.1	9.4E+04
395	PA(O-18:0/14:0)	[M+Na]+	C35H71O7P	657.48296	657.48410	1.7	6.1E+05
396	14-Deacetylnudicauline	[M-H]-	C36H48N2O10	667.32362	667.32304	-0.9	9.9E+04
397	Patuletin 3-(6''-(E)-feruloylglucoside)	[M-H]-	C32H30O16	669.14611	669.14785	2.6	6.7E+04
398	1-octadecanoyl-2-(7Z,10Z,13Z,16Z-docosatetraenoyl)-sn-glycerol	[M-H]-	C43H76O5	671.56200	671.56103	-1.4	6.5E+04
399	Malvidin 3-rutinoside	[M+Cl]-	C29H35O16	674.16191	674.16276	1.3	1.0E+05
400	Patuletin 3-(6''-p-coumaroylglucoside)	[M+Cl]-	C31H28O15	675.11222	675.11301	1.2	7.2E+04
401	N-Acetyl-leu-leu-leu-leu-tyr-amide	[M+H]+	C35H58N6O7	675.44397	675.44251	-2.2	9.1E+05
402	PG(O-16:0/14:1)	[M+H]+	C36H71O9P	679.49085	679.48953	-1.9	1.1E+06
403	PS(O-16:0/13:0)	[M+H]+	C35H70NO9P	680.48610	680.48616	0.1	4.1E+05
404	N-ornithinyl-35-aminobacteriohopane-32,33,34-triol	[M+Na]+	C40H73N3O4	682.54933	682.54815	-1.7	4.6E+05
405	PG(14:1(9Z)/14:1(9Z)	[M+Na]+	C34H63O10P	685.40511	685.40428	-1.2	9.4E+05
406	SM(d18:0/13:0)	[M+Na]+	C36H75N2O6P	685.52550	685.52439	-1.6	5.8E+05
407	Ceramide (d18:1/24:1(15Z)	[M+K]+	C42H81NO3	686.58480	686.58338	-2.1	5.9E+05
408	Alpha-Tetrasaccharide	[M-H]-	C26H45NO20	690.24622	690.24522	-1.4	7.5E+04
409	Hydroxyphthioceranic acid (C46)	[M-H]-	C46H92O3	691.69737	691.69844	1.5	7.8E+04
410	PS(12:0/17:0)	[M+H]+	C35H68NO10P	694.46536	694.46619	1.2	6.9E+05
411	Episteryl oleate	[M+Cl]-	C46H78O2	697.56958	697.56932	-0.4	1.1E+05
412	DG(20:0/22:6/0:0)	[M+H]+	C45H76O5	697.57655	697.57716	0.9	4.6E+05
413	PA(P-20:0/17:2)	[M+H]+	C40H75O7P	699.53232	699.53432	2.9	1.5E+06
414	1-Hexadecanoyl-2-(9Z-octadecenoyl)-sn-glycero-3-phosphonoethanolamine	[M-H]-	C39H76NO7P	700.52866	700.52956	1.3	8.2E+04
415	1-tetradecanyl-2-(8-[3]-ladderane-octanyl)-sn-glycerophosphocholine	[M+H]+	C42H80NO6P	726.57960	726.57859	-1.4	5.5E+05
416	SM(d18:0/16:0)	[M+Na]+	C39H81N2O6P	727.57245	727.57257	0.2	1.1E+06
417	3,5-di-O-(beta-Glucopyranosyl) pelargonidin 6''-O-4, 6'''-O-1-cyclic malate	[M+Cl]-	C31H33O18	728.13609	728.13468	-1.9	7.6E+04
418	PA(P-20:0/19:1)	[M+H]+	C42H81O7P	729.57927	729.57749	-2.4	5.8E+05
419	PG(13:0/20:2)	[M+H]+	C39H73O10P	733.50141	733.49991	-2.0	5.5E+05

420	1-tetradecanyl-2-(8-[3]-ladderane-octanyl)-sn-glycero-3-phospho-(1'-sn-glycerol)	[M+Na]+	C40H75O8P	737.50918	737.51099	2.5	7.9E+05
421	1-Hexadecanoyl-2-(9Z-octadecenoyl)-sn-glycero-3-phosphonoethanolamine	[M+K]+	C39H76NO7P	740.49910	740.49924	0.2	6.0E+05
422	PA(15:0/22:6)	[M+K]+	C40H67O8P	745.42052	745.42211	2.1	4.6E+05
423	DG(22:1/22:6/0:0)	[M+Na]+	C47H78O5	745.57415	745.57223	-2.6	6.4E+05
424	MGDG(18:3/16:3)	[M+H]+	C43H70O10	747.50417	747.50347	-0.9	1.6E+06
425	CE(22:0)	[M+K]+	C49H88O2	747.64159	747.64227	0.9	6.4E+05
426	PE(15:1/22:6)	[M+H]+	C42H70NO8P	748.49118	748.49317	2.7	7.3E+05
427	PA(18:0/22:6)	[M+H]+	C43H73O8P	749.51158	749.51303	1.9	6.7E+05
428	PG(O-16:0/18:3)	[M+Na]+	C40H75O9P	753.50409	753.50317	-1.2	5.7E+05
429	GlcCer(d18:2/20:0)	[M+H]+	C44H83NO8	754.61915	754.61895	-0.3	6.0E+05
430	PC(O-16:0/O-18:0)	[M+Na]+	C42H88NO6P	756.62415	756.62308	-1.4	5.6E+05
431	PG(O-20:0/14:0)	[M+Na]+	C40H81O9P	759.55104	759.55129	0.3	3.0E+06
432	PS(O-18:0/17:2)	[M+H]+	C41H78NO9P	760.54870	760.55007	1.8	1.4E+06
433	1-hexadecyl-2-nonadecanoyl-sn-glycero-3-phosphocholine	[M-H]-	C43H88NO7P	760.62256	760.62199	-0.8	7.8E+04
434	PI(12:0/15:1)	[M+Na]+	C36H67O13P	761.42115	761.42173	0.8	1.1E+06
435	PG(O-16:0/20:2)	[M+H]+	C42H81O9P	761.56910	761.56716	-2.5	8.2E+05
436	OH-Chlorobactene glucoside ester	[M+Cl]-	C47H68O6	763.47099	763.47283	2.4	3.6E+05
437	1-hexadecanoyl-2-(13Z,16Z-docosadienoyl)-glycero-3-phosphate	[M+Cl]-	C41H77O8P	763.50501	763.50368	-1.7	1.8E+05
438	1-(11Z-eicosenoyl)-2-(4Z,7Z,10Z,13Z,16Z,19Z-docosahexaenoyl)-glycero-3-phosphate	[M-H]-	C45H75O8P	773.51268	773.51443	2.3	1.0E+05
439	PS(13:0/22:1)	[M+H]+	C41H78NO10P	776.54361	776.54359	0.0	6.1E+05
440	MGDG(16:0/18:2)	[M+Na]+	C43H78O10	777.54872	777.54656	-2.8	6.4E+05
441	GlcCer(d18:2/22:0)	[M+H]+	C46H87NO8	782.65045	782.64911	-1.7	1.3E+06
442	PI(18:0/12:0)	[M+H]+	C39H75O13P	783.50181	783.50032	-1.9	6.9E+05
443	SM(d18:1/22:1)	[M+H]+	C45H89N2O6P	785.65310	785.65094	-2.8	6.1E+05
444	PE(22:0/P-18:1(11Z))	[M+H]+	C45H88NO7P	786.63712	786.63646	-0.8	4.7E+05
445	Oligomycin A	[M+H]+	C45H74O11	791.53039	791.53257	2.8	1.1E+06
446	1,2-di-(6Z,9Z,12Z,15Z-octadecatetraenoyl)-sn-glycero-3-phospho-(1'-sn-glycerol)	[M+Cl]-	C42H67O10P	797.41659	797.41569	-1.1	1.0E+05
447	SM(d18:0/22:2(13Z,16Z)(OH))	[M+H]+	C45H87N2O7P	799.63237	799.63006	-2.9	1.3E+06
448	1-(1Z-eicosenyl)-2-(9Z-nonadecenoyl)-glycero-3-phospho-(1'-sn-glycerol)	[M-H]-	C45H87O9P	801.60149	801.59948	-2.5	7.1E+04
449	1-(3Z,6Z,9Z,12Z,15Z-octadecapentaenoyl)-2-(6Z,9Z,12Z,15Z-octadecatetraenoyl)-3-O-beta-D-galactosyl-sn-glycerol	[M+Cl]-	C45H68O10	803.45065	803.45122	0.7	1.3E+05
450	PG(16:0/22:2(13Z,16Z))	[M+H]+	C44H83O10P	803.57966	803.58004	0.5	2.8E+06
451	PE(22:2/P-18:1)	[M+Na]+	C45H84NO7P	804.58776	804.58649	-1.6	1.4E+06
452	PG(O-18:0/22:4(7Z,10Z,13Z,16Z))	[M+H]+	C46H85O9P	813.60040	813.60092	0.6	8.1E+05

453	PS(15:0/22:6)	[M+Na]+	C43H72NO10P	816.47860	816.48038	2.2	4.9E+05
454	Laricitrin 3,7,5'-triglucoside	[M-H]-	C34H42O23	817.20441	817.20640	2.4	7.9E+04
455	PI(13:0/20:4)	[M+H]+	C42H73O13P	817.48616	817.48641	0.3	7.0E+05
456	Staphyloxanthin	[M+H]+	C51H78O8	819.57695	819.57794	1.2	1.4E+06
457	Malvidin 3-glucoside-5-(6'''-malonyl-2'''-sulfatoglucoside)	[M-H]-	C32H37O23S	820.13736	820.13729	-0.1	8.6E+04
458	PE(18:1/22:2)	[M+Na]+	C45H84NO8P	820.58268	820.58503	2.9	7.1E+05
459	1-(8-[5]-ladderane-octanyl)-2-(8-[3]-ladderane-octanyl)-sn-glycero-3-phospho-(1'-sn-glycerol)	[M+Cl]-	C46H77O8P	823.50501	823.50740	2.9	8.0E+04
460	PA(22:0/22:6)	[M+Na]+	C47H81O8P	827.55613	827.55556	-0.7	8.3E+05
461	PS(15:0/22:0)	[M+Na]+	C43H84NO10P	828.57251	828.57080	-2.1	4.4E+05
462	3,5-Di-O-galloyl-4-O-digalloylquinic acid	[M+Cl]-	C35H28O22	835.07662	835.07434	-2.7	6.7E+04
463	1-Hexadecanoyl-2-(9Z-octadecenoyl)-sn-glycero-3-phospho-1'-myo-inositol	[M-H]-	C43H81O13P	835.53420	835.53340	-1.0	9.7E+04
464	Isobutyryl-CoA	[M-H]-	C25H42N7O17P3S	836.14980	836.15174	2.3	1.0E+05
465	PI(13:0/20:5)	[M+Na]+	C42H71O13P	837.45245	837.45473	2.7	8.4E+05
466	PG(17:2/22:6)	[M+K]+	C45H73O10P	843.45729	843.45783	0.6	7.2E+05
467	PG(19:0/22:2)	[M+H]+	C47H89O10P	845.62661	845.62809	1.7	6.2E+05
468	PE(20:5/24:1)	[M+H]+	C49H86NO8P	848.61638	848.61828	2.2	1.5E+06
469	SM(d18:2/24:1)	[M+K]+	C47H91N2O6P	849.62463	849.62662	2.3	8.4E+05
470	PI(17:0/14:1)	[M+K]+	C40H78NO13P	850.48424	850.48246	-2.1	4.6E+05
471	PG(18:4/22:6)	[M+K]+	C46H71O10P	853.44164	853.44306	1.7	7.3E+05
472	TG(16:0/16:0/18:2)	[M+Na]+	C53H98O6	853.72556	853.72526	-0.4	5.9E+05
473	PG(18:3/22:6)	[M+K]+	C46H73O10P	855.45729	855.45735	0.1	1.1E+06
474	PI(O-20:0/17:2)	[M+H]+	C46H87O12P	863.60079	863.60218	1.6	1.2E+06
475	1-eicosanoyl-2-(13Z,16Z-docosadienoyl)-glycero-3-phosphoserine	[M-H]-	C48H90NO10P	870.62296	870.62246	-0.6	7.6E+04
476	TG(16:0/16:0/18:1)	[M+K]+	C53H100O6	871.71515	871.71404	-1.3	8.0E+05
477	7-Hydroxylpradimicin A	[M+Na]+	C40H44N2O19	879.24305	879.24285	-0.2	5.8E+05
478	PG(O-20:0/22:4)	[M+K]+	C48H89O9P	879.58758	879.58563	-2.2	6.8E+05
479	1-octadecanoyl-2-(9Z,12Z-octadecadienoyl)-sn-glycero-3-phospho-(1'-sn-glycerol-3'-phosphate)	[M+Cl]-	C42H80O13P2	889.47682	889.47628	-0.6	5.1E+04
480	PI(O-20:0/19:1)	[M+H]+	C48H93O12P	893.64774	893.64640	-1.5	6.8E+05
481	Delphinidin 3-(6''-O-4-malyl-glucoside)-5-(6'''-O-1-malyl-glucoside)	[M+Cl]-	C35H39O25	894.14744	894.14556	-2.1	4.7E+04
482	PI(16:0/20:0)	[M+K]+	C45H87O13P	905.55159	905.55092	-0.7	6.0E+05
483	PG(22:1/22:2)	[M+Na]+	C50H93O10P	907.63986	907.64251	2.9	7.0E+05
484	PE(24:0/24:0)	[M+H]+	C53H106NO8P	916.77288	916.77492	2.2	4.8E+05
485	PI(O-18:0/22:4)	[M+Na]+	C49H89O12P	923.59839	923.59815	-0.3	4.6E+05
486	bacteriohopane-,32,33,34-triol-35-(N-(9-cyclohexyl-nonanoyl))-glucosamine	[M+H]+	C56H99NO9	930.73926	930.74112	2.0	3.7E+05
487	PIP(16:0/18:0)	[M+Na]+	C43H84O16P2	941.51268	941.51459	2.0	3.6E+05

488	Disialyllactose	[M+Na]+	C34H56N2O27	947.29627	947.29904	2.9	3.6E+05
489	TG(16:0/20:1/20:4)[iso6]	[M+K]+	C59H104O6	947.74645	947.74365	-3.0	8.9E+05
490	PI(19:1/22:4)	[M+Na]+	C50H87O13P	949.57765	949.57959	2.0	5.1E+05
491	Bis(5'-adenosyl) pentaphosphate	[M+Cl]-	C20H29N10O22P5	950.98400	950.98282	-1.2	1.2E+05
492	1,4-Dihydroxy-2-naphthoyl-CoA	[M-H]-	C32H42N7O19P3S	952.13963	952.14124	1.7	6.1E+04
493	3,5-Dihydroxyphenylacetyl-CoA	[M+K]+	C29H42N7O19P3S	956.11006	956.11173	1.7	3.5E+05
494	1-octadecanoyl-2-docosanoyl-glycero-3-phospho-(1'-myo-inositol)	[M+Cl]-	C49H95O13P	957.62043	957.61849	-2.0	8.1E+04
495	Patuletin 3-(2''-feruloylglucosyl)-(1->6)-[apiosyl-(1->2)-glucoside]	[M-H]-	C43H48O25	963.24119	963.24203	0.9	1.2E+05
496	TG(17:1/22:6)	[M+H]+	C64H98O6	963.74362	963.74469	1.1	1.0E+06
497	PC(22:1/24:1)	[M+K]+	C54H104NO8P	964.71312	964.71218	-1.0	4.5E+05
498	TG(17:1/20:1/20:1)	[M+K]+	C60H110O6	965.79340	965.79520	1.9	3.7E+05
499	PIP2(16:0/16:2)	[M+H]+	C41H77O19P3	967.43447	967.43211	-2.4	3.4E+05
500	1-(11Z-eicosenoyl)-2-(7Z,10Z,13Z,16Z-docosatetraenoyl)-glycero-3-phospho-(1'-myo-inositol)	[M+Cl]-	C51H89O13P	975.57348	975.57113	-2.4	1.3E+05
501	PI(20:2/22:4)	[M+K]+	C51H87O13P	977.55159	977.54885	-2.8	5.0E+05
502	TG(22:5/18:2/22:6)	[M+H]+	C65H100O6	977.75927	977.76163	2.4	4.0E+05
503	2-O-nonadecanoyl-3-O-(2S,4S-dimethyl-tetracosanoyl)-alpha,alpha-trehalose	[M-H]-	C57H108O13	999.77172	999.77139	-0.3	1.2E+05

[a]Cer: Ceramide; GalCer: Galactosylceramide; GlcCer: Glucosylceramide; Glc-GP: phosphatidylglucose; LacCer: Lactosylceramide; MG: Monoacylglycerol; DAT: Acyltrehaloses; DG: Diacylglycerol; TG: Triacylglycerol; MGDG: Monoacyldiacylglycerol; PA: Phosphatidic acid; PC: Phosphatidylcholine; PE: Phosphatidylethanolamine; PG: Glycerophospholipids; PI: Phosphatidylinositol; PIP2: phosphatidylinositol bisphosphate; PS: Phosphatidylserine; SM: Sphingomyelin.

[b]Theor. stands for calculated exact mass to charge ratio.

[c]Exp. stands for experimental *m*/*z* value.

[d]The error expressed in parts per million (ppm).

Table S4. ESI FT-ICR MS comprehensive list of metabolites detected in SurM 10K.

#	Putative Annotation (M)[a]	Ion	Formula	Theor. *m/z*[b]	Exp. *m/z*[c]	ppm[d]	Peak_Height
1	Putrescine	[M-H]-	C4H12N2	87.09277	87.09280	0.3	2.6E+04
2	2-Aminoacrylate	[M+H]+	C3H5NO2	88.03930	88.03933	0.3	4.2E+04
3	Pyruvic acid	[M+H]+	C3H4O3	89.02332	89.02335	0.3	6.3E+04
4	1-Pentanol	[M+H]+	C5H12O	89.09609	89.09601	-0.9	4.4E+04
5	2,3-Butanediol	[M+H]+	C4H10O2	91.07536	91.07556	2.2	5.8E+04
6	Vinyl ether	[M+Na]+	C4H6O	93.03109	93.03130	2.3	5.6E+04
7	Sulfuric acid	[M-H]-	H2SO4	96.96010	96.96033	2.3	2.7E+05
8	Orthophosphate	[M-H]-	H3PO4	96.96962	96.96971	0.9	1.9E+05
9	Isopropylamine	[M+K]+	C3H9N	98.03666	98.03690	2.5	4.1E+04
10	Propyl mercaptan	[M+Na]+	C3H8S	99.02389	99.02374	-1.5	3.5E+04
11	Cyclohexylamine	[M+H]+	C6H13N	100.11208	100.11216	0.8	4.3E+04
12	*N*-Nitrosodiethylamine	[M+H]+	C4H10N2O	103.08659	103.08680	2.0	4.5E+04
13	Sulfite	[M+Na]+	H2SO3	104.96169	104.96172	0.3	4.4E+04
14	Styrene	[M+H]+	C8H8	105.06988	105.06996	0.8	5.0E+04
15	Thiocarbohydrazide	[M+H]+	CH6N4S	107.03859	107.03870	1.0	6.1E+04
16	1-Pyrroline	[M+K]+	C4H7N	108.02101	108.02101	0.0	3.6E+04
17	Pyruvaldehyde	[M+K]+	C3H4O2	110.98429	110.98438	0.8	3.7E+04
18	2-Furoic acid	[M-H]-	C5H4O3	111.00877	111.00879	0.2	8.1E+04
19	Nitrofuran	[M-H]-	C4H3NO3	112.00402	112.00384	-1.6	1.5E+04
20	2-Furoic acid	[M+H]+	C5H4O3	113.02332	113.02353	1.9	3.9E+04
21	2-hexenedial	[M+H]+	C6H8O2	113.05971	113.05939	-2.8	3.5E+04
22	Glycine	[M+K]+	C2H5NO2	113.99519	113.99515	-0.3	5.3E+04
23	Fumaric acid	[M-H]-	C4H4O4	115.00368	115.00391	2.0	3.0E+04
24	L-Valine	[M+H]+	C5H11NO2	118.08626	118.08599	-2.2	9.6E+04
25	Purine	[M-H]-	C5H4N4	119.03632	119.03604	-2.4	1.3E+04
26	Maleimide	[M+Na]+	C4H3NO2	120.00560	120.00547	-1.1	3.8E+04
27	Hexenal	[M+Na]+	C6H10O	121.06239	121.06240	0.1	4.2E+04
28	Formyl phosphate	[M-H]-	CH3O5P	124.96453	124.96467	1.1	1.9E+04
29	Valeric acid	[M+Na]+	C5H10O2	125.05730	125.05737	0.6	4.6E+04
30	Gamma-Aminobutyric acid	[M+Na]+	C4H9NO2	126.05255	126.05219	-2.8	4.1E+04
31	*N*-Cyclohexylformamide	[M-H]-	C7H13NO	126.09244	126.09238	-0.5	1.2E+04
32	Alanine	[M+K]+	C3H7NO2	128.01084	128.01072	-0.9	3.8E+04
33	4-Oxoproline	[M-H]-	C5H7NO3	128.03532	128.03532	0.0	1.1E+05
34	Lactic acid	[M+K]+	C3H6O3	128.99485	128.99524	3.0	7.1E+04
35	2-methyl-2Z-hexenoic acid	[M+H]+	C7H12O2	129.09101	129.09065	-2.8	4.2E+04
36	L-Leucine	[M-H]-	C6H13NO2	130.08735	130.08725	-0.8	1.3E+05

#	Putative Annotation (M)[a]	Ion	Formula	Theor. *m/z*[b]	Exp. *m/z*[c]	ppm[d]	Peak_Height
37	Glycerol	[M+K]+	C3H8O3	131.01050	131.01085	2.6	9.3E+04
38	L-Asparagine	[M-H]-	C4H8N2O3	131.04622	131.04592	-2.3	2.6E+04
39	Cresol	[M+Na]+	C7H8O	131.04674	131.04653	-1.6	3.7E+04
40	Indoleamine	[M-H]-	C8H8N2	131.06147	131.06145	-0.2	1.8E+04
41	L-Aspartic acid	[M-H]-	C4H7NO4	132.03023	132.03009	-1.1	2.7E+05
42	L-Leucine	[M+H]+	C6H13NO2	132.10191	132.10199	0.6	1.3E+05
43	Dimethyl sulfone	[M+K]+	C2H6O2S	132.97201	132.97195	-0.5	4.2E+04
44	L-Malic acid	[M-H]-	C4H6O5	133.01425	133.01430	0.4	8.5E+05
45	Phenylacetaldoxime	[M-H]-	C8H9NO	134.06114	134.06128	1.1	4.7E+04
46	(+)-3-Carene	[M-H]-	C10H16	135.11792	135.11783	-0.7	3.2E+04
47	Adenine	[M+H]+	C5H5N5	136.06177	136.06188	0.8	6.7E+04
48	2-Phenylacetamide	[M+H]+	C8H9NO	136.07569	136.07601	2.3	5.1E+04
49	8-Hydroxypurine	[M-H]-	C5H6N4O	137.04688	137.04704	1.1	3.9E+04
50	N-Methylnicotinamide	[M+H]+	C7H8N2O	137.07094	137.07131	2.7	3.8E+04
51	L-Proline	[M+Na]+	C5H9NO2	138.05255	138.05233	-1.6	4.9E+04
52	Tiglic acid	[M+K]+	C5H8O2	139.01559	139.01566	0.5	6.3E+04
53	1-Hexen-1-ol	[M+K]+	C6H12O	139.05197	139.05186	-0.8	4.6E+04
54	5-Aminopentanal	[M+K]+	C5H11NO	140.04722	140.04709	-1.0	5.1E+04
55	Betaine	[M+Na]+	C5H11NO2	140.06820	140.06802	-1.3	7.2E+04
56	2-Mercaptoethanesulfonate	[M-H]-	C2H6O3S2	140.96856	140.96830	-1.8	1.7E+04
57	2-Octenoic acid	[M-H]-	C8H14O2	141.09210	141.09251	2.9	2.7E+04
58	Proline betaine	[M-H]-	C7H13NO2	142.08735	142.08749	1.0	1.0E+05
59	cis,trans-Hexadienedioate	[M+H]+	C6H6O4	143.03389	143.03423	2.4	4.7E+04
60	3-Hexenedioic acid	[M-H]-	C6H8O4	143.03498	143.03491	-0.5	2.0E+04
61	1-(3-Aminopropyl)-4-aminobutanal	[M-H]-	C7H16N2O	143.11899	143.11906	0.5	9.3E+03
62	Benzaldehyde	[M+K]+	C7H6O	145.00502	145.00490	-0.9	3.9E+04
63	L-Glutamine	[M-H]-	C5H10N2O3	145.06187	145.06225	2.7	3.3E+04
64	L-Glutamic acid	[M-H]-	C5H9NO4	146.04588	146.04578	-0.7	4.0E+05
65	4-Guanidinobutanoate	[M+H]+	C5H11N3O2	146.09240	146.09206	-2.3	5.3E+04
66	L-Lysinamide	[M+H]+	C6H15N3O	146.12879	146.12894	1.0	6.4E+04
67	L-Lysine	[M+H]+	C6H14N2O2	147.11280	147.11285	0.3	4.5E+05
68	Dimethyl trisulfide	[M+Na]+	C2H6S3	148.95238	148.95250	0.8	4.1E+04
69	2,5-Dihydroxypyridine	[M+K]+	C5H5NO2	149.99519	149.99557	2.5	8.1E+04
70	Cytosine	[M+K]+	C4H5N3O	150.00642	150.00663	1.4	4.3E+04
71	Thiobenzamide S-oxide	[M-H]-	C7H7NOS	152.01756	152.01785	1.9	4.2E+04
72	Quinoline	[M+Na]+	C9H7N	152.04707	152.04669	-2.5	1.1E+05
73	Dopamine	[M-H]-	C8H11NO2	152.07170	152.07210	2.6	2.7E+04
74	Dihydrouracil	[M+K]+	C4H6N2O2	153.00609	153.00574	-2.3	4.1E+04

#	Putative Annotation (M)[a]	Ion	Formula	Theor. *m/z*[b]	Exp. *m/z*[c]	ppm[d]	Peak_Height
75	2Z-Hexenoic acid	[M+K]+	C6H10O2	153.03124	153.03114	-0.6	1.1E+05
76	Proline	[M+K]+	C5H9NO2	154.02649	154.02687	2.5	1.1E+05
77	Leucine	[M+Na]+	C6H13NO2	154.08385	154.08431	3.0	3.4E+05
78	Glycerol 1,2-cyclic phosphate	[M+H]+	C3H7O5P	155.01039	155.01065	1.7	7.4E+04
79	2,3-Dihydroxybenzoate	[M+H]+	C7H6O4	155.03389	155.03360	-1.8	4.2E+04
80	Dicyclopentadiene	[M+Na]+	C10H12	155.08312	155.08278	-2.2	5.6E+04
81	L-1-Aminopropan-2-ol O-phosphate	[M+H]+	C3H10NO4P	156.04202	156.04231	1.9	5.1E+05
82	Histidine	[M+H]+	C6H9N3O2	156.07675	156.07685	0.6	1.2E+05
83	Cinnamyl alcohol	[M+Na]+	C9H10O	157.06239	157.06261	1.4	6.9E+05
84	1,2-Naphthoquinone	[M+H]+	C10H6O2	159.04406	159.04424	1.2	4.5E+04
85	D-Alanyl-D-alanine	[M-H]-	C6H12N2O3	159.07752	159.07784	2.0	1.1E+04
86	Cysteine	[M+K]+	C3H7NO2S	159.98291	159.98288	-0.2	5.1E+04
87	2-Deoxy-3-keto-scyllo-inosamine	[M-H]-	C6H11NO4	160.06153	160.06187	2.1	4.0E+04
88	3-Hydroxybenzaldehyde	[M+K]+	C7H6O2	160.99994	161.00014	1.2	5.9E+04
89	3-Ethylcatechol	[M+Na]+	C8H10O2	161.05730	161.05773	2.7	5.5E+04
90	3,4-Dihydroxybenzylamine	[M+Na]+	C7H9NO2	162.05255	162.05258	0.2	5.1E+04
91	(R)-Nicotine	[M+H]+	C10H14N2	163.12297	163.12304	0.4	7.8E+04
92	(2-Aminoethyl)phosphonate	[M+K]+	C2H8NO3P	163.98734	163.98715	-1.2	4.8E+04
93	D-Phenylalanine	[M-H]-	C9H11NO2	164.07170	164.07185	0.9	7.4E+04
94	6-Dimethylaminopurine	[M+H]+	C7H9N5	164.09307	164.09293	-0.9	6.4E+04
95	2-Dehydro-D-xylonate	[M+H]+	C5H8O6	165.03936	165.03983	2.8	7.3E+04
96	1-Methylxanthine	[M-H]-	C6H6N4O2	165.04180	165.04139	-2.5	2.1E+04
97	2,3,6-Trihydroxypyridine	[M+K]+	C5H5NO3	165.99010	165.98978	-1.9	7.2E+04
98	3-Hydroxy-L-proline	[M+Cl]-	C5H9NO3	166.02764	166.02793	1.7	4.9E+04
99	3-Pyridinebutanoic acid	[M+H]+	C9H11NO2	166.08626	166.08623	-0.2	1.5E+05
100	Leucine	[M+K]+	C6H13NO2	170.05779	170.05807	1.7	2.8E+05
101	Asparagine	[M+K]+	C4H8N2O3	171.01665	171.01690	1.5	7.3E+04
102	Propylthiouracil	[M+H]+	C7H10N2OS	171.05866	171.05856	-0.6	6.5E+04
103	Ureidoglycine	[M+K]+	C3H7N3O3	172.01190	172.01195	0.3	5.0E+04
104	Phenylpropanoic acid	[M+Na]+	C9H10O2	173.05730	173.05774	2.5	2.5E+05
105	2-Octenedioic acid	[M+H]+	C8H12O4	173.08084	173.08124	2.3	1.2E+05
106	2E,8E-Undecadiene-4,6-diynoic acid	[M+H]+	C11H10O2	175.07536	175.07485	-2.9	1.1E+05
107	L-Arginine	[M+H]+	C6H14N4O2	175.11895	175.11894	-0.1	1.1E+06
108	Tyramine	[M+K]+	C8H11NO	176.04722	176.04728	0.3	5.4E+04
109	L-Hypoglycin	[M+Cl]-	C7H11NO2	176.04838	176.04841	0.2	1.4E+04
110	L-Histidinol	[M+Cl]-	C6H11N3O	176.05961	176.05927	-2.0	3.9E+04
111	Thien-2-ylacetate	[M+Cl]-	C6H6O2S	176.97825	176.97867	2.4	9.7E+03
112	2-Methylnaphthalene	[M+Cl]-	C11H10	177.04765	177.04814	2.8	1.1E+04

#	Putative Annotation (M)[a]	Ion	Formula	Theor. *m/z*[b]	Exp. *m/z*[c]	ppm[d]	Peak_Height
113	Allantoic acid	[M+H]+	C4H8N4O4	177.06183	177.06222	2.2	8.0E+04
114	5-Methyl-3-isoxazolyl sulfate	[M-H]-	C4H5NO5S	177.98157	177.98168	0.6	3.0E+04
115	Dehydrospermidine	[M+Cl]-	C7H17N3	178.11165	178.11184	1.1	2.8E+04
116	2,3,5-Trihydroxytoluene	[M+K]+	C7H8O3	179.01050	179.01018	-1.8	5.3E+04
117	2E,5-Hexadienyl acetate	[M+K]+	C8H12O2	179.04689	179.04651	-2.1	7.7E+04
118	D-Aldose	[M-H]-	C6H12O6	179.05611	179.05632	1.2	2.1E+05
119	Capraldehyde	[M+Na]+	C10H20O	179.14064	179.14086	1.3	5.4E+04
120	5-Nitrofurfural	[M+K]+	C5H3NO4	179.96937	179.96895	-2.3	5.7E+04
121	D-Tyrosine	[M-H]-	C9H11NO3	180.06662	180.06631	-1.7	3.0E+04
122	Glucosamine	[M+H]+	C6H13NO5	180.08665	180.08678	0.7	2.1E+05
123	2-Octenoic acid	[M+K]+	C8H14O2	181.06254	181.06280	1.4	9.5E+04
124	8-Hydroxy-7-methylguanine	[M+H]+	C6H7N5O2	182.06725	182.06707	-1.0	5.8E+04
125	Acenaphthenequinone	[M+H]+	C12H6O2	183.04406	183.04412	0.4	5.6E+04
126	Diisopropyl phosphate	[M+H]+	C6H15O4P	183.07807	183.07792	-0.8	7.3E+04
127	2-Thiophenesulfonamide	[M+Na]+	C4H5NO2S2	185.96539	185.96484	-3.0	5.1E+04
128	D-Glutamic acid	[M+K]+	C5H9NO4	186.01632	186.01654	1.2	5.4E+04
129	3-Hydroxy-L-glutamate	[M+Na]+	C5H9NO5	186.03729	186.03717	-0.7	9.0E+04
130	2-Deoxy-scyllo-inosamine	[M+Na]+	C6H13NO4	186.07368	186.07407	2.1	7.4E+04
131	(+/-)-trans-Acenaphthene-1,2-diol	[M+H]+	C12H10O2	187.07536	187.07509	-1.4	6.2E+04
132	Trimethyl-L-lysine	[M-H]-	C9H20N2O2	187.14520	187.14480	-2.1	3.4E+04
133	D-Methionine	[M+K]+	C5H11NO2S	188.01421	188.01449	1.5	6.6E+04
134	L-Phenylalanine	[M+Na]+	C9H11NO2	188.06820	188.06828	0.4	1.9E+05
135	Phthalic acid	[M+Na]+	C8H6O4	189.01583	189.01553	-1.6	1.3E+05
136	2-tridecene-4,7-diynal	[M+H]+	C13H16O	189.12739	189.12713	-1.4	8.8E+04
137	Thioguanine	[M+Na]+	C5H5N5S	190.01579	190.01612	1.8	8.4E+04
138	N-Amidino-L-glutamate	[M+H]+	C6H11N3O4	190.08223	190.08270	2.5	5.7E+04
139	Citric acid	[M-H]-	C6H8O7	191.01973	191.01974	0.1	1.0E+06
140	Vanillic acid	[M+Na]+	C8H8O4	191.03148	191.03102	-2.4	7.5E+04
141	1,2-Dihydroxy-7-hydroxymethylnaphthalene	[M+H]+	C11H10O3	191.07027	191.06992	-1.8	8.4E+04
142	(+)-Camphor	[M+K]+	C10H16O	191.08327	191.08311	-0.9	5.5E+04
143	Isopropylmaleate	[M+Cl]-	C7H10O4	193.02731	193.02692	-2.0	2.9E+04
144	1,2-Epoxy-3-(p-Nitrophenoxy)propane	[M-H]-	C9H9NO4	194.04588	194.04618	1.5	1.3E+04
145	5-oxo-7-octenoic acid	[M+K]+	C8H12O3	195.04180	195.04203	1.2	8.0E+04
146	4-Hydroxynonenal	[M+K]+	C9H16O2	195.07819	195.07826	0.4	1.1E+05
147	Coryneine	[M-H]-	C11H18NO2	195.12648	195.12680	1.7	4.2E+04
148	1-Nitrosonaphthalene	[M+K]+	C10H7NO	196.01592	196.01588	-0.2	7.0E+04
149	Nicotine imine	[M+Cl]-	C10H13N2	196.07728	196.07750	1.2	3.7E+04
150	Putreanine	[M+Cl]-	C7H17N2O2	196.09840	196.09820	-1.0	1.9E+04

#	Putative Annotation (M)[a]	Ion	Formula	Theor. *m/z*[b]	Exp. *m/z*[c]	ppm[d]	Peak_Height
151	Phenolic phosphate	[M+Na]+	C6H7O4P	196.99742	196.99776	1.7	8.1E+04
152	N-Acetyl-L-aspartate	[M+Na]+	C6H9NO5	198.03729	198.03737	0.4	9.4E+04
153	2-Amino-5-phosphopentanoic acid	[M+H]+	C5H12NO5P	198.05259	198.05243	-0.8	1.4E+05
154	Phosphoenol-4-deoxy-3-tetrulosonate	[M+H]+	C4H7O7P	199.00022	199.00007	-0.7	6.5E+04
155	10-hydroxy-8E-Decene-2,4,6-triynoic acid	[M+Na]+	C10H8O3	199.03656	199.03615	-2.1	7.7E+04
156	2R-hydroxy-octanoic acid	[M+K]+	C8H16O3	199.07310	199.07359	2.4	9.7E+04
157	Dodecanoic acid	[M-H]-	C12H24O2	199.17035	199.17070	1.7	6.2E+04
158	L-Carnitine	[M+K]+	C7H15NO3	200.06835	200.06858	1.1	6.4E+04
159	Nicotine imine	[M+K]+	C10H13N2	200.07103	200.07123	1.0	7.0E+04
160	1-Ethyl-2-benzimidazolinone	[M+K]+	C9H10N2O	201.04247	201.04230	-0.9	7.9E+04
161	1-Phenylpropyl acetate	[M+Na]+	C11H14O2	201.08860	201.08908	2.4	2.4E+05
162	(5-Phenyl-1,2,4-triazol-3-yl)urea	[M-H]-	C9H9N5O	202.07343	202.07302	-2.1	1.2E+04
163	D-Aldose	[M+Na]+	C6H12O6	203.05261	203.05265	0.2	9.1E+06
164	Pantothenol	[M-H]-	C9H19NO4	204.12413	204.12439	1.3	1.2E+04
165	D-Sorbitol	[M+Na]+	C6H14O6	205.06826	205.06816	-0.5	1.4E+05
166	2-dodecenal	[M+Na]+	C12H22O	205.15629	205.15669	2.0	7.2E+04
167	Tetradecatrienal	[M-H]-	C14H22O	205.15979	205.15980	0.1	3.2E+04
168	4-Toluenesulfonamide	[M+Cl]-	C7H9NO2S	206.00480	206.00435	-2.2	2.2E+04
169	Cyclo(deltaAla-L-Val)	[M+K]+	C8H12N2O2	207.05304	207.05322	0.9	1.2E+05
170	Benzidine	[M+Na]+	C12H12N2	207.08927	207.08983	2.7	1.0E+05
171	Phenolic phosphate	[M+Cl]-	C6H7O4P	208.97760	208.97798	1.8	3.1E+04
172	2-Propylglutaric acid	[M+Cl]-	C8H14O4	209.05861	209.05911	2.4	3.9E+04
173	Tyr-Oet	[M+H]+	C11H15NO3	210.11247	210.11217	-1.4	1.3E+05
174	Nonanedioic acid	[M+Na]+	C9H16O4	211.09408	211.09444	1.7	7.4E+04
175	1,4-Diguanidinobutane	[M+K]+	C6H16N6	211.10680	211.10652	-1.3	1.4E+05
176	Phospho-L-aspartate	[M-H]-	C4H8NO7P	211.99656	211.99664	0.4	1.5E+04
177	Benzyl nicotinate	[M-H]-	C13H11NO2	212.07170	212.07125	-2.1	7.1E+04
178	1-Phenyl-5-mercaptotetrazole	[M+Cl]-	C7H6N4S	213.00072	213.00033	-1.8	7.2E+04
179	7-Cyano-7-deazaguanine	[M+K]+	C7H5N5O	214.01257	214.01201	-2.6	9.4E+04
180	2-C-Methyl-D-erythritol 4-phosphate	[M-H]-	C5H13O7P	215.03261	215.03297	1.7	6.9E+05
181	Undecanedioic acid	[M+H]+	C11H20O4	217.14344	217.14388	2.0	8.0E+04
182	(R)-3-Hydroxydodecanoic acid	[M+H]+	C12H24O3	217.17982	217.17947	-1.6	7.3E+04
183	4-Nitrophenyl phosphate	[M-H]-	C6H6NO6P	217.98600	217.98661	2.8	4.4E+04
184	N-gamma-Nitro-L-arginine	[M-H]-	C6H13N5O4	218.08948	218.08951	0.2	1.2E+04
185	Aldohexose	[M+K]+	C6H12O6	219.02655	219.02654	0.0	1.4E+07
186	cis-2'-Carboxybenzalpyruvate	[M+H]+	C11H8O5	221.04445	221.04486	1.9	2.2E+05
187	4-Amino-2-hydroxylamino-6-nitrotoluene	[M+K]+	C7H9N3O3	222.02755	222.02777	1.0	9.6E+04
188	2,6-Diamino-4-hydroxy-5-N-methylformamidopyrimidine	[M+K]+	C6H9N5O2	222.03878	222.03923	2.0	8.6E+04

#	Putative Annotation (M)[a]	Ion	Formula	Theor. *m/z*[b]	Exp. *m/z*[c]	ppm[d]	Peak_Height
189	2E-Decenedioic acid	[M+Na]+	C10H16O4	223.09408	223.09422	0.6	1.1E+05
190	Methyl jasmonate	[M-H]-	C13H20O3	223.13397	223.13366	-1.4	2.6E+04
191	cis-2-Carboxycyclohexyl-acetic acid	[M+K]+	C9H14O4	225.05237	225.05240	0.1	7.7E+04
192	5-Acetylamino-6-formylamino-3-methyluracil	[M+H]+	C8H10N4O4	227.07748	227.07751	0.1	1.5E+05
193	(R)-3-Hydroxydecanoic acid	[M+K]+	C10H20O3	227.10440	227.10506	2.9	7.6E+04
194	Myristic acid	[M-H]-	C14H28O2	227.20165	227.20216	2.2	2.0E+05
195	Prenyl-L-cysteine	[M+K]+	C8H15NO2S	228.04551	228.04506	-2.0	9.5E+04
196	3-Hydroxy-N6,N6,N6-trimethyl-L-lysine	[M+Na]+	C9H21N2O3	228.14444	228.14464	0.9	1.6E+05
197	L-alpha-Amino-gamma-oxalylaminobutyric acid	[M+K]+	C6H10N2O5	229.02213	229.02266	2.3	1.7E+05
198	Dihydroresveratrol	[M-H]-	C14H14O3	229.08702	229.08635	-2.9	2.6E+04
199	N-Acetyl-D-phenylalanine	[M+Na]+	C11H13NO3	230.07876	230.07841	-1.5	9.0E+04
200	2-Deoxy-2-dimethylamino-alpha-D-Glucose	[M+Na]+	C8H17NO5	230.09989	230.10033	1.9	9.6E+04
201	13-amino-tridecanoic acid	[M+H]+	C13H27NO2	230.21146	230.21151	0.2	1.2E+05
202	1,3-Diphenylpropane	[M+Cl]-	C15H16	231.09460	231.09423	-1.6	6.9E+04
203	3-Carbamoyl-2-phenylpropionic acid	[M+Na]+	C10H11NO4	232.05803	232.05768	-1.5	1.1E+05
204	N-Succinyl-L-glutamate 5-semialdehyde	[M+H]+	C9H13NO6	232.08156	232.08220	2.7	8.7E+04
205	1-O-Methyl-myo-inositol	[M+K]+	C7H14O6	233.04220	233.04266	2.0	1.4E+05
206	(R)-(Homo)3-citrate	[M-H]-	C9H14O7	233.06668	233.06653	-0.6	1.5E+04
207	tridecanal	[M+Cl]-	C13H26O	233.16777	233.16771	-0.2	1.3E+04
208	3,7-Dimethyl-8,11-dioxo-2E,6E,9E-dodecatrienal	[M+H]+	C14H18O3	235.13287	235.13276	-0.5	1.3E+05
209	N-Heptanoylhomoserine lactone	[M+Na]+	C11H19NO3	236.12571	236.12521	-2.1	1.9E+05
210	2,4-Dinitrophenylhydrazine	[M+K]+	C6H6N4O4	237.00206	237.00268	2.6	9.9E+04
211	(+)-(1R,2R)-1,2-Diphenylethane-1,2-diol	[M+Na]+	C14H14O2	237.08860	237.08829	-1.3	1.0E+05
212	10,12-hexadecadienal	[M+H]+	C16H28O	237.22129	237.22107	-0.9	9.8E+04
213	Succinyl proline	[M+Na]+	C9H13NO5	238.06859	238.06811	-2.0	9.5E+04
214	Oxaloglutarate	[M+Cl]-	C7H8O7	238.99640	238.99581	-2.5	1.5E+04
215	2E-Decenedioic acid	[M+K]+	C10H16O4	239.06802	239.06776	-1.1	3.5E+05
216	N6-Acetyl-N6-hydroxy-L-lysine	[M+Cl]-	C8H16N2O4	239.08041	239.08016	-1.0	1.4E+05
217	10Z-Tridecenyl acetate	[M-H]-	C15H28O2	239.20165	239.20134	-1.3	2.4E+04
218	(1R,6R)-2-Succinyl-6-hydroxy-2,4-cyclohexadiene-1-carboxylate	[M+H]+	C11H12O6	241.07066	241.07124	2.4	1.4E+05
219	Thymidine	[M-H]-	C10H14N2O5	241.08300	241.08269	-1.3	2.7E+05
220	Indolepyruvate	[M+K]+	C11H9NO3	242.02140	242.02139	-0.1	1.4E+05
221	3-(Phosphoacetylamido)-L-alanine	[M+H]+	C5H11N2O7P	243.03766	243.03732	-1.4	1.1E+05
222	12-methyl-tetradecanoic acid	[M+H]+	C15H30O2	243.23186	243.23182	-0.2	1.2E+05
223	1-Hexadecanol	[M+H]+	C16H34O	243.26824	243.26874	2.0	9.4E+04
224	3-(Dimethylamino)propyl benzoate	[M+K]+	C12H17NO2	246.08909	246.08940	1.3	1.1E+05
225	beta-Butoxyethyl nicotinate	[M+Na]+	C12H17NO3	246.11006	246.10937	-2.8	8.7E+04
226	beta-Alanyl-L-arginine	[M+H]+	C9H19N5O3	246.15607	246.15607	0.0	2.5E+05

#	**Putative Annotation (M)**[a]	**Ion**	**Formula**	**Theor. *m/z***[b]	**Exp. *m/z***[c]	**ppm**[d]	**Peak_Height**
227	N-Acetyl-D-tryptophan	[M+H]+	C13H14N2O3	247.10772	247.10826	2.2	9.3E+04
228	L-beta-aspartyl-L-leucine	[M+H]+	C10H18N2O5	247.12885	247.12887	0.1	9.1E+04
229	Abscisic aldehyde	[M-H]-	C15H20O3	247.13397	247.13426	1.2	4.8E+04
230	Pyridoxal 5'-phosphate	[M+H]+	C8H10NO6P	248.03185	248.03178	-0.3	1.3E+05
231	2-Hydroxyiminostilbene	[M+K]+	C14H11NO	248.04722	248.04683	-1.6	1.4E+05
232	Deoxycytidine	[M+Na]+	C9H13N3O4	250.07983	250.07913	-2.8	8.4E+04
233	Bergapten	[M+Cl]-	C12H8O4	251.01166	251.01166	0.0	1.5E+04
234	Tetradecadiene-4,6-diynoic acid	[M+Cl]-	C14H16O2	251.08443	251.08421	-0.9	3.5E+04
235	Diisopropyl phthalate	[M+H]+	C14H18O4	251.12779	251.12717	-2.5	1.1E+05
236	(+)-(1R,2R)-1,2-Diphenylethane-1,2-diol	[M+K]+	C14H14O2	253.06254	253.06242	-0.5	8.8E+04
237	Palmitoleic acid	[M-H]-	C16H30O2	253.21730	253.21770	1.6	2.2E+05
238	Succinyl proline	[M+K]+	C9H13NO5	254.04253	254.04236	-0.7	1.9E+05
239	Palmitic acid	[M-H]-	C16H32O2	255.23295	255.23279	-0.6	4.7E+05
240	N-D-Glucosylarylamine	[M+H]+	C12H17NO5	256.11795	256.11829	1.3	1.2E+05
241	2E,4E,8E,10E-Dodecatetraenedioic acid	[M+K]+	C12H14O4	261.05237	261.05223	-0.5	3.4E+05
242	7E,9E,11-Dodecatrienyl acetate	[M+K]+	C14H22O2	261.12514	261.12459	-2.1	9.8E+04
243	Thiamine aldehyde	[M+H]+	C12H15N4OS	264.10393	264.10334	-2.2	1.2E+05
244	3,7,11-Trimethyl-2,6,10-dodecatrienyl acetate	[M+H]+	C17H28O2	265.21621	265.21680	2.2	2.0E+05
245	D-erythro-1-(Imidazol-4-yl)glycerol 3-phosphate	[M+Cl]-	C6H11N2O6P	273.00487	273.00560	2.7	6.5E+04
246	Sarmentosin	[M-H]-	C11H17NO7	274.09323	274.09261	-2.3	7.6E+04
247	Oxyresveratrol	[M+Cl]-	C14H12O4	279.04296	279.04249	-1.7	4.7E+04
248	3,7,11-trimethyl-dodecanoic acid	[M+K]+	C15H30O2	281.18774	281.18854	2.8	2.7E+05
249	Oleic acid	[M-H]-	C18H34O2	281.24860	281.24938	2.8	6.2E+05
250	Octadecanoic acid	[M-H]-	C18H36O2	283.26425	283.26364	-2.2	1.0E+06
251	2-(Formamido)-N1-(5-phospho-D-ribosyl)acetamidine	[M+H]+	C6H12N3O8P	286.04348	286.04281	-2.3	1.1E+05
252	Thiamine	[M+Na]+	C12H17N4OS	288.10153	288.10143	-0.3	1.1E+05
253	Nicotinate D-ribonucleoside	[M+Cl]-	C11H14NO6	291.05151	291.05089	-2.1	3.0E+04
254	Palmitic acid	[M+K]+	C16H32O2	295.20339	295.20360	0.7	2.3E+05
255	3,5,7-Trihydroxy-6,8-dimethylflavone	[M-H]-	C17H14O5	297.07685	297.07628	-1.9	2.6E+05
256	7,7-Difluoro-8Z-dodecenyl acetate	[M+Cl]-	C14H24F2O2	297.14384	297.14325	-2.0	5.1E+04
257	13S-hydroxy-9Z,11E-octadecadienoic acid	[M+H]+	C18H32O3	297.24242	297.24231	-0.4	2.7E+05
258	PC(2:0/0:0)	[M-H]-	C10H22NO7P	298.10611	298.10548	-2.1	3.2E+05
259	Serratamic acid	[M+Na]+	C13H25NO5	298.16249	298.16313	2.1	1.5E+05
260	2-Ethylhexyl phthalate	[M+Na]+	C16H22O4	301.14103	301.14068	-1.2	1.2E+06
261	Thymidine 3',5'-cyclic monophosphate	[M-H]-	C10H13N2O7P	303.03876	303.03879	0.1	8.2E+04
262	9-Riburonosyladenine	[M+Na]+	C10H11N5O5	304.06524	304.06516	-0.3	1.0E+05
263	2'-Deoxymugineic acid	[M+H]+	C12H20N2O7	305.13433	305.13369	-2.1	1.9E+05
264	4-Hydroxytestosterone	[M+H]+	C19H28O3	305.21112	305.21169	1.9	1.1E+05

#	Putative Annotation (M)[a]	Ion	Formula	Theor. *m/z*[b]	Exp. *m/z*[c]	ppm[d]	Peak_Height
265	Adenosine	[M+K]+	C10H13N5O4	306.05991	306.05906	-2.8	1.1E+05
266	Z-Gly-Pro	[M+H]+	C15H18N2O5	307.12885	307.12929	1.4	2.3E+05
267	Heptadecanediol	[M+Cl]-	C17H36O2	307.24093	307.24063	-1.0	2.3E+04
268	(1R)-Hydroxy-(2R)-N-acetyl-L-cysteinyl-1,2-dihydronaphthalene	[M+H]+	C15H17NO4S	308.09511	308.09514	0.1	2.8E+05
269	N-Acetyl-a-neuraminic acid	[M-H]-	C11H19NO9	308.09870	308.09859	-0.4	2.5E+05
270	5-Amino-6-(1-D-ribitylamino)uracil	[M+Cl]-	C9H16N4O6	311.07639	311.07720	2.6	1.2E+05
271	L-Aspartyl-L-phenylalanine	[M+Cl]-	C13H16N2O5	315.07532	315.07447	-2.7	1.3E+05
272	1-O-Hexadecyl-sn-glycerol	[M-H]-	C19H40O3	315.29047	315.28968	-2.5	1.2E+05
273	Saphenic acid methyl ester	[M+Cl]-	C16H14N2O3	317.06984	317.06964	-0.6	4.2E+04
274	Hydroxyeicosatetraenoic acid	[M-H]-	C20H32O3	319.22787	319.22711	-2.4	1.7E+05
275	4-(3,5-Diphenylcyclohexyl)phenol	[M-H]-	C24H24O	327.17544	327.17621	2.4	1.8E+05
276	2,2'-(1-phenyl-1H-1,2,4-triazole-3,5-diyl)bis-phenol	[M-H]-	C20H15N3O2	328.10915	328.10868	-1.4	4.9E+04
277	2-[3-Ethyl-5-(4-methoxyphenyl)-1H-pyrazol-4-yl]phenol	[M+Cl]-	C18H18N2O2	329.10623	329.10623	0.0	6.2E+04
278	{(3S)-3-[(2Z)-pent-2-en-1-yl]oxiran-2-ylidene}undec-9-enoic acid	[M+K]+	C18H28O3	331.16700	331.16634	-2.0	1.6E+05
279	4-Prenylresveratrol	[M+K]+	C19H20O3	335.10440	335.10396	-1.3	3.3E+05
280	Trihexyphenidyl	[M+Cl]-	C20H31NO	336.20997	336.20904	-2.8	4.2E+04
281	11R,12S-epoxy-8-hydroxy-5Z,9E,14Z-eicosatrienoic acid	[M+H]+	C20H32O4	337.23734	337.23647	-2.6	4.7E+05
282	Dehydrophytosphingosine	[M+Na]+	C18H37NO3	338.26656	338.26731	2.2	8.2E+05
283	N1-Amidinostreptamine 6-phosphate	[M+K]+	C7H17N4O7P	339.04665	339.04668	0.1	1.4E+05
284	1-Dehydro-15alpha-hydroxytestololactone	[M+Na]+	C19H24O4	339.15668	339.15750	2.4	2.9E+05
285	4-Carboxy-4'-sulfoazobenzene	[M+Cl]-	C13H10N2O5S	341.00044	341.00060	0.5	1.3E+05
286	Lactose	[M-H]-	C12H22O11	341.10894	341.10952	1.7	2.2E+05
287	(+)-12-Isocopalene-15,16-dial	[M+K]+	C20H30O2	341.18774	341.18831	1.7	1.4E+05
288	N,N,N-trimethyl-sphingosine	[M-H]-	C21H44NO2	341.32993	341.32938	-1.6	4.8E+04
289	13-HETE	[M+Na]+	C20H31O3	342.21654	342.21606	-1.4	1.4E+05
290	10,13-Eicosadiynoic acid	[M+K]+	C20H32O2	343.20339	343.20407	2.0	5.4E+05
291	Thiamin monophosphate	[M+H]+	C12H18N4O4PS	346.08591	346.08622	0.9	1.1E+05
292	O-Arachidonoyl Ethanolamine	[M+H]+	C22H37NO2	348.28971	348.28878	-2.7	1.5E+05
293	2-O-(6-Phospho-alpha-mannosyl)-D-glycerate	[M+H]+	C9H17O12P	349.05304	349.05319	0.4	1.3E+05
294	Heneicosanoic acid	[M+Na]+	C21H42O2	349.30770	349.30811	1.2	1.3E+05
295	(4E,8E,9Me-d19:2)sphingosine	[M+K]+	C19H37NO2	350.24559	350.24493	-1.9	1.3E+05
296	17-Propyl-5alpha-androst-2-en-17beta-ol	[M+Cl]-	C22H36O	351.24602	351.24668	1.9	1.2E+05
297	Dopamine glucuronide	[M+Na]+	C14H19NO8	352.10029	352.10005	-0.7	1.4E+05
298	12a-Hydroxydolineone	[M+H]+	C19H12O7	353.06558	353.06501	-1.6	1.7E+05
299	Testosterone acetate	[M+Na]+	C21H30O3	353.20872	353.20965	2.6	1.0E+06
300	2,5-Diamino-6-(5'-phosphoribosylamino)-4-pyrimidineone	[M+H]+	C9H16N5O8P	354.08093	354.08077	-0.4	8.5E+05
301	11,12,15S-trihydroxy-5Z,8Z,13E-eicosatrienoic acid	[M+H]+	C20H34O5	355.24790	355.24707	-2.3	2.4E+05
302	Succinyl sulfathiazole	[M+H]+	C13H13N3O5S2	356.03694	356.03706	0.3	1.4E+05

#	Putative Annotation (M)[a]	Ion	Formula	Theor. *m/z*[b]	Exp. *m/z*[c]	ppm[d]	Peak_Height
303	(5,12,13-Triaza-indeno[1,2-b]anthracen-13-yl)-acetic acid ethylester	[M+H]+	C22H17N3O2	356.13935	356.13887	-1.4	1.5E+05
304	Trihydroxy-trimethylpyranodihydrochalcone	[M+H]+	C21H24O5	357.16965	357.17067	2.9	1.6E+05
305	Tetrahydrodeoxycorticosterone	[M+Na]+	C21H34O3	357.24002	357.24082	2.3	1.7E+05
306	Nicotinate D-ribonucleotide	[M+Na]+	C11H15NO9P	359.03766	359.03861	2.6	1.2E+05
307	12(S)-HETE	[M+K]+	C20H32O3	359.19830	359.19919	2.5	2.0E+05
308	Estra-1,3,5(10),16-tetraen-3-ol benzoate	[M+H]+	C25H26O2	359.20056	359.20041	-0.4	1.8E+05
309	2',4',6'-Trihydroxy-3'-prenyldihydrochalcone	[M+Cl]-	C20H22O4	361.12121	361.12124	0.1	3.7E+04
310	Oleoyl glycine	[M+Na]+	C20H37NO3	362.26656	362.26700	1.2	1.5E+05
311	MG(0:0/16:0/0:0)	[M+Cl]-	C19H38O4	365.24641	365.24677	1.0	5.1E+05
312	N-Acetyl-6-O-L-fucosyl-D-glucosamine	[M-H]-	C14H25NO10	366.14057	366.13985	-2.0	3.3E+04
313	1-pentyl-sn-glycero-3-phosphocholine	[M+K]+	C13H30NO6P	366.14423	366.14517	2.6	1.3E+05
314	Steryl sulfate	[M+K]+	C17H28O4S	367.13399	367.13504	2.9	2.7E+05
315	trans-3-Hydroxycotinine glucuronide	[M+H]+	C16H20N2O8	369.12924	369.12981	1.5	1.4E+05
316	4'-O-Methylxanthohumol	[M+H]+	C22H24O5	369.16965	369.16992	0.7	1.7E+05
317	12-Keto-leukotriene B4	[M+Cl]-	C20H30O4	369.18381	369.18434	1.4	4.9E+04
318	Dihydroneopterin phosphate	[M+Cl]-	C9H14N5O7P	370.03249	370.03356	2.9	6.2E+04
319	7-O-Acetylsalutaridinol	[M-H]-	C21H25NO5	370.16600	370.16694	2.6	1.4E+05
320	(2S)-5,7,4'-Trihydroxy-3'-methoxy-6-(1,1-dimethylallyl)flavanone	[M+H]+	C21H22O6	371.14891	371.14951	1.6	3.1E+05
321	Methyl-2-alpha-L-fucopyranosyl-beta-D-galactoside	[M+Cl]-	C13H24O10	375.10635	375.10726	2.4	3.8E+04
322	2',4'-Dihydroxy-6'-methoxy-3'-prenylchalcone	[M+K]+	C21H22O4	377.11497	377.11455	-1.1	1.4E+05
323	3,6-Dimethoxy-19-norpregna-1,3,5,7,9-pentaen-20-one	[M+K]+	C22H26O3	377.15135	377.15035	-2.7	2.5E+05
324	5,7-Dihydroxyflavone 7-benzoate	[M+Na]+	C22H14O5	381.07334	381.07419	2.2	3.1E+05
325	3-Oxopregn-4-ene-20beta-carboxaldehyde dioxime	[M+Na]+	C22H34N2O2	381.25125	381.25164	1.0	2.5E+05
326	C17 Sphinganine-1-phosphate	[M+H]+	C17H41N2O5P	385.28259	385.28317	1.5	1.3E+05
327	5-Hydroxy-3',4'-methylenedioxy-6",6"-dimethylpyrano[2",3":7,8]isoflavone	[M+Na]+	C21H16O6	387.08391	387.08355	-0.9	2.7E+05
328	N-Acetylmuramoyl-Ala	[M+Na]+	C14H24N2O9	387.13740	387.13689	-1.3	4.5E+05
329	11,15,19-trimethyl-5Z,9Z,17Z-eicosatrienoic acid	[M+K]+	C23H40O2	387.26599	387.26621	0.6	1.3E+05
330	6-Deoxyerythronolide	[M+H]+	C21H38O6	387.27412	387.27387	-0.6	1.4E+05
331	Acetyl adenylate	[M-H]-	C12H16N5O8P	388.06637	388.06661	0.6	3.6E+04
332	12-oxo-20-dihydroxy-leukotriene B4	[M+Na]+	C20H29O6	388.18563	388.18536	-0.7	1.9E+05
333	N-Acetylneuraminate 9-phosphate	[M+H]+	C11H20NO12P	390.07959	390.07986	0.7	1.6E+05
334	Rehmaionoside A	[M+H]+	C19H34O8	391.23264	391.23315	1.3	2.3E+05
335	(22E)-3alpha,12alpha-Dihydroxy-5beta-chol-22-en-24-oic Acid	[M+H]+	C24H38O4	391.28429	391.28491	1.6	9.8E+05
336	N-stearoyl serine	[M+Na]+	C21H41NO4	394.29278	394.29383	2.7	1.4E+05
337	(5E)-isovitamin D2	[M-H]-	C28H44O	395.33194	395.33248	1.4	2.6E+04
338	PC(4:0/4:0)	[M-H]-	C16H32NO8P	396.17928	396.17974	1.2	6.1E+04
339	Tris(butoxyethyl)phosphate	[M-H]-	C18H39O7P	397.23606	397.23678	1.8	2.5E+05

#	Putative Annotation (M)[a]	Ion	Formula	Theor. *m/z*[b]	Exp. *m/z*[c]	ppm[d]	Peak_Height
340	1'H-5alpha-Androst-2-eno[3,2-b]indol-17beta-ol	[M+Cl]-	C25H33NO	398.22562	398.22534	-0.7	2.3E+05
341	Bisphenol A dimethacrylate	[M+Cl]-	C23H24O4	399.13686	399.13787	2.5	9.9E+04
342	16,16-dimethyl-PGA1	[M+Cl]-	C22H36O4	399.23076	399.23120	1.1	5.8E+04
343	1-Arachidonoylglycerol	[M+Na]+	C23H38O4	401.26623	401.26539	-2.1	1.6E+05
344	trans-3-Hydroxycotinine glucuronide	[M+Cl]-	C16H20N2O8	403.09137	403.09189	1.3	1.1E+05
345	1-Hydroxy-2-(beta-D-glucosyloxy)-9,10-anthraquinone	[M+H]+	C20H18O9	403.10236	403.10190	-1.1	2.3E+05
346	(3'R,4'R)-3'-Epoxyangeloyloxy-4'-acetoxy-3',4'-dihydroseselin	[M+H]+	C21H22O8	403.13874	403.13865	-0.2	2.1E+05
347	16Z-pentacosenoic acid	[M+Na]+	C25H48O2	403.35465	403.35453	-0.3	2.6E+05
348	N-palmitoyl phenylalanine	[M+H]+	C25H41NO3	404.31592	404.31712	3.0	1.6E+05
349	Salicin 6-phosphate	[M+K]+	C13H19O10P	405.03474	405.03494	0.5	1.4E+05
350	12a-Hydroxypachyrrhizone	[M+Na]+	C20H14O8	405.05809	405.05717	-2.3	1.9E+05
351	1alpha,5alpha-Epidithio-17a-oxa-D-homoandrostan-3,17-dione	[M+K]+	C19H26O3S2	405.09550	405.09605	1.4	1.9E+05
352	Cinncassiol A	[M+Na]+	C20H30O7	405.18837	405.18869	0.8	2.2E+05
353	1alpha,25-dihydroxy-21-nor-20-oxavitamin D3	[M+H]+	C25H40O4	405.29994	405.29919	-1.8	3.0E+05
354	Leu-leu-tyr	[M+H]+	C21H33N3O5	408.24930	408.24934	0.1	2.2E+05
355	3beta-(1-Pyrrolidinyl)-5alpha-pregnane-11,20-dione	[M+Na]+	C25H39NO2	408.28730	408.28694	-0.9	3.9E+05
356	17-oxo-20Z-hexacosenoic acid	[M+H]+	C26H48O3	409.36762	409.36867	2.6	1.7E+05
357	(22E)-3alpha,12alpha-Dihydroxy-5beta-chol-22-en-24-oic Acid	[M+Na]+	C24H38O4	413.26623	413.26744	2.9	4.7E+05
358	PA(14:1(9Z)/0:0)	[M+Cl]-	C17H33O7P	415.16579	415.16489	-2.2	7.3E+04
359	5,3',4'-Trihydroxy-3,7-dimethoxy-6-prenylflavone	[M+Na]+	C22H22O7	421.12577	421.12580	0.1	1.5E+05
360	17-methyl-tetracosanoic acid	[M+K]+	C25H50O2	421.34424	421.34323	-2.4	4.4E+05
361	Quassin	[M+Cl]-	C22H28O6	423.15799	423.15791	-0.2	1.1E+05
362	Deoxygomisin A	[M+Na]+	C23H28O6	423.17781	423.17872	2.2	1.6E+05
363	Plakinamine A	[M+H]+	C29H46N2	423.37338	423.37403	1.5	1.5E+05
364	PC(O-6:0/O-6:0)	[M-H]-	C20H44NO6P	424.28335	424.28293	-1.0	1.3E+05
365	5,2',5'-Trihydroxy-3,7,8-trimethoxyflavone 2'-acetate	[M+Na]+	C20H18O9	425.08430	425.08443	0.3	1.9E+05
366	Prostaglandin D2-1-glyceryl ester	[M-H]-	C23H38O7	425.25448	425.25503	1.3	4.9E+05
367	5,12-Dihydroxanthommatin	[M+H]+	C20H15N3O8	426.09319	426.09352	0.8	1.6E+05
368	PGF2alpha-dihydroxypropanylamine	[M-H]-	C23H41NO6	426.28611	426.28616	0.1	3.0E+05
369	PA(15:1(9Z)/0:0)	[M+Cl]-	C18H35O7P	429.18144	429.18258	2.7	6.3E+04
370	PA(18:4/0:0)	[M-H]-	C21H35O7P	429.20476	429.20450	-0.6	4.8E+04
371	Diisooctyl phthalate	[M+K]+	C24H38O4	429.24017	429.24014	-0.1	2.7E+06
372	Dihydroxy-campestenone	[M-H]-	C28H48O3	431.35307	431.35285	-0.5	3.5E+04
373	1-Benzyl-7,8-dimethoxy-3-phenyl-3H-pyrazolo[3,4-c]isoquinoline	[M+K]+	C25H21N3O2	434.12654	434.12538	-2.7	1.4E+05
374	Varanic acid	[M-H]-	C26H44O5	435.31160	435.31251	2.1	1.5E+05
375	Stigmasterol	[M+Na]+	C29H48O	435.35974	435.36103	3.0	2.1E+05
376	N-(1-methyl-2-hydroxy-2-phenyl-ethyl) arachidonyl amine	[M-H]-	C29H43NO2	436.32210	436.32264	1.2	8.3E+04
377	4alpha-methyl-5alpha-cholestan-3beta-ol	[M+Cl]-	C28H50O	437.35557	437.35453	-2.4	7.0E+04

#	Putative Annotation (M)[a]	Ion	Formula	Theor. *m/z*[b]	Exp. *m/z*[c]	ppm[d]	Peak_Height
378	(17Z)-1alpha,25-dihydroxy-26,27-dimethyl-17,20,22,22,23,23-hexadehydrovitamin D3	[M+H]+	C29H42O3	439.32067	439.32014	-1.2	1.4E+05
379	N-(2-hydroxy-2S-methyl-ethyl)-16,16-dimethyl-5Z,8Z,11Z,14Z-docosatetraenoyl amine	[M+Na]+	C27H47NO2	440.34990	440.35071	1.8	1.5E+05
380	N-linolenoyl-glutamine	[M+Cl]-	C23H38N2O4	441.25256	441.25275	0.4	3.7E+05
381	Palmitoyl glucuronide	[M+Na]+	C22H42O7	441.28227	441.28354	2.9	2.2E+05
382	(10E)-19-fluorovitamin D3 / (10E)-19-fluorocholecalciferol	[M+K]+	C27H43FO	441.29295	441.29322	0.6	1.4E+05
383	(20S)-20-cyclopropyl-1alpha,25-dihydroxy-16,17-didehydro-21-norvitamin D3	[M+H]+	C29H44O3	441.33632	441.33651	0.4	1.4E+05
384	(-)-Epicatechin 3-O-gallate	[M+H]+	C22H18O10	443.09727	443.09716	-0.3	2.1E+05
385	(23R)-23-Hydroxy-3,7-dioxo-5beta-cholan-24-oic Acid	[M+K]+	C24H36O5	443.21943	443.21882	-1.4	1.7E+05
386	1,25-Dihydroxyvitamin D3-26,23-lactone	[M-H]-	C27H40O5	443.28030	443.28104	1.7	6.3E+04
387	15alpha-hydroxycholestane	[M+K]+	C27H48O2	443.32859	443.32864	0.1	2.0E+05
388	Dihydrofolate	[M+H]+	C19H21N7O6	444.16261	444.16301	0.9	2.0E+05
389	N-oleoyl glutamine	[M+Cl]-	C23H42N2O4	445.28386	445.28476	2.0	4.6E+04
390	19-(3-methyl-butanoyloxy)-villanovane-13alpha,17-diol	[M+Na]+	C25H42O5	445.29245	445.29111	-3.0	1.8E+05
391	19Z-octacosenoic acid	[M+Na]+	C28H54O2	445.40160	445.40120	-0.9	1.6E+05
392	Kaempferol 3,7-di-O-sulfate	[M+H]+	C15H10O12S2	446.96864	446.96948	1.9	1.4E+05
393	5,7,2',6'-Tetrahydroxyflavone 2'-O-glucoside	[M-H]-	C21H20O11	447.09328	447.09309	-0.4	1.5E+05
394	4-epi-clavulone II	[M+H]+	C25H34O7	447.23773	447.23865	2.1	1.7E+05
395	24-isopropenyl-22E-dehydrocholesterol	[M+Na]+	C30H48O	447.35974	447.36046	1.6	2.2E+05
396	17beta-Estradiol 17-(beta-D-glucuronide)	[M+H]+	C24H31O8	448.20917	448.20947	0.7	1.5E+05
397	2-Geranyl-3,4,2',4'-tetrahydroxydihydrochalcone	[M+K]+	C25H30O5	449.17248	449.17256	0.2	2.3E+05
398	3alpha,12alpha,25-trihydroxy-5beta-cholestan-7-one	[M+H]+	C28H48O4	449.36254	449.36281	0.6	2.9E+05
399	1alpha-fluoro-25-hydroxy-16,17,23,23,24,24-hexadehydrovitamin D3	[M+K]+	C27H37FO2	451.24092	451.24163	1.6	1.7E+05
400	4,2'-Dihydroxy-4',6'-dimethoxychalcone 4-glucoside	[M+H]+	C23H26O10	463.15987	463.16107	2.6	3.8E+05
401	17-[[3-(1-Pyrrolidinyl)propyl]imino]androst-5-en-3beta-ol acetate	[M+Na]+	C28H44N2O2	463.32950	463.32963	0.3	2.4E+05
402	1-tetradecanoyl-glycero-3-phosphoserine	[M+H]+	C20H40NO9P	470.25135	470.25225	1.9	1.9E+05
403	19-oxo-22Z-octacosenoic acid	[M+Cl]-	C28H52O3	471.36105	471.36060	-1.0	5.9E+04
404	11-trans-LTE4; 5S-hydroxy-6R-(S-cysteinyl)-7E,9E,11E14Z-eicosatetraenoic acid	[M+Cl]-	C23H37NO5S	474.20865	474.20945	1.7	1.1E+05
405	Glucolimnanthin	[M+K]+	C15H20NO10S2	477.01602	477.01518	-1.8	1.6E+05
406	5,7,2'-Trihydroxy-8-methoxyflavone 7-glucuronide	[M+H]+	C22H20O12	477.10275	477.10200	-1.6	1.7E+05
407	1-(9Z-tetradecenoyl)-glycero-3-phospho-(1'-sn-glycerol)	[M+Na]+	C20H39O9P	477.22239	477.22134	-2.2	2.0E+05
408	1-O-all-trans-retinoyl-beta-glucuronic acid	[M+H]+	C26H36O8	477.24829	477.24778	-1.1	1.5E+05
409	Parsonsine	[M+K]+	C23H37NO7	478.22016	478.21962	-1.1	1.4E+05
410	3'-Geranyl-3,4,2',4'-tetrahydroxy-6'-methoxydihydrochalcone	[M+K]+	C26H32O6	479.18305	479.18325	0.4	1.8E+05
411	(24R)-24-Methylcycloarta-25-en-3-beta-ol	[M+K]+	C31H52O	479.36498	479.36614	2.4	1.8E+05

#	Putative Annotation (M)[a]	Ion	Formula	Theor. *m/z*[b]	Exp. *m/z*[c]	ppm[d]	Peak_Height
412	23Z-dotriacontenoic acid	[M+H]+	C32H62O2	479.48226	479.48095	-2.7	1.6E+05
413	N-Acetyl-leukotriene E4	[M-H]-	C25H39NO6S	480.24253	480.24266	0.3	1.6E+05
414	13'-hydroxy-alpha-tocopherol	[M+Cl]-	C29H50O3	481.34540	481.34581	0.9	4.0E+04
415	S-Decyl glutathione	[M+Cl]-	C20H37N3O6S	482.20971	482.20932	-0.8	1.8E+05
416	Lankacidin C	[M+Na]+	C25H33NO7	482.21492	482.21616	2.6	2.0E+05
417	3,4,2',3',4',6',alpha-Heptahydroxychalcone 2'-glucoside	[M+H]+	C21H22O13	483.11332	483.11449	2.4	1.7E+05
418	Doxycycline	[M+K]+	C22H24N2O8	483.11643	483.11676	0.7	1.9E+05
419	11alpha-ethyl-1alpha,25-dihydroxyvitamin D3 / 11alpha-ethyl-1alpha,25-dihydroxycholecalciferol;	[M+K]+	C29H48O3	483.32350	483.32261	-1.9	1.9E+05
420	17-hydroxyandrostane-3-glucuronide	[M+H]+	C25H40O9	485.27451	485.27460	0.2	1.6E+05
421	C17 sphingosine-1-phosphocholine	[M+Cl]-	C22H47N2O5P	485.29166	485.29091	-1.6	5.1E+04
422	(22R)-1alpha,22,25-trihydroxy-26,27-dimethyl-23,23,24,24-tetradehydro-24a,24b-dihomovitamin D3	[M+H]+	C31H48O4	485.36254	485.36237	-0.3	2.3E+05
423	(24R)-25-fluoro-1alpha,24-dihydroxy-24-methylvitamin D3	[M+K]+	C28H45FO3	487.29843	487.29815	-0.6	3.2E+05
424	2-(8-[3]-ladderane-octanyl)-sn-glycero-3-phosphoethanolamine	[M+H]+	C25H46NO6P	488.31355	488.31357	0.0	3.9E+05
425	Dotriacontanol	[M+Na]+	C32H66O	489.50059	489.50187	2.6	1.7E+05
426	L-2-Aminoadipate adenylate	[M+H]+	C16H23N6O10P	491.12860	491.12717	-2.9	1.7E+05
427	1",2"-Dihydro-8-hydroxyisopentanyl-2'-methoxy-4'-O-methylalpinumisoflavone	[M+Na]+	C27H32O7	491.20402	491.20256	-3.0	2.0E+05
428	1-hydroxy-2-hexadecanoyl-sn-glycero-3-phosphoethanolamine	[M+K]+	C21H44NO7P	492.24870	492.24787	-1.7	2.0E+05
429	Patuletin 3,3'-di-O-sulfate	[M+H]+	C16H12O14S2	492.97412	492.97300	-2.3	1.5E+05
430	Tetrahydrogeranylgeranyl diphosphate	[M+K]+	C20H40O7P2	493.18809	493.18694	-2.3	1.5E+05
431	(24RS)-28,28,28-trifluoro-25-hydroxyvitamin D2	[M+K]+	C28H41F3O2	505.26902	505.27023	2.4	1.7E+05
432	Deoxyuridine triphosphate	[M+K]+	C9H15N2O14P3	506.93677	506.93551	-2.5	1.5E+05
433	(25S)-3-oxo-12beta-acetoxy-cholest-1,4-dien-26-oic acid	[M+Na]+	C30H44O5	507.30810	507.30897	1.7	1.7E+05
434	Luciferyl sulfate	[M+Na]+	C26H21N3O5S	510.10941	510.11039	1.9	1.6E+05
435	omega-Carboxy-N-acetyl-LTE4	[M-H]-	C25H37NO8S	510.21671	510.21611	-1.2	7.0E+04
436	PS(18:4/0:0)	[M-H]-	C24H40NO9P	516.23679	516.23543	-2.6	1.0E+05
437	PA(20:4(5Z,8Z,11Z,14Z)e/2:0)	[M+K]+	C25H43O7P	525.23780	525.23773	-0.1	1.8E+05
438	5,3',4'-Trihydroxy-7-methoxy-4-phenylcoumarin 5-O-(6"-acetyl)-galactoside	[M+Na]+	C24H24O12	527.11600	527.11560	-0.8	1.7E+05
439	Makisterone A	[M+Cl]-	C28H46O7	529.29376	529.29250	-2.4	1.6E+05
440	1-tridecanoyl-sn-glycero-3-phospho-(1'-myo-inositol)	[M+H]+	C22H43O12P	531.25649	531.25510	-2.6	3.2E+05
441	1-(9Z,12Z-octadecadienoyl)-glycero-3-phospho-(1'-sn-glycerol)	[M+Na]+	C24H45O9P	531.26934	531.27035	1.9	2.0E+05
442	2',4',6',3,4-Pentahydroxy-3'-geranyl-5-prenyldihydrochalcone	[M+K]+	C30H38O6	533.23000	533.23092	1.7	3.4E+05
443	5-O-(Indol-3-ylacetyl-myo-inositol) D-galactoside	[M+Cl]-	C22H29NO12	534.13838	534.13813	-0.5	5.1E+04
444	(S)-N-[3-(3,4-Methylenedioxyphenyl)-2-(acetylthio)methyl-1-oxoprolyl]-(S)-alanine benzyl ester	[M+Cl]-	C25H28N2O7S	535.13112	535.13139	0.5	1.6E+05

#	Putative Annotation (M)[a]	Ion	Formula	Theor. *m/z*[b]	Exp. *m/z*[c]	ppm[d]	Peak_Height
445	Lipoxin D4	[M+Na]+	C25H40N2O7S	535.24484	535.24641	2.9	2.0E+05
446	Neurosporaxanthin	[M+K]+	C35H46O2	537.31294	537.31150	-2.7	2.8E+05
447	1-(9Z-tetradecenoyl)-2-(9Z-hexadecenoyl)-sn-glycerol	[M+H]+	C33H60O5	537.45135	537.45235	1.9	1.8E+05
448	Thiamin triphosphate	[M+Cl]-	C12H20N4O10P3S	539.98070	539.98128	1.1	5.6E+04
449	Chitotriose	[M+K]+	C18H35N3O13	540.18015	540.18092	1.4	2.6E+05
450	10-Deoxygeniposide tetraacetate	[M+H]+	C25H32O13	541.19157	541.19067	-1.7	2.2E+05
451	1-(1Z,12Z-nonadecadienyl)-sn-glycero-3-phosphocholine	[M+Na]+	C27H54NO6P	542.35810	542.35693	-2.1	2.9E+05
452	dolichyl diphosphate	[M+Na]+	C25H46O7P2	543.26110	543.25991	-2.2	8.3E+05
453	Thiamin triphosphate	[M+K]+	C12H20N4O10P3S	543.97446	543.97521	1.4	4.4E+05
454	2-deoxy-20-hydroxyecdysone 22-phosphate	[M+H]+	C27H45O9P	545.28740	545.28713	-0.5	6.0E+05
455	1-(9Z,12Z-heptadecadienoyl)-glycero-3-phosphoserine	[M+K]+	C23H42NO9P	546.22288	546.22197	-1.7	3.2E+05
456	(24R)-11alpha,20,24-trihydroxyecdysone	[M+Cl]-	C27H44O9	547.26793	547.26777	-0.3	9.1E+04
457	Hydroxyphthioceranic acid (C36)	[M-H]-	C36H72O3	551.54087	551.54008	-1.4	1.0E+05
458	(D-Ala(2)-mephe(4)-gly-ol(5))enkephalin	[M+K]+	C26H35N5O6	552.22189	552.22332	2.6	1.8E+05
459	2-Aminoethylphosphocholate	[M+K]+	C26H46NO7P	554.26435	554.26408	-0.5	2.0E+05
460	PG(P-20:0/0:0)	[M+Cl]-	C26H53O8P	559.31721	559.31560	-2.9	7.6E+04
461	Cholesterol glucuronide	[M+H]+	C33H54O7	563.39423	563.39365	-1.0	2.1E+05
462	PI(16:1/0:0)	[M-H]-	C25H47O12P	569.27324	569.27401	1.4	4.0E+04
463	5,4'-Dihidroxy-7,8,2',3'-tetramethoxy flavone 5-glucoside	[M+Cl]-	C25H28O13	571.12239	571.12368	2.3	5.0E+04
464	6,7,3',4'-Tetrahydroxyaurone 6-(2'',4'',6''-triacetylglucoside)	[M+H]+	C27H26O14	575.13953	575.13913	-0.7	2.2E+05
465	Coroglaucigenin-3-o-alpha-L-rhamnopyranoside	[M+K]+	C29H44O9	575.26169	575.26270	1.8	3.4E+05
466	Tetrahydroxy-4-phenylcoumarin 5-O-apiosyl-(1->6)-glucoside	[M-H]-	C26H28O15	579.13554	579.13396	-2.7	4.8E+04
467	Formamidopyrimidine nucleoside triphosphate	[M+K]+	C10H18N5O15P3	579.96438	579.96443	0.1	1.9E+05
468	PS(20:3/0:0)	[M+Cl]-	C26H46NO9P	582.26042	582.26144	1.8	1.4E+05
469	(3Z)-Phycocyanobilin	[M-H]-	C33H38N4O6	585.27186	585.27279	1.6	6.0E+04
470	Cholic acid glucuronide	[M+H]+	C30H48O11	585.32694	585.32561	-2.3	2.3E+05
471	DG(P-14:0/18:1)	[M+Cl]-	C35H66O4	585.46551	585.46709	2.7	8.9E+04
472	3-O-acetylecdysone 2-phosphate	[M+H]+	C29H47O10P	587.29796	587.29773	-0.4	2.1E+05
473	Catechin 3,7,-di-O-galate	[M-H]-	C29H22O14	593.09368	593.09492	2.1	7.9E+04
474	1-heneicosanoyl-glycero-3-phospho-(1'-sn-glycerol)	[M+K]+	C27H55O9P	593.32153	593.32059	-1.6	2.3E+05
475	5-O-beta-D-Mycaminosyltylonolide	[M-H]-	C31H51NO10	596.34402	596.34530	2.2	9.7E+04
476	N-Acetyl-leu-leu-leu-tyr-amide	[M+K]+	C29H47N5O6	600.31579	600.31738	2.6	2.2E+05
477	dolichyl beta-D-glucosyl phosphate	[M-H]-	C31H55O9P	601.35109	601.35183	1.2	4.9E+04
478	Cyanidin 3-(2''-galloylgalactoside)	[M+H]+	C28H25O15	602.12662	602.12723	1.0	1.9E+05
479	5,7,3'-Trihydroxy-6.8,4'-trimethoxyflavone 5-(6''-acetylglucoside)	[M+K]+	C26H28O14	603.11107	603.11124	0.3	2.5E+05
480	PS(22:4(7Z,10Z,13Z,16Z)/0:0)	[M+Cl]-	C28H48NO9P	608.27607	608.27778	2.8	1.6E+05
481	Tetraacetoxy-cholest-5-en-3alpha-ol	[M-H]-	C35H54O9	617.36951	617.37059	1.8	1.5E+05
482	1-hexadecanoyl-2-valeryl-sn-glycero-3-phosphocholine	[M+K]+	C29H58NO8P	618.35316	618.35255	-1.0	4.1E+05

#	Putative Annotation (M)[a]	Ion	Formula	Theor. *m/z*[b]	Exp. *m/z*[c]	ppm[d]	Peak_Height
483	1-(6-[5]-ladderane-hexanoyl)-2-(8-[3]-ladderane-octanyl)-sn-glycerol	[M-H]-	C41H64O4	619.47318	619.47418	1.6	4.0E+04
484	Bacteriorubuxanthin	[M+K]+	C41H58O2	621.40684	621.40807	2.0	1.8E+05
485	Adenosine tetraphosphate	[M+Cl]-	C10H17N5O16P4	621.93148	621.93246	1.6	6.3E+04
486	Cer(d18:0/22:0)	[M+H]+	C40H81NO3	624.62892	624.62879	-0.2	2.1E+05
487	1-heptadecanoyl-glycero-3-phospho-(1'-myo-inositol)	[M+K]+	C26H51O12P	625.27497	625.27378	-1.9	3.2E+05
488	Delphinidin 3-O-(6-caffeoyl-beta-D-glucoside)	[M+H]+	C30H27O15	628.14227	628.14271	0.7	2.1E+05
489	Kaempferol 3-(3"-acetyl-alpha-L-arabinofuranoside)-7-rhamnoside	[M+Na]+	C28H30O15	629.14769	629.14780	0.2	2.8E+05
490	7"-O-Phosphohygromycin	[M+Na]+	C20H38N3O16P	630.18819	630.18727	-1.5	1.9E+05
491	PI(18:1(9Z)/0:0)	[M+Cl]-	C27H51O12P	633.28122	633.28053	-1.1	1.0E+05
492	PS(13:0/12:0)	[M-H]-	C31H60NO10P	636.38821	636.38860	0.6	3.6E+04
493	1-dodecanoyl-2-(9Z-tetradecenoyl)-glycero-3-phospho-(1'-sn-glycerol)	[M+H]+	C32H61O10P	637.40751	637.40684	-1.1	2.3E+05
494	1-dodecanoyl-2-(9Z,12Z-heptadecadienoyl)-glycero-3-phosphoethanolamine	[M+H]+	C34H64NO8P	646.44423	646.44593	2.6	1.7E+05
495	2-Octaprenyl-6-hydroxyphenol	[M-H]-	C46H70O2	653.53031	653.53184	2.4	4.9E+04
496	Z-Arg-Arg-NHMec	[M+Cl]-	C30H39N9O6	656.27173	656.27151	-0.3	1.2E+05
497	1-dodecanoyl-2-nonadecanoyl-glycero-3-phosphate	[M+Na]+	C34H67O8P	657.44658	657.44603	-0.8	2.0E+05
498	Neocarzinostatin chromophore	[M-H]-	C35H33NO12	658.19300	658.19228	-1.1	3.9E+04
499	Delphinidin 3-glucoside-5-(6-acetylglucoside)	[M+H]+	C29H33O18	670.17397	670.17342	-0.8	2.2E+05
500	bacteriohopane-32,33-diol-34,35-dicarbamate	[M+K]+	C37H64N2O6	671.43960	671.43808	-2.3	2.0E+05
501	1-(6Z,9Z,12Z,15Z-octadecatetraenoyl)-2-(5Z,8Z,11Z,14Z,17Z-eicosapentaenoyl)-sn-glycerol	[M+K]+	C41H62O5	673.42288	673.42448	2.4	2.4E+05
502	1-tetradecanoyl-2-(1-enyl-1Z,11Z-octadecadienoyl)-sn-glycero-3-phosphoethanolamine	[M+H]+	C37H72NO7P	674.51192	674.51040	-2.2	2.8E+05
503	1-tridecanoyl-2-(5Z,8Z,11Z,14Z-eicosatetraenoyl)-glycero-3-phosphate	[M+Na]+	C36H63O8P	677.41528	677.41548	0.3	3.8E+05
504	PA(15:0/20:5)	[M-H]-	C38H65O8P	679.43443	679.43522	1.2	7.4E+04
505	Cer(d18:0/26:0)	[M+H]+	C44H89NO3	680.69152	680.69101	-0.8	2.6E+05
506	Noranhydroicaritin 3-rhamnosyl-(1->2)-rhamnoside	[M+K]+	C32H38O14	685.18932	685.18970	0.6	2.7E+05
507	1-(8-[3]-ladderane-octanoyl-2-(8-[3]-ladderane-octanyl)-sn-glycerol	[M+Cl]-	C43H70O4	685.49681	685.49531	-2.2	4.2E+04
508	1-dodecanoyl-2-(7Z,10Z,13Z,16Z-docosatetraenoyl)-glycero-3-phosphate	[M+Na]+	C37H65O8P	691.43093	691.43019	-1.1	3.4E+05
509	DG(17:0/22:5)/0:0)	[M+Cl]-	C42H72O5	691.50738	691.50815	1.1	1.6E+05
510	2-Octaprenyl-6-methoxyphenol	[M+Na]+	C47H72O2	691.54245	691.54215	-0.4	2.5E+05
511	1-tetradecanoyl-2-(4Z,7Z,10Z,13Z,16Z,19Z-docosahexaenoyl)-glycero-3-phosphate	[M+H]+	C39H65O8P	693.44898	693.45027	1.9	3.7E+05
512	1-docosanoyl-glycero-3-phospho-(1'-myo-inositol)	[M+K]+	C31H61O12P	695.35322	695.35302	-0.3	3.0E+05
513	1,2-di(9Z-tetradecenoyl)-rac-glycero-3-phosphoserine	[M+Na]+	C34H62NO10P	698.40035	698.40146	1.6	3.4E+05
514	1-(1Z-hexadecenyl)-2-tridecanoyl-glycero-3-phosphoserine [1Z-alkenyl,2-acylglycerophosphoserines	[M+Na]+	C35H68NO9P	700.45239	700.45240	0.0	3.6E+05

#	Putative Annotation (M)[a]	Ion	Formula	Theor. *m/z*[b]	Exp. *m/z*[c]	ppm[d]	Peak_Height
515	1-pentadecanoyl-2-(5Z,8Z,11Z,14Z,17Z-eicosapentaenoyl)-glycero-3-phosphate	[M+Na]+	C38H65O8P	703.43093	703.43133	0.6	2.8E+05
516	PA(15:1/22:6)	[M-H]-	C40H65O8P	703.43443	703.43466	0.3	2.2E+05
517	1-tridecanoyl-2-(6Z,9Z,12Z-octadecatrienoyl)-glycero-3-phospho-(1'-sn-glycerol)	[M+H]+	C37H67O10P	703.45446	703.45591	2.1	4.7E+05
518	Limocitrin 3,7-diglucoside	[M+Cl]-	C29H34O18	705.14392	705.14492	1.4	1.8E+05
519	Guanosine 3'-diphosphate 5'-triphosphate	[M+Na]+	C10H18N5O20P5	705.91254	705.91436	2.6	4.5E+05
520	1-dodecanoyl-2-(5Z,8Z,11Z,14Z-eicosatetraenoyl)-glycero-3-phosphoethanolamine	[M+Na]+	C37H66NO8P	706.44183	706.44234	0.7	4.9E+05
521	1-dodecanoyl-2-(4Z,7Z,10Z,13Z,16Z,19Z-docosahexaenoyl)-glycero-3-phosphoethanolamine	[M+H]+	C39H66NO8P	708.45988	708.46077	1.3	2.8E+05
522	2,3-Bis(3-hydroxytetradecanoyl)-beta-D-glucosaminyl 1-phosphate	[M+H]+	C34H66NO12P	712.43954	712.43999	0.6	4.5E+05
523	1-tridecanoyl-2-docosanoyl-glycero-3-phosphate	[M+Na]+	C38H75O8P	713.50918	713.50926	0.1	2.3E+05
524	1-(6Z,9Z,12Z,15Z-octadecatetraenoyl)-2-(1-enyl-hexadecenoyl)-sn-glycero-3-phosphoethanolamine	[M+Na]+	C39H70NO7P	718.47821	718.47763	-0.8	4.3E+05
525	1-pentadecanoyl-2-(8Z,11Z,14Z-eicosatrienoyl)-glycero-3-phosphate	[M+K]+	C38H69O8P	723.43617	723.43573	-0.6	3.8E+05
526	1-(9Z,12Z-octadecadienoyl)-2-(15Z-tetracosanoyl)-sn-glycerol	[M+Na]+	C45H82O5	725.60545	725.60670	1.7	2.3E+05
527	Alpha-Tetrasaccharide	[M+Cl]-	C26H45NO20	726.22289	726.22309	0.3	3.1E+04
528	1-dodecanoyl-2-(9Z-heptadecenoyl)-glycero-3-phosphoserine	[M+K]+	C35H66NO10P	730.40559	730.40362	-2.7	5.3E+05
529	1-octadecyl-2-(5Z,8Z,11Z,14Z,17Z-eicosapentaenoyl)-glycero-3-phosphate	[M+Na]+	C41H73O7P	731.49861	731.49905	0.6	4.6E+05
530	PS(12:0/20:1)	[M-H]-	C38H72NO10P	732.48211	732.48394	2.5	4.2E+04
531	Pentacarboxylporphyrin I	[M+Cl]-	C37H38N4O10	733.22820	733.22695	-1.7	3.3E+04
532	N-(heptadecanoyl)-sphing-4-enine-1-phosphocholine	[M+Na]+	C40H81N2O6P	739.57245	739.57466	3.0	2.3E+05
533	1-(1Z-hexadecenyl)-2-(9Z-hexadecenoyl)-glycero-3-phosphoserine	[M+Na]+	C38H72NO9P	740.48369	740.48468	1.3	5.6E+05
534	PS(12:0/18:0)	[M+Cl]-	C36H70NO10P	742.44314	742.44228	-1.2	9.2E+04
535	1-tetradecanoyl-2-docosanoyl-glycero-3-phosphate	[M+K]+	C39H77O8P	743.49877	743.49809	-0.9	5.6E+05
536	1-tridecanoyl-2-(5Z,8Z,11Z,14Z,17Z-eicosapentaenoyl)-glycero-3-phospho-(1'-sn-glycerol)	[M+Na]+	C39H67O10P	749.43641	749.43745	1.4	2.5E+05
537	1,2-di(4Z,7Z,10Z,13Z,16Z,19Z-docosahexaenoyl)-rac-glycerol	[M+K]+	C47H68O5	751.46983	751.47144	2.1	6.0E+05
538	PA(16:0/22:6)	[M+Cl]-	C41H69O8P	755.44241	755.44451	2.8	3.5E+04
539	1-dodecanoyl-2-(11Z-eicosenoyl)-glycero-3-phosphoserine	[M+Na]+	C38H72NO10P	756.47860	756.47869	0.1	3.8E+05
540	1-(10Z,13Z,16Z-docosatrienoyl)-2-(4Z,7Z,10Z,13Z,16Z,19Z-docosahexaenoyl)-sn-glycerol	[M+K]+	C47H74O5	757.51678	757.51806	1.7	4.6E+05
541	Oceanalin A	[M+Na]+	C41H72N2O9	759.51300	759.51308	0.1	6.1E+05
542	(3R,2'S)-Myxol 2'-(2,4-di-O-methyl-alpha-L-fucoside)	[M+H]+	C48H70O7	759.51943	759.52111	2.2	3.6E+05
543	Quercetin 3-(2''-galoylrutinoside)	[M-H]-	C34H34O20	761.15707	761.15744	0.5	6.0E+04

#	Putative Annotation (M)[a]	Ion	Formula	Theor. *m/z*[b]	Exp. *m/z*[c]	ppm[d]	Peak_Height
544	1-(6-[5]-ladderane-hexanyl)-2-(8-[3]-ladderane-octanyl)-sn-glycero-3-phospho-(1'-sn-glycerol)	[M+H]+	C44H73O8P	761.51158	761.51330	2.3	7.3E+05
545	1-(6Z,9Z,12Z-octadecatrienoyl)-2-(1-enyl-1Z,11Z-octadecadienoyl)-sn-glycero-3-phosphoethanolamine	[M+K]+	C41H74NO7P	762.48345	762.48488	1.9	3.2E+05
546	CerP(d18:1/24:1)	[M+Cl]-	C42H82NO6P	762.55738	762.55560	-2.3	1.7E+05
547	PI(12:0/17:2)	[M-H]-	C38H69O13P	763.44030	763.43961	-0.9	4.5E+05
548	1-hexadecanoyl-2-(7Z,10Z,13Z,16Z-docosatetraenoyl)-glycero-3-phosphate	[M+K]+	C41H73O8P	763.46747	763.46564	-2.4	9.1E+05
549	1,2-di-(3Z,6Z,9Z,12Z,15Z-octadecapentaenoyl)-3-O-beta-D-galactosyl-sn-glycerol	[M+H]+	C45H66O10	767.47287	767.47413	1.6	3.7E+05
550	PA(P-18:0/22:6)	[M+Cl]-	C43H73O7P	767.47879	767.48054	2.3	1.1E+05
551	1-tetradecanoyl-2-(8Z,11Z,14Z-eicosatrienoyl)-glycero-3-phospho-(1'-sn-glycerol)	[M+Na]+	C40H73O10P	767.48336	767.48482	1.9	2.6E+05
552	PC(O-16:0/17:0)	[M+Cl]-	C41H84NO7P	768.56794	768.56581	-2.8	4.5E+04
553	(3R,2'S)-Myxol 2'-alpha-L-fucoside	[M+K]+	C46H66O7	769.44401	769.44560	2.1	2.8E+05
554	1-hexadecyl-2-(9Z-octadecenoyl)-glycero-3-phosphoserine [1-alkyl,2-acylglycerophosphoserines	[M+Na]+	C40H78NO9P	770.53064	770.53258	2.5	2.3E+05
555	Leucomycin A5	[M+H]+	C39H65NO14	772.44778	772.44650	-1.7	2.2E+05
556	1-(8-[5]-ladderane-octanoyl)-2-(8-[3]-ladderane-octanyl)-sn-glycerophosphoethanolamine	[M+H]+	C45H74NO7P	772.52757	772.52870	1.5	4.7E+05
557	1-dodecanoyl-2-(7Z,10Z,13Z,16Z-docosatetraenoyl)-glycero-3-phosphocholine	[M+Na]+	C42H76NO8P	776.52008	776.52107	1.3	5.0E+05
558	1-(6-[3]-ladderane-hexanoyl)-2-(8-[3]-ladderane-octanyl)-sn-glycero-3-phospho-(1'-sn-glycerol)	[M+H]+	C44H73O9P	777.50650	777.50675	0.3	4.0E+05
559	5,7,3'-Trihydroxy-4'-methoxyflavanone 7-(2,6-dirhamnosylglucoside)	[M+Na]+	C34H44O19	779.23690	779.23481	-2.7	2.1E+05
560	3-O-Sulfogalactosylceramide (d18:1/16:0)	[M+H]+	C40H77NO11S	780.52901	780.52703	-2.5	2.4E+05
561	PG(15:1(9Z)/22:2)	[M-H]-	C43H79O10P	785.53381	785.53215	-2.1	7.6E+04
562	1-tridecanoyl-2-(4Z,7Z,10Z,13Z,16Z,19Z-docosahexaenoyl)-glycero-3-phosphocholine	[M+Na]+	C43H74NO8P	786.50443	786.50641	2.5	4.9E+05
563	1-(6-[3]-ladderane-hexanoyl)-2-(8-[3]-ladderane-octanyl)-sn-glycerophosphocholine	[M+H]+	C46H78NO7P	788.55887	788.55785	-1.3	2.1E+05
564	1-(9Z-octadecenoyl)-2-(7Z,10Z,13Z,16Z-docosatetraenoyl)-glycero-3-phosphate	[M+K]+	C43H75O8P	789.48312	789.48469	2.0	2.7E+05
565	1-(9Z-octadecenoyl)-2-(13Z,16Z-docosadienoyl)-glycero-3-phosphate	[M+K]+	C43H79O8P	793.51442	793.51268	-2.2	8.7E+05
566	PA(22:2/22:6)	[M-H]-	C47H77O8P	799.52833	799.52875	0.5	7.7E+04
567	N-(docosanoyl)-hexadecasphinganine-1-phosphocholine	[M+K]+	C43H89N2O6P	799.60898	799.60955	0.7	4.0E+05
568	1-(11Z-eicosenoyl)-2-(7Z,10Z,13Z,16Z-docosatetraenoyl)-glycero-3-phosphate	[M+Na]+	C45H79O8P	801.54048	801.53905	-1.8	5.0E+05

#	Putative Annotation (M)[a]	Ion	Formula	Theor. m/z^b	Exp. m/z^c	ppm[d]	Peak_Height
569	1-hexadecyl-2-(4Z,7Z,10Z,13Z,16Z,19Z-docosahexaenoyl)-glycero-3-phospho-(1'-sn-glycerol)	[M+Na]+	C44H77O9P	803.51974	803.52040	0.8	4.1E+05
570	1-(1Z-hexadecenyl)-2-(9Z,12Z-heptadecadienoyl)-glycero-3-phospho-(1'-myo-inositol)	[M+H]+	C42H77O12P	805.52254	805.52485	2.9	3.3E+05
571	1-octadecanoyl-2-(12S-hydroxy-5Z,8Z,10E,14Z-eicosatetraenoyl)-sn-glycero-3-phosphoethanolamine	[M+Na]+	C43H78NO9P	806.53064	806.53282	2.7	6.3E+05
572	1-pentadecanoyl-2-(7Z,10Z,13Z,16Z-docosatetraenoyl)-glycero-3-phospho-(1'-sn-glycerol)	[M+Na]+	C43H77O10P	807.51466	807.51416	-0.6	8.5E+05
573	PG(P-20:0/17:2)	[M+Cl]-	C43H81O9P	807.53122	807.53130	0.1	1.1E+05
574	FAD	[M+Na]+	C27H33N9O15P2	808.14636	808.14464	-2.1	2.2E+05
575	1-(2E,6E-phytadienyl)-2-(2E,6E-phytadienyl)-sn-glycero-3-phosphocholine	[M-H]-	C48H92NO6P	808.65895	808.65717	-2.2	4.6E+04
576	PC(P-20:0/19:1)	[M-H]-	C47H92NO7P	812.65386	812.65627	3.0	3.4E+04
577	PE(16:0/22:6)(14OH))	[M+Cl]-	C43H74NO9P	814.47952	814.48006	0.7	1.9E+05
578	1-hexadecyl-2-docosanoyl-glycero-3-phospho-(1'-sn-glycerol)	[M+Na]+	C44H89O9P	815.61364	815.61581	2.7	2.7E+05
579	1-tetradecanoyl-2-(4Z,7Z,10Z,13Z,16Z,19Z-docosahexaenoyl)-sn-glycero-3-phosphocholine	[M+K]+	C44H76NO8P	816.49401	816.49391	-0.1	4.9E+05
580	1-tridecanoyl-2-(5Z,8Z,11Z,14Z-eicosatetraenoyl)-glycero-3-phospho-(1'-myo-inositol)	[M+H]+	C42H73O13P	817.48616	817.48707	1.1	9.3E+05
581	1-tetradecanoyl-2-(4Z,7Z,10Z,13Z,16Z,19Z-docosahexaenoyl)-glycero-3-phosphoserine	[M+K]+	C42H70NO10P	818.43689	818.43728	0.5	2.7E+05
582	1-hexadecanoyl-2-(4Z,7Z,10Z,13Z,16Z-docosapentaenoyl)-sn-glycero-3-phospho-(1'-sn-glycerol)	[M+Na]+	C44H77O10P	819.51466	819.51643	2.2	5.3E+05
583	1-hexadecyl-2-(9Z,12Z-octadecadienoyl)-glycero-3-phospho-(1'-myo-inositol)	[M+H]+	C43H81O12P	821.55384	821.55178	-2.5	4.2E+05
584	5,10-Methenyltetrahydromethanopterin	[M+Cl]-	C31H44N6O16P	822.22454	822.22428	-0.3	6.7E+04
585	1-(9Z-pentadecenoyl)-2-(13Z,16Z-docosadienoyl)-glycero-3-phosphoserine	[M+Na]+	C43H78NO10P	822.52556	822.52402	-1.9	4.6E+05
586	1-dodecanoyl-2-(4Z,7Z,10Z,13Z,16Z,19Z-docosahexaenoyl)-glycero-3-phospho-(1'-myo-inositol)	[M+H]+	C43H71O13P	827.47051	827.47160	1.3	5.3E+05
587	1-(9Z-pentadecenoyl)-2-(4Z,7Z,10Z,13Z,16Z,19Z-docosahexaenoyl)-glycero-3-phosphocholine	[M+K]+	C45H76NO8P	828.49401	828.49494	1.1	4.5E+05
588	1-octadecanoyl-2-(14-hydroxy-4Z,7Z,10Z,12E,16Z,19Z-docosahexaenoyl)-sn-glycero-3-phosphoethanolamine	[M+Na]+	C45H78NO9P	830.53064	830.52843	-2.7	3.1E+05
589	3-O-Sulfogalactosylceramide (d18:1/18:0)	[M+Na]+	C42H81NO11S	830.54225	830.54395	2.0	5.0E+05
590	1-hexadecyl-2-(9Z-heptadecenoyl)-glycero-3-phospho-(1'-myo-inositol)	[M+Na]+	C42H81O12P	831.53579	831.53580	0.0	7.4E+05
591	1-heneicosanoyl-2-(7Z,10Z,13Z,16Z-docosatetraenoyl)-glycero-3-phosphate	[M+K]+	C46H83O8P	833.54572	833.54694	1.5	5.5E+05

#	Putative Annotation (M)[a]	Ion	Formula	Theor. *m/z*[b]	Exp. *m/z*[c]	ppm[d]	Peak_Height
592	PS(18:3/20:5)	[M+Cl]-	C44H70NO10P	838.44314	838.44104	-2.5	6.4E+04
593	1-pentadecanoyl-2-(9Z-octadecenoyl)-3-hexadecanoyl-glycerol	[M+Na]+	C52H98O6	841.72556	841.72732	2.1	2.3E+05
594	1-(11Z-docosenoyl)-2-(7Z,10Z,13Z,16Z-docosatetraenoyl)-glycero-3-phosphate	[M+K]+	C47H83O8P	845.54572	845.54718	1.7	7.1E+05
595	1-octadecanoyl-2-(15-oxo-5Z,8Z,11Z,13E-eicosatetraenoyl)-sn-glycero-3-phosphoethanolamine	[M+Na]+	C46H82NO9P	846.56194	846.56293	1.2	5.3E+05
596	Acetyl-CoA	[M+K]+	C23H38N7O17P3S	848.08893	848.08887	-0.1	2.7E+05
597	1-tridecanoyl-2-(13Z,16Z-docosadienoyl)-glycero-3-phospho-(1'-myo-inositol)	[M+H]+	C44H81O13P	849.54876	849.54874	0.0	1.7E+06
598	PG(20:1/22:2)	[M-H]-	C48H89O10P	855.61206	855.61081	-1.5	8.9E+04
599	1-eicosyl-2-(4Z,7Z,10Z,13Z,16Z,19Z-docosahexaenoyl)-glycero-3-phospho-(1'-sn-glycerol)	[M+Na]+	C48H85O9P	859.58234	859.58122	-1.3	4.7E+05
600	1-octadecanoyl-2-(4Z,7Z,10Z,13Z,16Z,19Z-docosahexaenoyl)-sn-glycero-3-phospho-(1'-sn-glycerol)	[M+K]+	C46H79O10P	861.50424	861.50266	-1.8	1.6E+06
601	1-(9Z-nonadecenoyl)-2-(7Z,10Z,13Z,16Z-docosatetraenoyl)-glycero-3-phospho-(1'-sn-glycerol)	[M+Na]+	C47H83O10P	861.56161	861.56198	0.4	2.7E+05
602	1-(docosatetraenoyl)-2-(docosahexaenoyl)-sn-glycero-3-phosphoethanolamine	[M+Na]+	C49H78NO8P	862.53573	862.53442	-1.5	9.9E+05
603	SM(d18:1/25:0)	[M+Cl]-	C48H97N2O6P	863.67783	863.67659	-1.4	7.2E+04
604	1-hexadecanoyl-2-heptadecanoyl-3-(5Z,8Z,11Z,14Z,17Z-eicosapentaenoyl)-sn-glycerol	[M+H]+	C56H98O6	867.74362	867.74524	1.9	3.9E+05
605	PI(15:1(9Z)/22:4)	[M-H]-	C46H79O13P	869.51855	869.51902	0.5	1.6E+05
606	1-eicosanoyl-2-(4Z,7Z,10Z,13Z,16Z,19Z-docosahexaenoyl)-glycero-3-phospho-(1'-sn-glycerol)	[M+Na]+	C48H83O10P	873.56161	873.56115	-0.5	8.7E+05
607	PI(O-18:0/20:3)	[M-H]-	C47H87O12P	873.58624	873.58804	2.1	3.0E+04
608	TG(16:0/17:2/18:3)	[M+Cl]-	C54H94O6	873.67444	873.67405	-0.5	2.3E+05
609	1-(9Z-hexadecenoyl)-2-(9Z,12Z,15Z-octadecatrienoyl)-3-(5Z,8Z,11Z,14Z,17Z-eicosapentaenoyl)-sn-glycerol	[M+H]+	C57H92O6	873.69667	873.69851	2.1	2.7E+05
610	1-octadecanoyl-2-(7Z,10Z,13Z,16Z-docosatetraenoyl)-glycero-3-phosphoserine	[M+K]+	C46H82NO10P	878.53079	878.53336	2.9	7.1E+05
611	PC(P-20:0/21:0)	[M+Cl]-	C49H98NO7P	878.67749	878.67555	-2.2	1.5E+05
612	1-(11Z-eicosenoyl)-2-(13Z,16Z-docosadienoyl)-glycero-3-phospho-(1'-sn-glycerol)	[M+Na]+	C48H89O10P	879.60856	879.60764	-1.0	8.7E+05
613	PS(18:0/22:0)	[M+Cl]-	C46H90NO10P	882.59964	882.60112	1.7	4.9E+04
614	1-hexadecyl-2-(5Z,8Z,11Z,14Z-eicosatetraenoyl)-glycero-3-phospho-(1'-myo-inositol)	[M+K]+	C45H81O12P	883.50972	883.51148	2.0	3.0E+05
615	1-hexadecyl-2-(11Z-eicosenoyl)-glycero-3-phospho-(1'-myo-inositol) [1-alkyl,2-acylglycerophosphoinositols	[M+K]+	C45H87O12P	889.55667	889.55678	0.1	9.8E+05

#	Putative Annotation (M)[a]	Ion	Formula	Theor. *m/z*[b]	Exp. *m/z*[c]	ppm[d]	Peak_Height
616	1-heptadecanoyl-2-(5Z,8Z,11Z,14Z-eicosatetraenoyl)-sn-glycero-3-phospho-(1'-myo-inositol)	[M+H]+	C46H84NO13P	890.57530	890.57686	1.7	7.2E+05
617	PG(22:1(11Z)/20:4(5Z,8Z,11Z,14Z))	[M+K]+	C48H85O10P	891.55120	891.54867	-2.8	2.3E+06
618	PC(20:3(8Z,11Z,14Z)/22:4(7Z,10Z,13Z, 16Z))	[M+K]+	C50H86NO8P	898.57226	898.57044	-2.0	5.8E+05
619	PS(22:0/22:2)	[M-H]-	C50H94NO10P	898.65426	898.65568	1.6	8.5E+04
620	PI(O-18:0/19:1)	[M+Cl]-	C46H89O12P	899.57857	899.58102	2.7	8.7E+04
621	2-Methylacetoacetyl-CoA	[M+K]+	C26H42N7O18P3S	904.11515	904.11253	-2.9	7.4E+05
622	(3'-sulfo)Galbeta-Cer(d18:0/2-OH-24:1)	[M-H]-	C48H91NO12S	904.61892	904.61897	0.1	3.9E+04
623	PC(20:3(8Z,11Z,14Z)/22:1(11Z))	[M+K]+	C50H92NO8P	904.61922	904.62022	1.1	2.9E+05
624	PG(21:0/22:4(7Z,10Z,13Z,16Z))	[M+K]+	C49H89O10P	907.58250	907.58427	2.0	6.5E+05
625	PG(21:0/22:0)	[M+Cl]-	C49H97O10P	911.65134	911.65134	0.0	2.2E+04
626	PI(P-18:0/20:2(11Z,14Z))	[M+K]+	C47H87O12P	913.55667	913.55665	0.0	6.5E+05
627	PI(16:0/22:1(11Z))	[M+Na]+	C47H89O13P	915.59330	915.59551	2.4	6.8E+05
628	PS(22:1(11Z)/22:2(13Z,16Z))	[M+Na]+	C50H92NO10P	920.63511	920.63564	0.6	5.6E+05
629	2-Aminobenzoyl-CoA	[M-H]-	C28H41N8O17P3S	921.12172	921.12282	1.2	1.0E+05
630	PI(22:1(11Z)/17:1(9Z))	[M+Na]+	C48H89O13P	927.59330	927.59191	-1.5	3.2E+05
631	Glc-GP(18:0/20:0)	[M+K]+	C47H91O13P	933.58289	933.58437	1.6	1.2E+06
632	Bilirubin beta-diglucuronide	[M-H]-	C45H52N4O18	935.32038	935.32314	3.0	8.4E+04
633	1,2-Di-(9Z,12Z,15Z-octadecatrienoyl)-3-(Galactosyl-alpha-1-6-Galactosyl-beta-1)-glycerol	[M+H]+	C51H84O15	937.58830	937.58747	-0.9	3.8E+05
634	PI(O-20:0/20:0)	[M+K]+	C49H97O12P	947.63492	947.63562	0.7	1.4E+06
635	PI(22:6/21:0)	[M-H]-	C52H89O13P	951.59680	951.59549	-1.4	7.8E+04
636	3-O-Sulfogalactosylceramide (d18:1/26:1(17Z))	[M+K]+	C50H95NO11S	956.62574	956.62528	-0.5	7.3E+05
637	PI(21:0/22:2(13Z,16Z))	[M+H]+	C52H97O13P	961.67396	961.67402	0.1	9.6E+05
638	PI(20:1(11Z)/22:4(7Z,10Z,13Z,16Z))	[M+Na]+	C51H89O13P	963.59330	963.59065	-2.8	2.6E+05
639	(3S)-Citryl-CoA	[M+Na]+	C27H42N7O22P3S	964.12087	964.12004	-0.9	2.7E+05
640	(2E)-Dodecenoyl-CoA	[M+Na]+	C33H52N7O17P3S	966.22454	966.22373	-0.8	2.8E+05
641	Trihexosylceramide (d18:1/12:0)	[M+H]+	C48H89NO18	968.61524	968.61264	-2.7	5.4E+05
642	DAT(16:0/22:0(2Me[S],4Me[S]))	[M+K]+	C52H98O13	969.66390	969.66591	2.1	4.5E+05
643	Benzylsuccinyl-CoA	[M+Cl]-	C32H46N7O19P3S	992.14760	992.14713	-0.5	7.9E+04
644	PIP2(16:0/18:0)	[M-H]-	C43H85O19P3	997.48251	997.47996	-2.6	5.8E+04

[a]Cer: Ceramide; GalCer: Galactosylceramide; GlcCer: Glucosylceramide; Glc-GP: phosphatidylglucose; LacCer: Lactosylceramide; MG: Monoacylglycerol; DAT: Acyltrehaloses; DG: Diacylglycerol; TG: Triacylglycerol; MGDG: Monoacyldiacylglycerol; PA: Phosphatidic acid; PC: Phosphatidylcholine; PE: Phosphatidylethanolamine; PG: Glycerophospholipids; PI: Phosphatidylinositol; PIP2: phosphatidylinositol bisphosphate; PS: Phosphatidylserine; SM: Sphingomyelin.
[b]Theor. stands for calculated exact mass to charge ratio.
[c]Exp. stands for experimental *m*/*z* value.
[d]The error expressed in parts per million (ppm).
